# Study on the Metabonomics Mechanism of Mongolian Medical Andai Therapy on Healthy People

**DOI:** 10.1155/2022/1364408

**Published:** 2022-06-20

**Authors:** QiLa Sa, LiHong Bao, YaGeTu Hu, Haihua Bai, AGuLa Bo

**Affiliations:** ^1^Medicine Innovation Center for Nationalities, Inner Mongolia Medical University, Hohhot 010110, China; ^2^Medical College, Inner Mongolia Minzu University, Tongliao 028000, China; ^3^Inner Mongolia International Mongolian Medicine Hospital, Hohhot 010065, China; ^4^Life and Food Science College, Inner Mongolia Minzu University, Tongliao 028000, China; ^5^Baotou Medical College, Inner Mongolia University of Science and Technology, Baotou 014060, China

## Abstract

Andai therapy is a traditional therapy combining body, mind, and language with Mongolian characteristics. In the form of singing and dancing, it is widely popular among people of all ages in Mongolian areas of Inner Mongolia. According to Mongolian medicine, Heyi is one of the three elements of human body, and it can maintain life activities, promote blood circulation, and improve the functions of the sensory and mental consciousness. Andai therapy stimulates the whole body nerves and Heyi through music and dance, improves Heyi and blood operation, strengthens physique, improves immunity, effectively promotes physical and mental health, and plays a role in preventing and treating diseases. *Objective*. In this study, gas chromatography-mass spectrometry (GC-MS) was used to explore the mechanism of Andai therapy, so as to provide a new research direction for taking targeted prevention and treatment measures for diseases. *Methods*. Using gas chromatography-mass spectrometry (GC-MS) on all its cases baseline plasma to the targeted metabonomics testing, the differential metabolites of the experimental group (receiving Andai therapy) and control group (without receiving Andai therapy), analysis-related metabolite function, and screening of metabolites and related pathways through adjusting mechanism to explore the related factors are compared, to study the mechanism of the influence of Mongolian medical Andai therapy on the metabolism of different healthy people. *Results*. The differences in metabolic numbers between the experimental group and the control group are 114, such as cyclohexylamine chlorinated acid, 2,4-2 aminobutyric acid bitter almond alcohol, l-methyl inosine, 2-picolinate, and 2-hydroxy-2-glutaric acid metabolite content of the control group that are significantly higher than the experimental group, experimental group's other substance content is significantly higher than that of the control group, and two groups' metabolite content was obviously different. The number of differential metabolites between the female experimental group and the female control group was 119, and the number of differential metabolites between the male experimental group and the male control group was 48.

## 1. Introduction

“Andai” is a treatment method for psychosomatic diseases spread in Kulun Banner, Tongliao City, Inner Mongolia [1]. According to the literature records, traditional Andai is a healing ritual performed by Andai therapists for the purpose of curing the physical discomfort and mental disorders of married and unmarried women between the ages of 18 and 25 years [2]. With the development of The Times, the new Andai therapy mainly refers to the integration of Andai's self-cultivation methods, such as dance, music, sports, and language, so as to achieve the principle of body and mind integration in Mongolian medicine. Books such as Da Cheng of Traditional Mongolian Medicine Therapy [3] and Traditional Mongolian Medical Therapy [4] record in detail the indications and effects of Mongolian medical Andai therapy and reflect its medical value in treating diseases. Andai therapy is a combination of psychology, music, dance, and sports therapy with distinctive national characteristics. After more than 300 years of development and continuous enrichment and innovation, on May 20, 2006, the Mongolian Andai dance was approved by The State Council to be included in the first batch of the national intangible cultural heritage list [5]. At present, research studies on Andai mainly focus on literature, art, sociology, singing and dancing, and other disciplines. Andai has its unique efficacy in health promotion and disease prevention, but there is no systematic and complete research report so far. Therefore, this study discusses the mechanism of Mongolian medicine Andai therapy. In order to give full play to the characteristics and advantages of Mongolian medicine Andai therapy in health promotion and disease prevention and treatment by combining modern medicine, as we all know, exercise is good for health. Exercise can not only improve immunity, enhance cardiopulmonary function, and improve cognitive ability but also bring immediate changes to metabolism and other physiological processes. On May 28, 2020, a team of researchers led by Michael P. Snyder and Francois Haddad at the Stanford University School of Medicine provided the first comprehensive analysis of the effects of exercise on the human body at the molecular level from the perspectives of proteomics, metabolomics, lipidomics, and transcriptome [6]. The study also found that in insulin-resistant volunteers, changes in key biological pathways such as immune response and lipid, carbohydrate, and amino acid metabolism were less severe after exercise than in healthy subjects because they could not properly process glucose metabolism. Paolo's team studied the changes in skeletal muscle metabolites in mice at different exercise time points, which showed that exercise immediately changed carbohydrate metabolism after getting up [7]. It can be seen that exercise has a significant impact on the basic metabolism of the human body, and dancing, as a common way of exercise, plays an equally important role in the metabolism of the body. In this study, people who do Andai dance and those who do not dance Andai dance are studied by metabonomics technology, and the internal mechanism of Mongolian Andai dance bringing external changes to the crowd is analyzed.

## 2. Materials and Methods

### 2.1. Sample Selection

In this study, 24 healthy volunteers from Kulun Banner, Tongliao City, Inner Mongolia, the “hometown of Andai,” who have participated in Andai dance for the past two years (practicing Andai dance for no less than 150 min per week and no less than 30 min each time) and 24 healthy volunteers who have not participated in any intervention were selected. Inclusion criteria were as follows: normal healthy persons aged 55–65 years screened from the biobank of the Metabolic Disease Research Center. The healthy people were divided into four groups: the experimental group (male and female group) receiving Mongolian medical Andai therapy and the control group (male and female group) without Mongolian medical Andai therapy (Table 1).

### 2.2. The Experimental Operation

The samples stored at −80°C were thawed at room temperature. The samples were transferred from 80 *μ*L to 1.5 mL EP tubes, 20 *μ*L internal standard (L-2-chlorophenylalanine, 0.3 mg/mL, methanol) was added, and the samples were shaken by vortex for 10 s. About 240 *μ*L methanol-acetonitrile (V : V = 2 : 1) was added, and the mixture was oscillated for 30 s. With ultrasonic extraction with an ice bath for 10 min, stand for 30 min at −20°C, and centrifuged for 10 min (13000 rpm, 4°C), 150 *μ*L of supernatant was taken into a glass-derived flask. The quality control (QC) sample is prepared by mixing the extract of all samples with equal volume, and the volume of each QC is the same as the sample; the samples were dried with a freeze-concentrated centrifugal dryer. About 80 *μ*L methoxylamine hydrochloride pyridine solution (15 mg/mL) was added to the glass-derived vials, and the reaction was carried out in an oscillating incubator at 37°C for 90 min after 2 min of vortex oscillation. 80 *μ*L of BSTFA (with 1% TMCS) and 20 *μ*L n-hexane was added into the mixture, which was vortexed vigorously for 2 min and then derivatized at 70°C for 60 min. After the samples were removed, they were placed at room temperature for 30 min for GC-MS analysis.

Chromatographic conditions were as follows: DB-5MS capillary column (30 m × 0.25 mm × 0.25 *μ*m, Agilent J&W Scientific, Folsom, CA, USA) was carried by high purity helium (purity not less than 99.999%) with a flow rate of 1.0 mL/min. The inlet temperature is 260°C. The injection volume was 1 *μ*L, and the solvent delay was 5 min. The programmed temperature was as follows: the initial temperature of the column temperature box is 60°C and kept for 0.5 min; ramped to 125°C at a rate of 8°C/min; to 210°C at a rate of 5°C/min; to 270°C at a rate of 10°C/min; to 305°C at a rate of 20°C/min; and finally held at 305°C for 5 min. Mass spectrometry conditions were as follows: electron-bombarded ion source (EI), ion source temperature 230°C, quadrupole temperature 150°C, and electron energy 70 eV. SCAN mode was as follows: full SCAN mode (SCAN) and quality SCAN range: *M*/*Z* 50–500.

### 2.3. The Data Analysis

Metabolic Fingerprinting: In order to identify and identify as many metabolites as possible in biological samples, the most appropriate elution gradient and mass spectrometry parameters are selected according to the optimal response value and signal-to-noise ratio of samples in positive and negative modes, so as to obtain and identify more abundant chromatographic peaks. The plasma samples of the experimental group and the control group were examined by metabonomics to obtain the preliminary metabolic fingerprint. The UPLC-Q-TOF-MS total ion flow diagrams with abundant chromatographic peak information were obtained in total ion flow mode. After the original data are processed by Mass Profiler Professional, the excel data are exported and imported into SIMCA-P 13.0 for discriminant analysis. In the total ion flow mode, the clustering effect between each group and the separation degree between the experimental group and the control group were analyzed to determine whether there were significant differences in metabolite expression and confirm that there were no abnormalities in the grouping. Then, the original data of all groups were extracted by molecular characteristics and the integration was investigated. Mass Profiler Professional software was used for further data analysis and processing. The multiple changes were set as two times, *P* < 0.01, and the target compound was finally determined and derived. The target compounds were further qualitative analyzed by Qualitative Analysis B.07.00 to identify biomarkers. Then, the mass to charge ratio, retention time, related metabolism, and content changes in different target metabolites were analyzed.

Principal Component Analysis (PCA): The principal component analysis is an analysis method that deletes redundant and repeated variables (closely related variables) on the basis of original variables and establishes as few new variables as possible, so as to determine whether there is a correlation between new variables. As a process of information enrichment, the PCA analysis can help us further determine whether there are significant differences among the four groups.

Volcanic Map Analysis: Fold change ≥2.0 was used as the standard. The horizontal axis is log2 (FC), and the vertical axis is -log10 (*P* value). Each point represents a sample. The two lines parallel to the *Y* axis are *X* = 1 and *X* = −1, respectively. Meanwhile, parallel to the *X* axis, there is a dotted line *Y* = 1.30, namely, −log10 (0.05). Points above the dotted line represent samples with significance <0.05.

Differential Metabolite Analysis: In the analysis of differential metabolites in samples, the heat map is mainly used, which is generally used for two purposes: first, it visually presents the global expression changes in multiple genes in various samples; the second is the clustering relationship of multiple genes or genes.

Channel Path Analysis: All differential metabolites with defined structures were input into MetaboAnalyst4.0 and associated with KEGGC (https://www.kegg.Jp/) database for pathway analysis, and it was found that differential metabolites were mainly involved in multiple metabolic pathways.

## 3. Results

Metabolic fingerprint analysis showed no difference between the four groups in the total ion flow diagram.

### 3.1. Principal Component Analysis (PCA)

**Figure 1:**
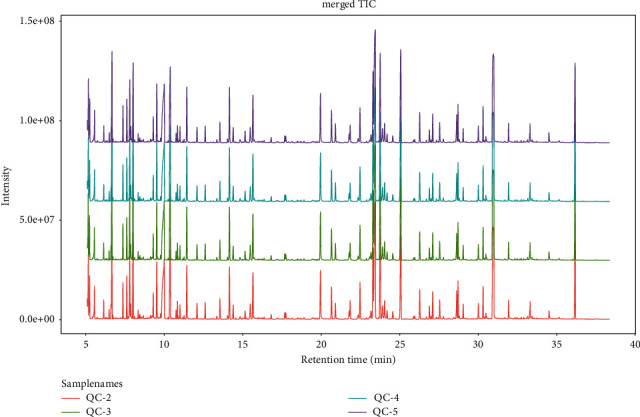
Merged TIC—metabolic fingerprint.

**Figure 2:**
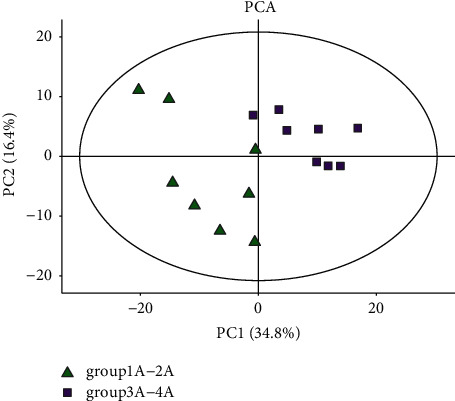
The PCA diagram of the whole sample.

**Figure 3:**
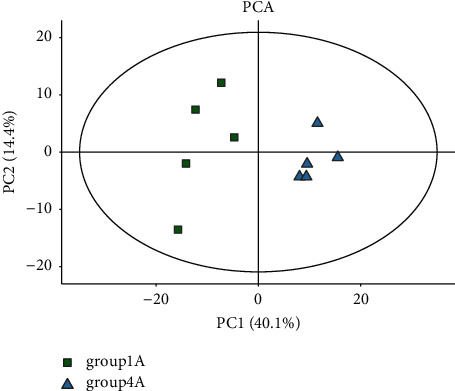
The PCA diagram of female sample.

**Figure 4:**
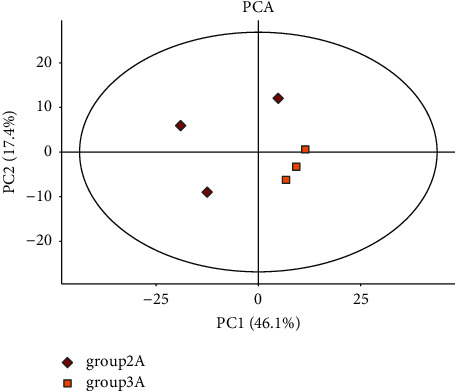
The PCA diagram of male sample.

Through the TIC superposition diagram (Figure 1), it was found that there were no significant differences among the four groups: the control group (female), the control group (male), the experimental group (female), and the experimental group (male). In order to further determine whether there were differences among the four groups, the principal component analysis (PCA) was conducted in this part. According to Figure 2, the experimental group samples were mainly concentrated in the second and third quadrants, while the control group samples were mainly concentrated in the first quadrant, indicating significant differences between the experimental group and the control group. It can be seen from Figure 3 that the data of the control group (female) are mainly concentrated in the first and fourth quadrants, while the data of the control group (female) are mainly concentrated in the second and third quadrants, indicating that there are significant differences between the experimental group and the control group in female samples. As can be seen from Figure 4, in male samples, the experimental group was mainly concentrated in the first, second, and third quadrants, while the control group was mainly concentrated in the fourth quadrant, indicating significant differences between the experimental group and the control group in male samples.

### 3.2. Volcanic Map Analysis

**Figure 5:**
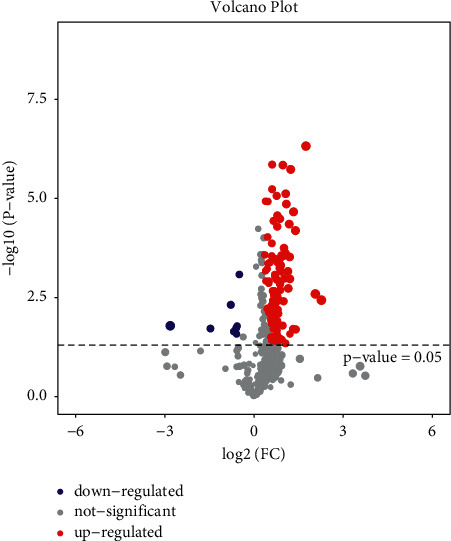
Full-sample comparison.

**Figure 6:**
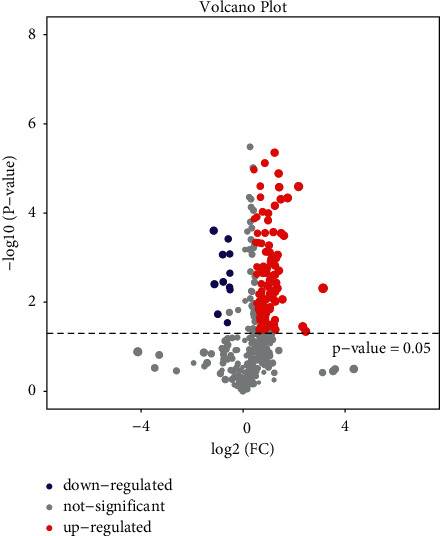
Female sample comparison.

**Figure 7:**
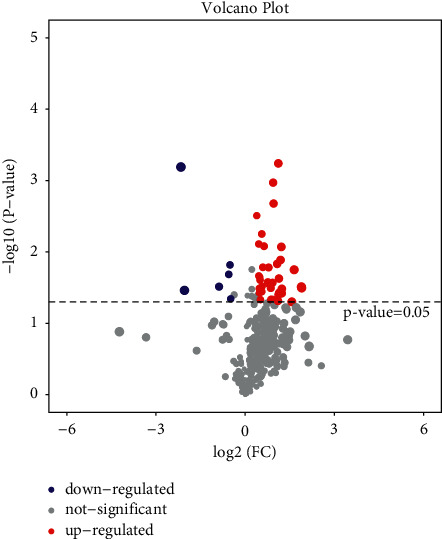
Male sample comparison.

After analyzing the differences in metabolite expression in the samples, the volcanic map was used to further analyze the significant differences in the samples. The closer the sample expression, the more similar the expression in the sample. The color level represents the abundance of sample expression. The more red it is, the more obviously upregulated it is. The more green it is, the more obviously downregulated it is. As can be seen from Figures 5–7, most samples in the whole sample and female sample were significantly upregulated but not so significantly upregulated in the male sample.

### 3.3. Differential Metabolite Analysis

**Figure 8:**
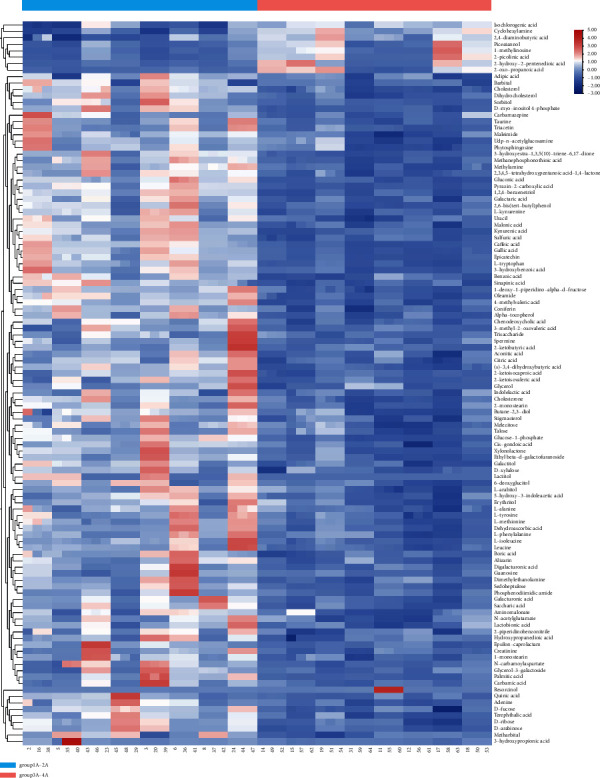
Whole-sample clustering heat map analysis.

The clustering results of 48 samples in four groups are shown (Table 2). As can be seen from Figure 8, in the whole sample, the content of metabolites such as cyclohexylamine chlorate, 2,4-diaminobutyric amygdalol, L-methylinosine, 2-picolinic acid, and 2-hydroxy-2-glutaric acid in the control group was significantly higher than that in the experimental group, while the content of other substances in the experimental group was significantly higher than that in the control group. For example, adipic acid, barbiturate, cholesterol, dihydrocholesterol, sorbitol, d-inositol 4-phosphate carbamazepine, taurine, etc., further showed that there were significant differences in metabolite content between the control group and the experimental group in the whole sample.

**Figure 9:**
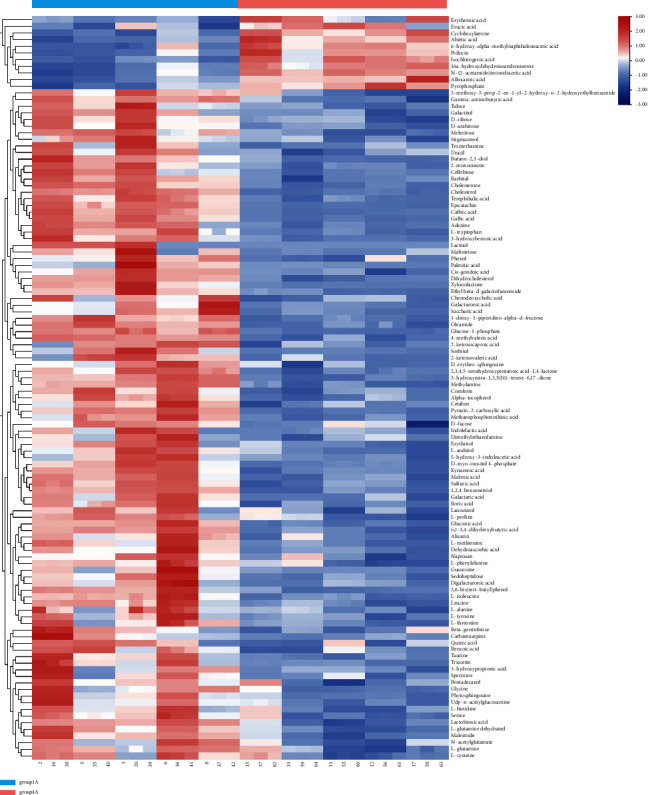
Female sample clustering heat map analysis.

In Figure 9 analysis shows that in the control group (women) red blood cells of folic acid, erucic acid, cyclohexylamine, fir acid, 6-hydroxy-alpha-methyl naphthalene acetic acid, chlorogenic acid, l6a-hydroxy dehydrogenation androsterone, N-(2-ethyl amide) iminodiacetic acid, four oxygen ethanoic acid, and pyrophosphate content of these materials was obviously higher than that of the experimental group (female). There are significant differences between the two.

**Figure 10:**
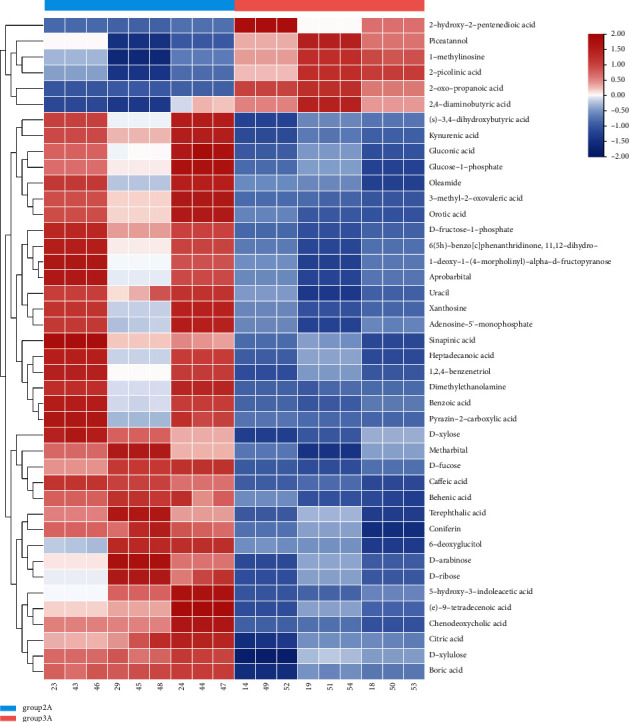
Male sample clustering heat map analysis.

According to Figure 10, the expression levels of 2-hydroxy-2-pentenedioic acid matrine, L-methylinosine, 2-pyridinic acid, 2-oxypropionic acid, and 2,4-diaminobutyric acid in the control group (male) were significantly higher than those in the experimental group (male), and the (S) -3 obtained in the experimental group (male) was significantly higher than that in the experimental group (male). The contents of 4-dihydroxybutyric acid, gluconic acid, glucose-L-phosphatidylurea, and 3-methyl-2-oxovalerate were significantly higher than those in the control group (male), and there were significant differences in metabolite expression between the two groups.

### 3.4. Metabolic Pathway Analysis

**Figure 11:**
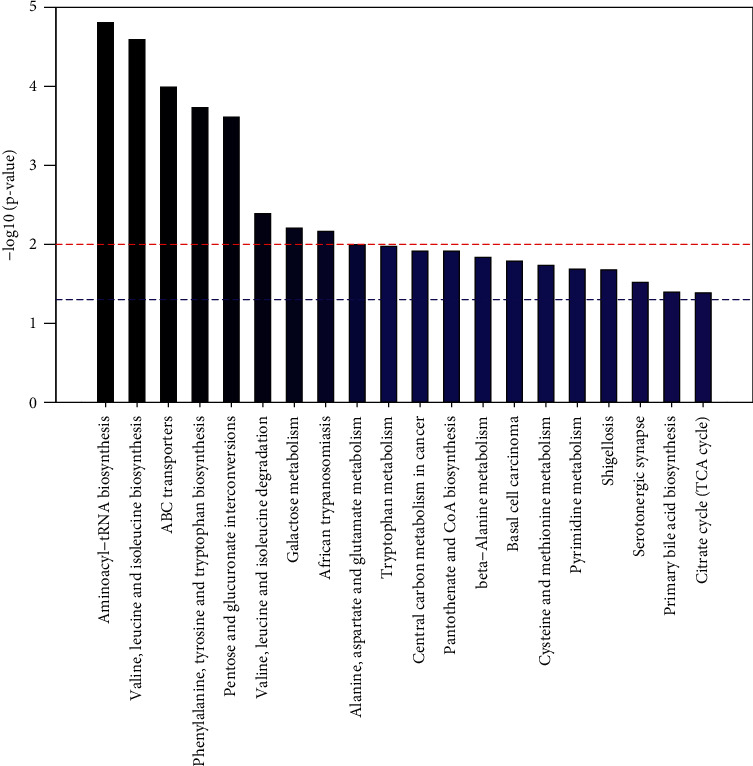
Enrichment of metabolic pathways in the whole sample (top 20).

**Figure 12:**
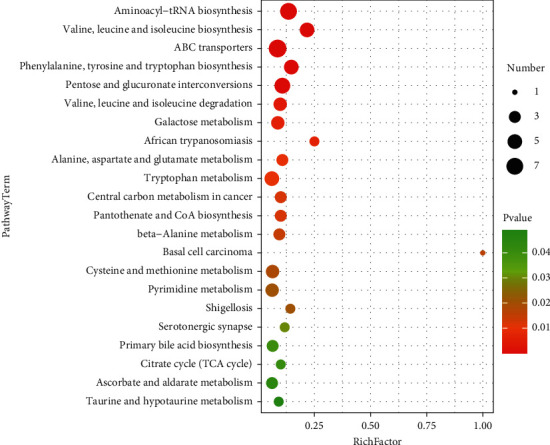
Metabolic bubble of the whole sample *P* < 0.05(*P* < 0.05).

The metabolic pathway enrichment map was used to sort all the metabolites, and it was found that the distribution of metabolites with a significance of less than 0.05 was not consistent in the whole sample, female sample, and male sample. As can be seen from the metabolic pathway enrichment diagram（Figure 11） and bubble diagram （Figure 12） of the whole sample below, metabolic pathways with high enrichment degree in the whole sample are as follows: ammonia acyl tRNA biosynthesis, valine, leucine and tryptophan biosynthesis, ABC transporters, phenylalanine, tyrosine and tryptophan biosynthesis, mutual transformation between the pentose and glucuronic acid, valine, leucine and isoleucine degradation and metabolism of galactose, African trypanosomiasis, alanine, glutamic acid and aspartic acid metabolism, tryptophan metabolism, central carbon metabolism, pantothenic acid and CoA biosynthesis, basal cell carcinoma, *β*-alanine metabolism, cysteine and methionine metabolism, pyrimidine metabolism, Shigellosis, serotonergic synapses, primary bile acid biosynthesis, tricarboxylic acid cycle, ascorbic acid and uronic acid metabolism, and taurine and low-taurine metabolism.

**Figure 13:**
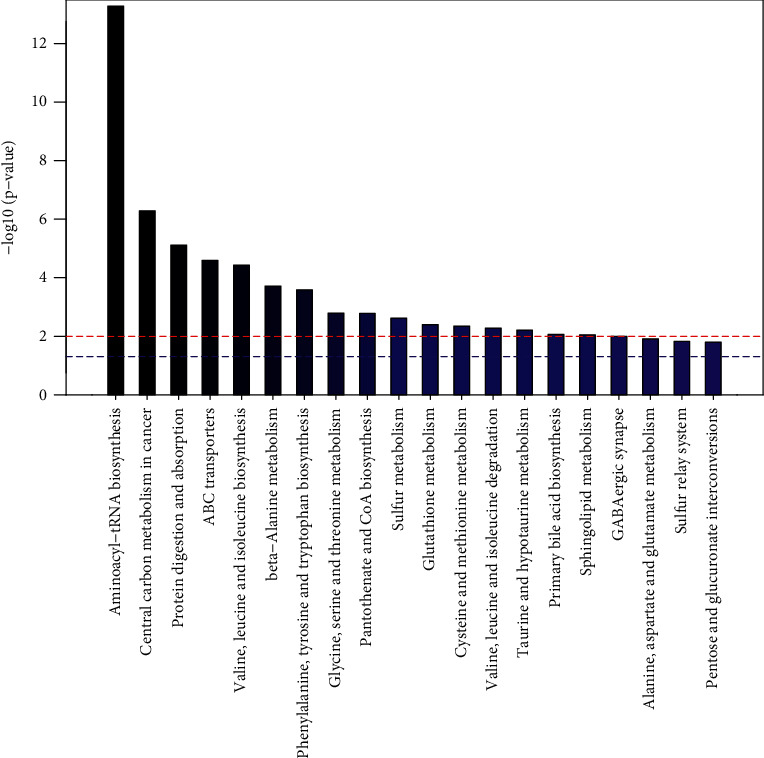
Accumulation of metabolic pathways in female samples (top 20).

**Figure 14:**
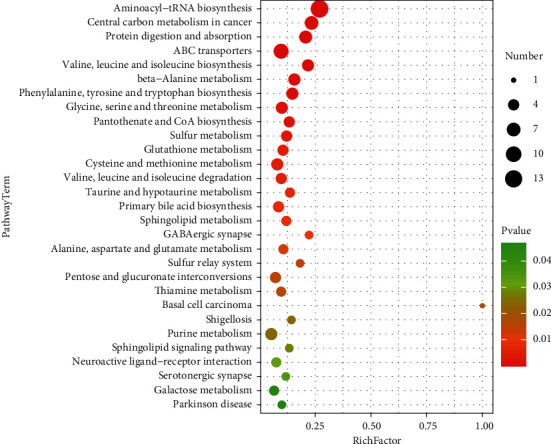
Metabolic bubble of female sample (*P* < 0.05).

As can be seen from the metabolic pathway enrichment diagram (Figure 13) and bubble diagram (Figure 14) of female samples, the metabolic pathways with higher enrichment degree in female samples are as follows: ammonia acyl tRNA biosynthesis, cancer center of carbon metabolism, protein digestion and absorption, ABC transporters, valine, leucine and isoleucine biosynthesis, beta alanine metabolism, phenylalanine, tyrosine and tryptophan biosynthesis, glycine, serine and threonine metabolism and biosynthesis of pantothenic acid and CoA, sulfur metabolism, GSH metabolism, cysteine and methionine metabolism, valine, leucine and isoleucine degradation, taurine and low metabolism of taurine, primary bile acid biosynthesis, sheath lipid metabolism, serotonin can synapses, alanine, glutamic acid, aspartic acid and metabolism of sulfur relay system, exchange of pentose and glucuronic acid, and thiamine metabolism. Among them, the enrichment degree of the first 16 metabolic pathways was obvious.

**Figure 15:**
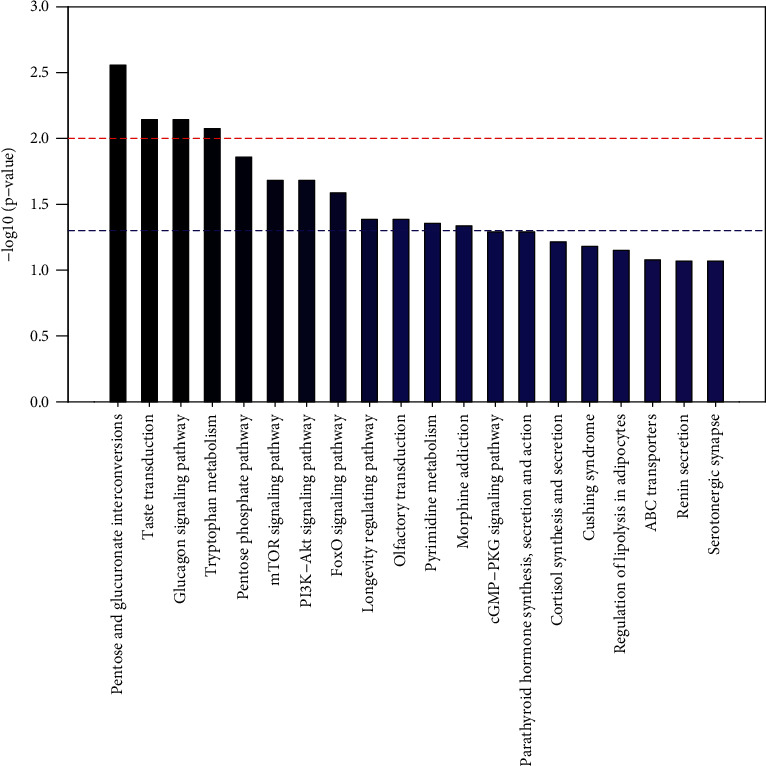
Accumulation of metabolic pathways in male samples (top 20).

**Figure 16:**
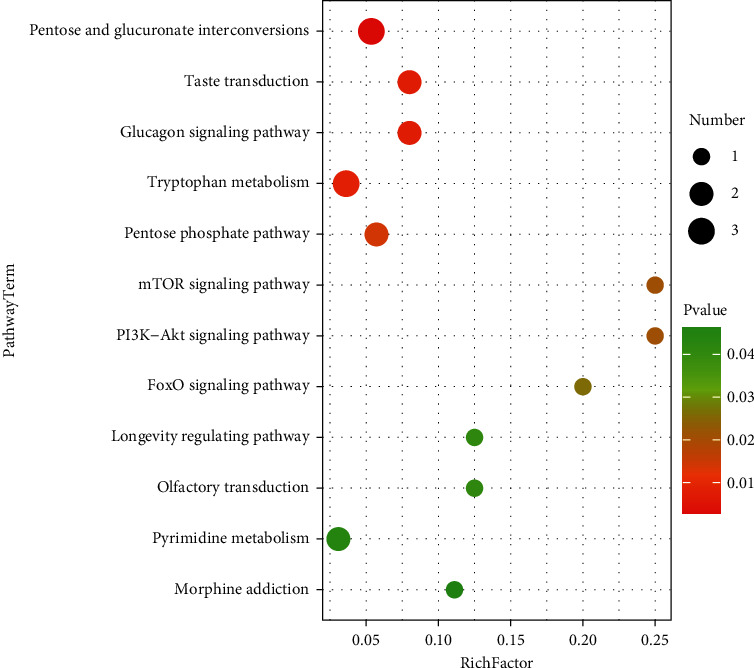
Metabolic bubble of male sample (*P* < 0.05).

As can be seen from the metabolic pathway enrichment diagram (Figure 15) and bubble diagram (Figure 16) of the male samples below, the metabolic pathways with enrichment degrees from high to low are as follows: mutual conversion of pentose and glucuronic acid, taste transduction, glucagon signaling pathway, tryptophan metabolism, pentose phosphate pathway, TOR signaling pathway, PL3K-Akt signaling pathway, FoxO signaling pathway, life regulation pathway, olfactory transduction, pyrimidine metabolism, and morphine addiction.

## 4. Discussion

Compared with the content in the experimental group, more metabolites found in the control group metabolites are mostly intermediate metabolites of various biochemical reactions, such as 2-hydroxy-2-glutaric acid is the pentose phosphate intermediate [8], chlorinated acid cyclohexylamine is cyclohexylamine-generated intermediate [9], and l-methylinosine is the center of the cancer carbon metabolites [7, 10–12]. If these products are not eliminated in time, there will be some harm to the human body. Compared with the control group, the metabolites of the experimental group are mostly final metabolites, and the metabolism of the experimental group is more thorough, so the metabolic burden on the body is less. Metabolic channels, in the control group and experimental group, show significant differences in metabolic channels with aminoacyltRNA biosynthesis, valine, leucine and tryptophan biosynthesis, ABC transporters, phenylalanine, tyrosine and tryptophan biosynthesis, mutual transformation between the pentose and glucuronic acid, valine, leucine and isoleucine degradation and metabolism of galactose, African trypanosomiasis (African trypanosomiasis), alanine, aspartic acid, and glutamate metabolism. In the biosynthesis of aminoacyltRNA, aminoacylation of amino acids, and tRNA can improve the accuracy of genetic information translation [13–15]. In the biosynthesis of valine, leucine, and tryptophan, leucine performs a large amount of aminotransferase in muscle to promote the synthesis of glutamine [16], while glutamine can maximize the synthesis of muscle protein [17], thus promoting the generation of muscle and making the human body stronger. The existence of ABC transporter can transport substrates including inorganic ions, monosaccharides, polysaccharides, cholesterol, phospholipids, amino acids, peptides, proteins, toxins, drugs, antibiotics, and heterologous substances [18], which can promote the occurrence of various metabolic reactions and reduce toxic reactions of the body at the same time. The biosynthesis of phenylalanine, tyrosine, and tryptophan is also a process of protein synthesis, which can serve for muscle growth. Mutual conversion of pentose and glucuronic acid mainly ensures the occurrence of the tricarboxylic acid cycle and pentose phosphate pathway and provides energy for the body. In addition to protein synthesis, protein degradation reaction was more frequent in the experimental group, indicating that the Andai therapy promoted the metabolism of the body. It also helps the body convert galactose to glucose, preventing African trypanosomiasis.

Andai therapy has different metabolic effects in men and women. The results of female metabolites and metabolic pathways showed the following conclusions. The human body commonly used plasma folic acid, folic acid level measured serum folate, and red blood cells, red blood cell folate levels are long-term changes in state and folic acid reserve of the folic acid in the body [19], and the control group (women) red blood cells of folic acid content are higher than the experimental group. Thus, they have a certain impact on the organism. Pyrophosphate is a synthetic material of the pentose phosphate pathway, and because the control group (women) did not receive the Andai treatment compared to the experimental group (women), levels of these substances remain high. The control group (women) had insufficient cyclohexylamine metabolism, resulting in high cyclohexylamine levels, because of the lack of Andai therapy. Cyclohexylamine can cause central anesthesia, optic neuroretinopathy, metabolic alkalosis, etc. [20], and also has certain harm to the environment. In addition to this metabolite, abietic acid, 6-hydroxy-*α*-methylnaphthalene acetic acid, isochlorogenic acid, L6A-hydroxy-dehydroandrosterone, N- (2-acetamyl) iminodiacetic acid, tetraoxy acetic acid, etc., were also higher in the control group (female). According to these results, Andai therapy may reduce folic acid content in women but can also promote the occurrence of cyclohexylamine metabolism and pentose phosphate pathway, so as to reduce the body's awaiting metabolites and promote the body's energy metabolism and basic metabolism. Compared with the control group (women), the energy metabolism of the experimental group (women) was more active, protein synthesis and metabolism were more active, but different from the whole sample, and female cancer is more typical in central carbon metabolism, *β*-alanine metabolism, pantothenic acid and coenzyme A biosynthesis, sulfur metabolism, glutathione metabolism, taurine and low-taurine metabolism, primary bile acid biosynthesis, sphingolipid metabolism, serotonergic synapses, and so on. For example, the central carbon metabolism of cancer is more active, in which aerobic glycolysis, glutamine decomposition, one-carbon metabolism, pentose phosphate pathway, fatty acid de novo synthesis, etc., can be smoothly carried out, reducing the occurrence of metabolic disorders, thus indicating that Andai therapy may play a certain role in tumor prevention. *β*-alanine metabolism is not involved in protein or enzyme synthesis, it is mainly produced by the degradation of dihydrouracil and carnosine, as an important component of the spontaneously produced peptide carnosine and vitamin B5, and it is eventually metabolized into acetic acid under normal conditions, which is an important substance in the tricarboxylic acid cycle; therefore, Andai therapy promotes *β*-alanine metabolism and the body's energy metabolism. Pantothenic acid and coenzyme A biosynthesis are mainly involved in the metabolism of fatty acids and pyruvate, indicating that Andai therapy can promote the decomposition of fat, reduce the accumulation of ketones in the body, and reduce the toxic effect on the body. Sulfur metabolism is also a way to help the body reduce sulfur toxic substances; Glutathione metabolism can provide NADPH for pentose phosphate by metabolism to promote energy metabolism and achieve the role of calorie burning. 5-hydroxytryptamine can be catalyzed by monoamine oxidase into 5-hydroxytryptamine aldehyde and 5-hydroxyindoleacetic acid and excreted in urine, and this reaction indicates that Andai therapy can be beneficial to vascular contraction and smooth muscle function. Primary bile acid biosynthesis is associated with fat burning, sheath fat metabolism of ceramide can regulate cell differentiation and growth, and as a highly effective skin moisturizer, Andai therapy can promote the scabbard lipid metabolism, to produce these kinds of beneficial substances, explain Andai therapy, can promote female cell growth and development, and maintain the state of the skin effect.

The results of male metabolites and metabolic pathways showed the following conclusions. Compared with the experimental group (male), the content of L-methylinosine in the body of the control group (male) was significantly higher, which is a synthetic material of adenosine, which is the material of ATP energy reaction in the body. It can be seen that the energy metabolism of the experimental group (male) was more active than that of the control group (male). The 2-pyridinic acid (2-pyridinic acid) is a harmful substance, which may cause toxic reactions in the body and is a side reaction product of incomplete completion of the tricarboxylic acid cycle. This metabolite was higher in the control group (male), which further indicates that the Andai therapy can promote the thorough metabolism of the body and reduce the formation of toxic substances. Two groups of metabolite concentrations with significantly higher metabolic pathways have a pentose and reciprocal transformation of glucuronic acid, taste the conduction, glucagon, signaling pathways, and tryptophan metabolism, and the pentose phosphate pathway shows that compared with the control group (men), the experimental group (male), and the way these pathways are more active, and it is further illustrated that Andai therapy can make men taste more sensitive. Energy metabolism is more active, promoting the occurrence of the glucagon signaling pathway, enabling glucagon to regulate insulin secretion of pancreatic *β* cells through the cAMP signaling pathway, and reducing the incidence of diabetes. Tryptophan can be transformed into 5-ht after oxidation and de-enucleation, which mainly exists in brain tissue and gastrointestinal wall, but rarely exists in blood [21]. The 5-HT produced by tryptophan can cause microvasoconstriction and increase blood pressure, and also acts as a neurotransmitter. Tryptophan can also be converted to niacin, which is a precursor to the synthesis of NAD and NADP, coenzymes of oxygen-free dehydrogenases involved in redox reactions in vivo. It can be seen that although Andai therapy may lead to increased blood pressure in men, it also promotes energy metabolism in the body. Overall, Andai therapy can help men improve their appetite, boost their energy metabolism, and reduce the risk of diabetes, but it can also lead to high blood pressure.

## 5. Conclusions

Mongolian medical Andai therapy has a significant effect on metabolism in healthy people. It can promote the thorough metabolism of airframe, reduce the formation of toxic material, help airframe to transform galactose into glucose, promote the synthesis of muscle protein, and thereby promote the generation of muscle. Andai therapy has different effects on metabolism in healthy men and healthy women.

## Figures and Tables

**Table 1:** Basic sample information.

Group		Number of people	Sample no.
Experimental group (receiving Andai therapy)	Group 1 (female group)	15	2, 3, 5, 6, 8, 16, 20, 35, 36, 37, 38, 39, 40, 41, 42
	Group 2 (male group)	9	23, 24, 29, 43, 44, 45, 46, 47, 48

Control group (did not receive Andai therapy)	Group 3 (male group)	9	14, 18, 19, 49, 50, 51, 52, 53, 54
	Group 4 (female group)	15	11, 15, 17, 17, 31, 55, 56, 57, 58, 59, 60, 61, 62, 63, 64

**Table 2:** The number of differential metabolites in each comparison group.

Comparison group	Number of differential metabolites
Group 1/group 4	119
Group 2/group 3	48
Group 1-group 2/group 3-group 4	114

## Data Availability

The raw data required to reproduce these findings cannot be shared at this time as the data also form part of an ongoing study.

## Supplementary Materials

Supplementary Materials Supplement 1 is evidence for the principal component analysis (PCA) diagram. Supplement 2A is evidence for (group 1-group 4) female sample comparison volcanic map analysis representing metabolites, as shown in Figure 6. Supplement 2B is evidence for (group 2-group 3) male sample comparison volcanic map analysis representing metabolites, as shown in Figure 7. Supplement2C is evidence for (group 1-group 2 and group 3-group 4) full-sample comparison volcanic map analysis representing metabolites, as shown in Figure 5. Supplement 3A is evidence for (group 1A and group 4A) female sample clustering heat map analysis, as shown in Figure 9. Supplement 3B is evidence for (group 2A and group 3A) male sample clustering heat map analysis, as shown in Figure 10. Supplement 3C is evidence for (group 1A-2A and group 3A-4A) whole-sample clustering heat map analysis， as shown in Figure 8. Supplement 4A is evidence for (group 1A and group 4A) accumulation of metabolic pathways in female samples—Top20, as shown in Figure 13. Supplement 4B is evidence for (group 2 and group 3) accumulation of metabolic pathways in male samples—Top20, as shown in Figure 15. Supplement 4C is evidence for (group 1-2 and group 3-4) enrichment of metabolic pathways in the whole sample—Top20, as shown in Figure 11. Supplement 5A is evidence for (group 1 and group 4) metabolic bubble of female sample, as shown in Figure 14. Supplement 5B is evidence for (group 2 and group 3) metabolic bubble of male sample, as shown in Figure 16. Supplement 5C is evidence for metabolic pathways of (group 1-2 and group 3-4) metabolic bubble of the whole sample, as shown in Figure 12.
