# Supplementary material for: Study on the Metabonomics Mechanism of Mongolian Medical Andai Therapy on Healthy People
Source: Evid Based Complement Alternat Med. 2022 Jun 20;2022:1364408. doi: 10.1155/2022/1364408 (PMC9236767; doi:10.1155/2022/1364408)
Supplement: Supplementary Materials — Supplement 1 is evidence for the principal component analysis (PCA) diagram. Supplement 2A is evidence for (group 1-group 4) female sample comparison volcanic map analysis representing metabolites, as shown in Figure 6. Supplement 2B is evidence for (group 2-group 3) male sample comparison volcanic map analysis representing metabolites, as shown in Figure 7. Supplement2C is evidence for (group 1-group 2 and group 3-group 4) full-sample comparison volcanic map analysis representing metabolites, as shown in Figure 5. Supplement 3A is evidence for (group 1A and group 4A) female sample clustering heat map analysis, as shown in Figure 9. Supplement 3B is evidence for (group 2A and group 3A) male sample clustering heat map analysis, as shown in Figure 10. Supplement 3C is evidence for (group 1A-2A and group 3A-4A) whole-sample clustering heat map analysis， as shown in Figure 8. Supplement 4A is evidence for (group 1A and group 4A) accumulation of metabolic pathways in female samples—Top20, as shown in Figure 13. Supplement 4B is evidence for (group 2 and group 3) accumulation of metabolic pathways in male samples—Top20, as shown in Figure 15. Supplement 4C is evidence for (group 1-2 and group 3-4) enrichment of metabolic pathways in the whole sample—Top20, as shown in Figure 11. Supplement 5A is evidence for (group 1 and group 4) metabolic bubble of female sample, as shown in Figure 14. Supplement 5B is evidence for (group 2 and group 3) metabolic bubble of male sample, as shown in Figure 16. Supplement 5C is evidence for metabolic pathways of (group 1-2 and group 3-4) metabolic bubble of the whole sample, as shown in Figure 12. [file 1364408.f1.zip › 1364408.f1/Supplement 4A.pdf]

Supplement 4

Metabolite name	log2(FC)	KEGG	RED/BLUE
1,2,4-benzenetriol	0.680234037	C02814	red
16a-hydroxydehydroisoandrosterone	-0.802044386	C05139	blue
2-ketoisocaproic acid	0.617341466	C00233	red
2-ketoisovaleric acid	0.777428611	C00141	red
3-hydroxybenzoic acid	1.145573766	C00587	red
3-hydroxypropionic acid	0.662593189	C01013	red
5-hydroxy-3-indoleacetic acid	0.832564524	C05635	red
Abietic acid	-0.519091515	C06087	blue
Adenine	1.105514846	C00147	red
Alpha-tocopherol	1.080487079	C02477	red
Benzoic acid	0.728298546	C00539	red
Butane-2,3-diol	1.289169147	C00265	red
Caffeic acid	2.175288656	C01481	red
Carbamazepine	1.056557177	C06868	red
Cellobiose	0.542329743	C06422	red
Chenodeoxycholic acid	1.198159627	C02528	red
Cholesterol	0.850867485	C00187	red
Cholesterone	1.244338947	C00599	red
Cis-gondoic acid	1.250958778	C16526	red
Coniferin	1.404904958	C00761	red
Cyclohexylamine	-0.586295116	C00571	blue
D-erythro-sphingosine	0.548544974	C00319	red
D-fucose	0.869850354	C01018	red
D-myo-inositol 4-phosphate	1.048860735	C03546	red
D-ribose	1.258467135	C00121	red
Dehydroascorbic acid	0.762348682	C05422	red
Digalacturonic acid	1.250291725	C02273	red
Dimethylethanolamine	0.652225878	C04308	red
Epicatechin	1.413767767	C09727	red
Erucic acid	-0.616512091	C08316	blue

Erythritol	0.812062446	C00503	red
Galactaric acid	0.92780538	C00879	red
Galactitol	0.930166948	C01697	red
Galacturonic acid	1.541683064	C08348	red
Gallic acid	1.746939015	C01424	red
Gamma-aminobutyric acid	0.501842054	C00334	red
Gluconic acid	1.186030887	C00257	red
Glucose-1-phosphate	1.235651097	C00103	red
Glycine	1.04883952	C00037	red
Guanosine	1.216131251	C00387	red
Indolelactic acid	0.766331284	C02043	red
Kynurenic acid	0.860545974	C01717	red
L-alanine	0.681471266	C00041	red
L-arabitol	1.075169908	C00532	red
L-cysteine	0.673296562	C00097	red
L-glutamine	0.661330053	C00064	red
L-histidine	0.863854263	C00135	red
L-isoleucine	0.775678032	C00407	red
L-methionine	1.039892638	C00073	red
L-phenylalanine	0.670149164	C00079	red
L-proline	0.64564067	C00148	red
L-threonine	0.636237913	C00188	red
L-tryptophan	1.491307925	C00078	red
L-tyrosine	0.876376593	C00082	red
Lanosterol	0.980563782	C01724	red
Leucine	0.722073928	C00123	red
Malonic acid	0.595472659	C04025	red
Maltotriose	2.457503577	C01835	red
Melezitose	1.355097042	C08243	red
Methylamine	0.539378522	C00218	red
N-acetylglutamate	0.830931499	C00624	red
Naproxen	0.909659757	C01517	red
Oleamide	0.67370783	C19670	red

Palmitic acid	2.337305505	C00249	red
Phenol	0.63817271	C15584	red
Phytosphingosine	0.703450727	C12144	red
Psilocin	-0.513620039	C08312	blue
Pyrazin-2-carboxylic acid	1.393197531	C19915	red
Pyrophosphate	-0.780515525	C00013	blue
Quinic acid	0.847754145	C00296	red
Saccharic acid	1.298433444	C00818	red
Sedoheptulose	1.350997293	C08355	red
Serine	0.737922762	C00065	red
Sorbitol	3.137025981	C00794	red
Spermine	0.635623682	C00750	red
Stigmasterol	0.757966602	C05442	red
Sulfuric acid	0.72044524	C00059	red
Taurine	1.25772005	C00245	red
Terephthalic acid	0.563246996	C06337	red
Tromethamine	0.702443786	C07182	red
Udp-n-acetylglucosamine	0.711956678	C00043	red
Uracil	0.600324895	C00106	red
Xylonolactone	1.257324063	C02266	red

Supplement 4A:We analysis representing metabolic pathways of Group1 and Group4. Metabolic pathway analysis was performed on control and experimental female samples. KEGG(https://www.kegg.jp/) database was used to analyze metabolic pathway enrichment of differential metabolites. Enrichment factor (Rich factor= number of significantly differentiated metabolites/total metabolites in this pathway), the higher the Rich factor, the greater the degree of enrichment; The color from red to green indicates that p-values decrease successively. The larger the point is, the more metabolites are enriched into this pathway.
