# Supplementary material for: Study on the Metabonomics Mechanism of Mongolian Medical Andai Therapy on Healthy People
Source: Evid Based Complement Alternat Med. 2022 Jun 20;2022:1364408. doi: 10.1155/2022/1364408 (PMC9236767; doi:10.1155/2022/1364408)
Supplement: Supplementary Materials — Supplement 1 is evidence for the principal component analysis (PCA) diagram. Supplement 2A is evidence for (group 1-group 4) female sample comparison volcanic map analysis representing metabolites, as shown in Figure 6. Supplement 2B is evidence for (group 2-group 3) male sample comparison volcanic map analysis representing metabolites, as shown in Figure 7. Supplement2C is evidence for (group 1-group 2 and group 3-group 4) full-sample comparison volcanic map analysis representing metabolites, as shown in Figure 5. Supplement 3A is evidence for (group 1A and group 4A) female sample clustering heat map analysis, as shown in Figure 9. Supplement 3B is evidence for (group 2A and group 3A) male sample clustering heat map analysis, as shown in Figure 10. Supplement 3C is evidence for (group 1A-2A and group 3A-4A) whole-sample clustering heat map analysis， as shown in Figure 8. Supplement 4A is evidence for (group 1A and group 4A) accumulation of metabolic pathways in female samples—Top20, as shown in Figure 13. Supplement 4B is evidence for (group 2 and group 3) accumulation of metabolic pathways in male samples—Top20, as shown in Figure 15. Supplement 4C is evidence for (group 1-2 and group 3-4) enrichment of metabolic pathways in the whole sample—Top20, as shown in Figure 11. Supplement 5A is evidence for (group 1 and group 4) metabolic bubble of female sample, as shown in Figure 14. Supplement 5B is evidence for (group 2 and group 3) metabolic bubble of male sample, as shown in Figure 16. Supplement 5C is evidence for metabolic pathways of (group 1-2 and group 3-4) metabolic bubble of the whole sample, as shown in Figure 12. [file 1364408.f1.zip › 1364408.f1/Supplement 4B.pdf]

Supplement 4B

Metabolite name	log2(FC)	KEGG	RED/BLUE
1,2,4-benzenetriol	0.489716498	C02814	red
2-picolinic acid	-0.509865137	C10164	blue
3-methyl-2-oxovaleric acid	1.190247445	C03465	red
5-hydroxy-3-indoleacetic acid	0.474074744	C05635	red
Adenosine-5'-monophosphate	1.105911538	C00020	red
Aprobarbital	1.890699901	C07826	red
Behenic acid	0.941643503	C08281	red
Benzoic acid	0.920252338	C00539	red
Caffeic acid	1.114459841	C01481	red
Chenodeoxycholic acid	1.216532942	C02528	red
Citric acid	0.566736514	C00158	red
Coniferin	0.456488518	C00761	red
D-fructose-1-phosphate	0.389930589	C01094	red
D-fucose	0.956021225	C01018	red
D-ribose	1.12528852	C00121	red
D-xylose	0.788226999	C00181	red
D-xylulose	0.765814103	C00310	red
Dimethylethanolamine	0.459181587	C04308	red
Gluconic acid	0.861682995	C00257	red
Glucose-1-phosphate	0.559551137	C00103	red
Kynurenic acid	0.635635056	C01717	red
Oleamide	0.514002917	C19670	red
Orotic acid	1.076051989	C00295	red
Piceatannol	-0.48728171	C05901	blue
Pyrazin-2-carboxylic acid	0.958895979	C19915	red
Sinapinic acid	0.482218982	C00482	red
Terephthalic acid	0.502244315	C06337	red
Uracil	0.594520976	C00106	red
Xanthosine	1.233970806	C01762	red

Supplement 4B:This annex is the enrichment map analysis evidence representing the metabolic pathways of Group2 and Group3. Metabolic pathway analysis was performed on male samples from the control group and the experimental group. KEGG(https://www.kegg.jp/) database was used to analyze metabolic pathway enrichment of differential metabolites. Enrichment factor (Rich factor= number of significantly differentiated metabolites/total metabolites in this pathway), the higher the Rich factor, the greater the degree of enrichment; The color from red to green indicates that p-values decrease successively. The larger the point is, the more metabolites are enriched into this pathway.
