# Supplementary material for: Study on the Metabonomics Mechanism of Mongolian Medical Andai Therapy on Healthy People
Source: Evid Based Complement Alternat Med. 2022 Jun 20;2022:1364408. doi: 10.1155/2022/1364408 (PMC9236767; doi:10.1155/2022/1364408)
Supplement: Supplementary Materials — Supplement 1 is evidence for the principal component analysis (PCA) diagram. Supplement 2A is evidence for (group 1-group 4) female sample comparison volcanic map analysis representing metabolites, as shown in Figure 6. Supplement 2B is evidence for (group 2-group 3) male sample comparison volcanic map analysis representing metabolites, as shown in Figure 7. Supplement2C is evidence for (group 1-group 2 and group 3-group 4) full-sample comparison volcanic map analysis representing metabolites, as shown in Figure 5. Supplement 3A is evidence for (group 1A and group 4A) female sample clustering heat map analysis, as shown in Figure 9. Supplement 3B is evidence for (group 2A and group 3A) male sample clustering heat map analysis, as shown in Figure 10. Supplement 3C is evidence for (group 1A-2A and group 3A-4A) whole-sample clustering heat map analysis， as shown in Figure 8. Supplement 4A is evidence for (group 1A and group 4A) accumulation of metabolic pathways in female samples—Top20, as shown in Figure 13. Supplement 4B is evidence for (group 2 and group 3) accumulation of metabolic pathways in male samples—Top20, as shown in Figure 15. Supplement 4C is evidence for (group 1-2 and group 3-4) enrichment of metabolic pathways in the whole sample—Top20, as shown in Figure 11. Supplement 5A is evidence for (group 1 and group 4) metabolic bubble of female sample, as shown in Figure 14. Supplement 5B is evidence for (group 2 and group 3) metabolic bubble of male sample, as shown in Figure 16. Supplement 5C is evidence for metabolic pathways of (group 1-2 and group 3-4) metabolic bubble of the whole sample, as shown in Figure 12. [file 1364408.f1.zip › 1364408.f1/Supplement 2 A.pdf]

Supplement 2A

Metabolite name	P-value	log2(FC)	VIP
Ethylamine	0.074116892	0.159567755	0.46718433
Methylamine	5.88489E-05	0.48149831	1.062336762
Tranexamic acid	0.598154345	0.114986078	0.207601914
Diethylamine	0.345820579	0.075883933	0.246175963
Boric acid	1.46279E-05	0.791889432	1.375684618
Digitoxose	1.43232E-05	0.172191964	0.644802568
Ethylene glycol	5.61344E-06	0.073187156	0.416267845
Malonic acid	3.84776E-05	0.546149549	1.136539023
Nicotinamide	5.57985E-05	0.305110071	0.844556479
Piperidone	0.000207274	0.295806097	0.818648893
Ferulic acid	5.78386E-05	0.328597753	0.875095333
Dimethylethanolamine	0.004017499	0.743239234	1.20037269
2-picolinic acid	0.042009392	-0.452880991	0.770710703
Butane-2,3-diol	1.37923E-06	1.43927414	1.939366458
Norvaline	0.399479015	-0.09765827	0.215376634
3-pyridinol	0.001726273	0.312781837	0.791645127
Kynurenic acid	9.81871E-06	0.787390323	1.377826058
Pyruvic acid	0.350189363	-0.199525041	0.326756534
Beta-alanine	0.013448851	0.812836494	1.21629492
Diethylcarbamic acid	0.000257813	0.359597928	0.90004685
Phenol	0.003754767	0.565784036	1.034899959
L-lactic acid	0.811914986	0.030189394	0.06016637
Maleic acid	0.318258267	0.018584787	0.126283407
Carbamic acid	0.064319808	0.707387691	0.96907236
Cyclohexylamine	2.37384E-06	-0.574527578	1.196602017
2-ketoadipic acid	0.001082704	0.382368858	0.898814291
4-methylvaleric acid	7.73154E-06	0.461285661	1.048652636
Glycolic acid	0.237326078	0.154488543	0.37736448
Sulfuric acid	1.40642E-05	0.711423421	1.312059697
Cytosin	0.000588933	0.453685538	0.956117603

2-oxo-propanoic acid	0.166313631	-1.134546198	0.567671952
Resorcinol	0.001797674	0.457087179	0.939862297
Maleimide	5.82057E-06	0.727220654	1.343597551
2-ketobutyric acid	0.007546699	0.404164924	0.823758722
L-alanine	0.04608993	0.444627228	0.771246116
2-ketoisovaleric acid	0.003239545	0.748601632	1.165838564
4-aminophenol	0.717399988	0.07438774	0.204044053
2-hydroxybutyric acid	0.213591466	0.254415411	0.455637742
Oxalic acid	0.555415219	0.09040035	0.219033811
Propane-1,3-diol	0.862863728	0.021078554	0.119640262
3-hydroxypropionic acid	0.01056962	0.907986867	1.267556878
P-cresol	0.079356891	1.185744566	0.743313686
(r)-3-hydroxybutyric acid	0.999996696	-2.10621E-06	0.264204584
2-hydroxy-3-methylbutyric acid	0.119708707	0.476511519	0.720927222
3-hydroxybutyric acid	0.401730941	-0.017309709	0.111267858
Alizarin	0.000845367	0.811128551	1.308785108
Urocanic acid	0.824870234	-0.060982241	0.299200258
2-aminoisobutyric acid	0.384725821	0.171893314	0.355050717
Methanephosphonothioic acid	5.45799E-06	0.859970703	1.439241173
4-hydroxycinnamic acid	4.19455E-05	0.093243283	0.47191439
N-methylalanine	0.413640494	-0.099594693	0.22216547
N-acetylglutamate	0.009312674	0.727175497	1.203885078
3-methyl-2-oxovaleric acid	0.018160966	0.516076566	0.896893314
Succinic acid	0.012447932	0.447196349	0.866225071
Epsilon-caprolactam	8.6884E-05	0.500274312	1.062661957
2-methylglyceric acid	0.002523589	0.479524013	0.965676544
2-hydroxyvaleric acid	0.000838781	0.347601255	0.857135689
Thymidine	0.958840304	0.006777733	0.013420965
3-hydroxyisovaleric acid	0.357475545	0.13789104	0.288872122
Gamma-aminobutyric acid	0.000201506	0.437951411	0.98831198
2-ketoisocaproic acid	0.000454652	0.806231942	1.308078454
L-valine	0.013926337	0.337790553	0.72797569
Hypoxanthine	0.940407299	-0.014521247	0.023706257

Cyano-l-alanine	0.309568982	0.086675057	0.254335979
Adipic acid	0.051697778	0.778693318	0.818724025
L-alanine-alanine	0.001108636	0.251051575	0.72750996
Dopamine	0.022707094	0.19450134	0.564712905
2-piperidinobenzonitrile	0.206475047	0.492144002	0.874754433
-hydroxy-3-methylpentanoic ac	2.00107E-05	0.376367059	0.947719259
Urea	0.001431294	0.210749614	0.661656491
Amine	0.008034592	0.819018309	1.22752221
Benzoic acid	0.000467382	0.684167458	1.220339753
Sinapinic acid	3.27729E-05	0.41838288	0.999559324
N-methylglutamic acid	0.864627762	-0.081077662	0.222139187
1-methylhydantoin	0.288848388	3.904002282	1.650139027
Ethanolamine	0.087752242	0.047393786	0.234998559
Leucine	0.001119578	0.627285789	1.116622404
Ciliatine	0.005135377	0.313579109	0.771983828
Glycerol	0.005219339	0.562109482	1.021001662
Pyridoxine	0.904096522	-0.024715479	0.045960418
Citraconic acid	0.536158002	-0.102716275	0.173197146
odecanoic acid, isopropanol est	0.000847919	0.237453763	0.714241784
Pyrazin-2-carboxylic acid	2.97447E-09	1.359679831	1.894975125
3,6-anhydro-d-galactose	0.064282783	-0.395341422	0.699833456
L-isoleucine	0.000279318	0.687970361	1.215461538
L-proline	0.327954427	0.293415016	0.59000719
Glycine	0.023115651	0.77401183	1.11604222
Hydroquinone	0.470986087	-0.109840302	0.269217556
Xanthine	0.892233354	0.024966211	0.066317656
Allantoin	0.181619113	0.487442624	0.673633477
Pyrrole-2-carboxylic acid	0.085782296	0.177464922	0.465895288
Butanedioic acid	0.354160162	0.127774101	0.305252842
Metharbital	0.011361509	0.841828654	1.105646642
Oleamide	3.54467E-05	0.610477202	1.209967548
Glucosamine	0.510866986	-0.162918622	0.197129414
Phytol	0.006919362	0.263575076	0.693707319

Glyceric acid	0.256285338	0.431770822	0.533096526
Oxoproline	0.674796425	0.392995645	0.219978387
Uracil	0.001333297	0.824540818	1.339125698
L-threonic acid	0.015505716	-0.827911797	1.132759743
1,2,4-benzenetriol	3.77047E-06	0.650879089	1.267344075
4-deoxyerythronic acid	0.02123563	0.784225184	1.191341755
Fumaric acid	0.124678845	-0.268561647	0.529290477
Pinitol	0.278636979	0.288998375	0.639599272
Formononetin	0.034949282	0.506300818	0.875057299
Erythrose	0.292785671	-0.169962422	0.38368278
Serine	0.003210732	0.605444819	1.132033758
Dihydroxymalonic acid	0.039988217	1.064201078	1.330035424
Pelargonic acid	0.020339457	0.26776156	0.681619414
D-erythro-sphingosine	0.007892593	0.544130739	1.013729817
Phytosphingosine	0.002580693	0.580395961	1.104789885
Udp-n-acetylglucosamine	0.00504239	0.56169371	1.063578998
Shikimic acid	0.32149935	0.675459524	0.100487106
O-phosphoserine	0.666937152	0.123523135	0.013823351
L-threonine	0.064459799	0.377545022	0.742843711
Hydroxypropanedioic acid	0.151751115	0.464321623	0.85543393
Abietic acid	0.000352652	-0.528350483	1.070203863
2,3-dihydroxy-acrylic acid	0.080138343	0.609461752	0.816471255
N-carbamoylaspartate	0.019821956	1.46438066	1.357518621
Glutaric acid	0.077869398	0.320629464	0.644574398
Glycyl proline	0.329318512	0.486946437	0.157095714
Putrescine	1.74497E-05	0.368735124	0.94378484
1,2,4-butanetriol	0.364102513	-0.239854049	0.680782617
(s)-3,4-dihydroxybutyric acid	0.001863247	0.956242814	1.416904701
D-fructose-1-phosphate	0.106918633	0.260548502	0.544286482
N-methyl-l-leucine	0.185807058	1.708804791	1.639186708
2,4-diaminobutyric acid	0.026387364	-0.665299	1.09624959
9-decenoic acid	0.27060566	0.278283193	0.420419124
Phosphenodiimidic amide	0.011133261	1.156408951	1.45844133

Cytidine-5-monophosphate	0.396857245	0.374516603	0.361065252
Capric acid	0.001119571	0.312247141	0.800917631
Adrenaline	0.312738669	-2.371763234	0.570568605
Aminomalonate	0.020304034	0.915332969	1.249273916
L-glutamine dehydrated	0.000245249	0.725606895	1.289962377
1,3-dihydroxypyridine	0.024448515	-0.242458672	0.583485007
L-cysteine-glycine	0.010364182	0.392152559	0.817995538
Biphenyl	0.460823825	0.182383219	0.566807424
Noradrenaline	0.391712736	-0.332051242	0.351093347
Malic acid	0.726826887	0.065575963	0.143742495
N-acetylornithine	0.131126019	-0.20836109	0.451788037
Tetracosane	0.177883103	0.015768578	0.13273195
Scopoletin	0.000710617	0.300311679	0.799704033
Galactitol	0.006476463	0.979361087	1.384582337
N-acetylputrescine	0.174924543	-0.16196219	0.374048082
Erythritol	0.008193284	0.647631457	1.098769508
Tromethamine	0.003548214	0.744500065	1.199640047
L-methionine	0.000465944	0.855785231	1.361811356
Dehydroascorbic acid	0.000598272	0.674660335	1.201234387
L-aspartic acid	0.04312742	0.429521958	0.777030453
4-hydroxyproline	0.561690908	-0.204280369	0.364157112
Pentadecanol	0.002228864	0.609324001	1.141130426
2,6-bis(tert-butyl)phenol	0.000493282	0.480373159	1.024378031
Aminoadipic acid	0.664928724	0.235753961	0.060453457
Creatinine	0.094944089	0.472077694	0.779247829
L-cysteine	0.030216454	0.472426523	0.882079561
Glucosaminic acid	0.046589264	-0.481792942	0.966187368
Erythronic acid	0.023860486	-0.659076354	0.964813187
4-acetamino phenol	0.138678964	0.864391107	1.003471863
Naproxen	0.01272362	0.746751255	1.19544788
L-2-hydroxyglutaric acid	0.726430178	0.095331528	0.267553895
Oxoglutaric acid	0.145411416	0.446741355	0.867502235
5-hydroxy-3-indoleacetic acid	0.003309441	0.734049998	1.18459765

Nicotianamine	0.551554693	0.271822105	0.530831392
Dithiothreitol	0.042373741	0.293095369	0.642079511
Allantoic acid	0.156213694	0.28797219	0.572026565
N-acetyl-l-aspartic acid	0.357756827	0.50649512	0.127425255
Cyanuric acid	0.15777842	3.678694178	2.133748662
Glucose-1-phosphate	1.06763E-08	1.140630103	1.738774521
Xylonolactone	0.341597154	0.493781831	1.12468362
L-glutamic acid	0.335850271	0.189402004	0.331704051
Beta-glutamic acid	0.112308726	0.354065049	0.650878652
L-phenylalanine	0.001310095	0.635065951	1.137837676
3-hydroxybenzoic acid	5.48021E-05	1.105456454	1.640524843
Homocystine	0.222847328	0.578934531	0.556419866
Cerotinic acid	0.792919074	-0.042477491	0.069897025
Glycerol-3-galactoside	0.027140642	1.380673855	1.354667404
tetrahydroxypentanoic acid-1,4	0.000217744	0.423267875	0.981941772
Tartaric acid	0.293949863	0.318247251	0.417647934
3-hydroxyphenylacetic acid	0.003856613	0.795333708	1.162530337
L-kynurenine	0.000262582	0.434708269	0.985650347
-2-en-1-yl-2-hydroxy-n-2-hydro	0.792755852	0.109234258	0.571111063
Cellobiose	0.000415005	0.529159999	1.087877748
Dodecanoic acid	0.146598665	0.329004724	0.583951152
D-arabinose	0.004006645	0.963607612	1.277055924
D-ribose	0.000108528	1.385733253	1.796609044
Pyrophosphate	0.000464852	-0.770662319	1.319206157
Taurine	7.37546E-05	1.40372559	1.837626348
L-asparagine	0.283875543	0.327204738	0.44776432
D-xylulose	0.001476462	0.710516274	1.24012048
4-(dimethylamino)azobenzene	0.09947914	0.94461001	1.102774397
Indoxyl sulfate	0.480638704	0.316357428	0.46343345
Dl-dopa	0.648217439	-0.22147703	0.049522167
Sophorose	0.232348111	0.335442607	0.003367813
2-hydroxy-2-pentenedioic acid	0.053515686	-3.552072652	2.146680434
Psilocin	0.000530281	-0.537701672	1.071372991

Piceatannol	0.020091093	-0.632305441	0.937132192
L-arabitol	0.095483188	0.693351778	1.157511246
Isoheptadecanoic acid	0.158258723	-2.697901679	1.019125552
6-deoxyglucitol	0.014284875	0.826234008	1.18181015
Coniferin	3.86603E-05	1.291442746	1.7596341
Myristyl myristate	0.8628017	-0.019578496	0.030290143
1-methylinosine	0.061739479	-0.566334745	0.825507098
D-fucose	0.008975545	0.85386015	1.242761043
Udp-glucuronic acid	0.298606148	0.23675901	0.458941347
Orotic acid	0.405225512	0.209867624	0.361517714
Digalacturonic acid	0.003972085	1.155387864	1.545778292
(4'-hydroxy-3'-methoxyphenyl)p	0.221355063	3.351004328	1.792756453
Aconitic acid	0.003321139	0.776020511	1.210759541
L-cystine	0.566377022	-0.133020836	0.141630418
Glycerol 3-phosphate	0.21957478	0.338338026	0.642269268
D-ribulose 5-phosphate	0.205242968	0.405646375	0.695632359
Arabinofuranose	0.877049496	0.025831672	0.074293224
Galactonic acid	0.106793269	0.382236115	0.708987971
L-glutamine	0.076070353	0.375571898	0.772700345
Ribonic acid	0.039595238	0.687273541	0.980252841
O-phosphoethanolamine	0.915888081	-0.029066796	0.07736596
Trehalose-6-phosphate	0.359378813	0.819132962	0.502465836
Glucose	0.302329224	2.295814405	1.189689802
Terephthalic acid	0.000698475	0.580249056	1.128694605
Catechin	0.006112729	0.335837649	0.763941601
Ascorbic acid	0.3976355	-0.777971558	0.112044601
D-fructose	0.581976644	0.616719494	0.160362954
eoxy-1-piperidino-alpha-d-fruct	0.000451114	0.736864912	1.271887891
D-mannose	0.787551028	0.201910364	0.362146643
3-phosphoglyceric acid	0.258912054	0.3688557	0.564237947
Palatinitol	0.670490985	0.561573469	0.575172338
Ornithine	0.523335778	-0.129009921	0.225054007
Citric acid	0.005128286	0.614040889	1.047270228

Isocitric acid	0.570276673	0.152556016	0.223418596
Citrulline	0.122392926	0.516433922	0.735370909
Guanosine	0.040956141	0.93140518	0.627907419
Epicatechin	8.00733E-06	1.437834316	1.920175908
(e)-9-tetradecenoic acid	0.461767785	0.270539818	0.420533375
N-acetyl-d-hexosamine	0.271445649	0.435999352	0.524817839
Xylofuranose	0.491033875	0.443046	0.077278655
Maltitol	0.149728592	1.047563942	1.116730828
1,5-anhydroglucitol	0.05608053	0.689785459	1.027068721
Triacetin	0.000738971	0.948404794	1.436546073
Myristic acid	0.302721123	0.263049173	0.402235442
Serotonin	0.084039167	-0.548215591	0.724404164
Chlorogenic acid	0.23761126	0.946585449	0.937651591
Quinic acid	0.002940908	1.156885299	1.567474736
Adenine	0.000690307	1.214824831	1.652096083
D-tagatose	0.027053004	0.989416919	1.18098088
D-ribose-5-phosphate	0.375416287	1.359783231	0.643604178
Delta-tocopherol	0.651385919	0.20669354	0.326359854
Carbamazepine	0.002959664	0.925635933	1.404334526
Beta-gentiobiose	0.112547108	0.639185204	1.031984817
Maltotriose	0.003313337	2.353792226	2.188236478
Lactitol	0.004407008	7.339540386	3.028710644
Galactinol	0.577371149	-0.448633898	0.140334897
Phthalimide	0.047165694	0.432555548	0.763266538
L-histidine	0.426873418	0.252460856	0.641931902
L-lysine	0.183166354	0.273850646	0.46681143
Sorbitol	0.014721226	1.77446307	2.315649603
Galacturonic acid	0.000922252	1.427450496	1.759916684
2'-deoxyguanosine	0.09860373	1.045911723	1.050476399
Ethyl beta-d-glucopyranoside	0.124734178	1.62537445	1.387229558
L-tyrosine	0.005025472	0.732880681	1.176088933
Sedoheptulose	0.000349077	1.236243524	1.674462827
Saccharic acid	0.000337772	1.269652246	1.692637402

Lignoceric acid	0.295873848	0.696391389	0.672649397
Lactobionic acid	0.000193561	1.03257373	1.550448106
Melezitose	5.46711E-05	1.148784402	1.642550216
Gallic acid	4.43653E-06	1.767822283	2.151275834
Resveratrol	0.001003929	1.377226898	1.762966006
Isohexonic acid	0.13166218	0.555274957	0.688838817
hylethyl)sulfanyl]phenyl}-2-hyd	0.142777907	0.252047416	0.575064329
Cis-gondoic acid	0.00093747	1.012798073	1.489695986
o[1,3]thiazolo[4,5-d]pyrimidine-	0.22791378	2.732636007	1.385863934
1-kestose	0.007814133	1.081025146	0.773888123
Galactose	0.233546664	0.315793272	0.456959663
Ethyl beta-d-galactofuranoside	5.04203E-05	1.050691117	1.593520337
Gluconic acid	0.000376944	1.118317556	1.589138607
Spermine	0.021394426	0.609842863	0.978630639
Acebutolol	0.008190536	0.385626158	0.827871819
Galactaric acid	0.006341838	0.897686274	1.328116541
Erucic acid	0.99700307	-0.001328753	0.103483379
Palmitelaidic acid	0.63634036	0.191160991	0.423636047
Alloxanic acid	0.819716485	0.044112152	0.078371782
,4-methylenedioxyamphetamin	0.007067142	0.57093596	0.967280543
Barbital	5.59275E-05	1.574814959	1.96805962
Montanic acid	0.251969457	0.182530309	0.424892385
Palmitic acid	0.007065761	1.696360659	1.94067766
Indole-3-propionic acid	0.811308858	0.114814912	0.091238906
Homoserine	0.007447722	1.076675194	1.435787523
P-octopamine	0.065283104	0.992401526	1.137580581
Erythrotetrofuranose	0.201469419	-2.288116709	0.317747468
Myo-inositol	0.049502975	0.434330699	0.745693808
Glycyl tyrosine	0.50912754	0.309783079	0.069790599
l-(4-morpholinyl)-alpha-d-fructo	0.901841544	0.085829761	0.13736298
Uric acid	0.004193879	1.234666871	1.610394328
Heptadecanoic acid	0.563390456	0.175980521	0.207191029
-(2-acetamido)iminodiacetic ac	0.007066413	-0.468348009	0.92331905

Alloxanoic acid	0.009074379	-0.849529896	1.260831391
2-aminoethyl methacrylate	0.223931147	-0.404539376	0.585603839
Talose	0.000247192	1.224552867	1.664173631
Caffeic acid	4.97453E-05	2.291770584	2.424687545
Cetaben	0.000882804	1.053946237	1.492158139
xy-alpha-methylnaphthaleneac	0.006020537	-0.485501238	0.943399504
Octadecanol	0.004498576	0.294433532	0.746534037
Indolelactic acid	0.123671146	0.622869091	0.124461905
Methyl beta-d-glucopyranoside	0.458050773	-0.554520872	0.80761132
7-dihydroxy-4'-methoxyisoflavo	0.111919782	0.426486092	0.733359181
1h-indole-3-acetamide	0.925484447	-0.015652411	0.003842493
Cadaverine	0.19789975	0.454436412	0.65541144
L-tryptophan	1.45181E-07	1.517599632	1.986668275
Stigmasterol	0.006325323	0.632762084	1.100149037
Linoleic acid	0.140656856	0.384834664	0.581279665
Oleic acid	0.345308135	0.216538709	0.397445104
Aprobarbital	0.425395754	-0.570006812	0.534964654
3-aminoisobutanoic acid	0.702042558	-0.125445421	0.251534149
4-hydroxyhippuric acid	0.421098348	-0.280809139	0.495612039
Bumetanide	0.859853204	0.109535862	0.18616135
Indoleacetic acid	0.193091933	1.022120122	0.514035451
dimethyl-4-hydroxy-2-pteridinar	0.759646956	0.185662618	0.168384965
Mannobiose	0.522180573	0.606373501	0.076754232
roxyestra-1,3,5(10)-triene-6,17-	4.14502E-06	1.636300885	2.039337564
2-butyl-1-octanol	0.165802651	0.495751087	0.645215669
Stearic acid	0.098433249	0.294365785	0.585350416
Cholic acid	0.84323908	-0.061508079	0.216235873
nzo[c]phenanthridinone, 11,12-	0.75568577	-0.201411766	0.045511944
D-fructose-6-phosphate	0.015115063	0.774462708	1.216977439
D-fructose-1,6-bisphosphate	0.029046975	0.690423836	1.072333286
Glucose-6-phosphate	0.014863165	0.88563944	1.300258295
Zeranol	0.759420213	0.089007939	0.086261335
L-fucose	0.565037304	0.409449974	0.209466597

Lithocholic acid	0.160608799	0.293840175	0.601840798
D-myo-inositol 4-phosphate	9.93614E-05	1.102644116	1.592169484
N-acetyl-5-hydroxytryptamine	0.463236686	-0.627486261	0.492307116
ethylene glycol monomethyl eth	0.516239501	0.094528623	0.21314944
Acetamide	0.659894127	0.065721044	0.14064946
Uridine	0.354614658	-0.456854944	0.315054213
Xanthosine	0.563142491	0.334876655	0.661524597
5-methoxytryptamine	0.394803189	0.190211439	0.219156646
a-hydroxydehydroisoandrostero	0.021926832	-0.674249729	1.056195665
Isochlorogenic acid	0.000115122	-1.169419839	1.638055514
Epigallocatechin	0.545128436	-0.186846913	0.25479289
2-monostearin	0.000305433	0.585911951	1.155505613
Docosahexaenoic acid	0.821548029	-0.059776577	0.005061412
1-monopalmitin	0.044377967	0.299806273	0.690361139
D-xylose	0.234860587	0.46352915	0.490528701
Behenic acid	0.093117601	0.57740804	1.00244778
Pectin	0.965541055	-0.02391353	0.352531352
Trisaccharide	0.035125405	0.636699272	0.929762578
Gallocatechin	0.385403831	0.498787211	0.473489123
1-monostearin	0.013038792	0.418821426	0.873745504
Inosine-5'-monophosphate	0.075307125	1.074193414	1.115660499
Gamma-tocopherol	0.009553448	1.540661104	1.56605458
Adenosine-5'-monophosphate	0.316548166	0.364670998	0.537495878
Alpha-tocopherol	0.004503488	0.799737183	1.315199448
Cholesterol	3.19738E-06	0.787316544	1.419576044
Dihydrocholesterol	0.000634873	0.811239024	1.351176024
Cholesterone	4.67895E-05	1.125130803	1.671387625
Zymosterol	0.057517246	0.541359121	0.829143573
Chenodeoxycholic acid	0.01111711	0.947905603	1.405863921
Deoxycholic acid	0.139636161	0.533193028	0.962635967
Beta-sitosterol	0.115856072	0.537765202	0.545439995
Lanosterol	0.004791935	0.896403961	1.31842683

Supplement 2A:This is evidence of group1-group4 volcanic map analysis representing metabolites. Analyzing the differences in metabolite expression in the samples.
