# Supplementary material for: Study on the Metabonomics Mechanism of Mongolian Medical Andai Therapy on Healthy People
Source: Evid Based Complement Alternat Med. 2022 Jun 20;2022:1364408. doi: 10.1155/2022/1364408 (PMC9236767; doi:10.1155/2022/1364408)
Supplement: Supplementary Materials — Supplement 1 is evidence for the principal component analysis (PCA) diagram. Supplement 2A is evidence for (group 1-group 4) female sample comparison volcanic map analysis representing metabolites, as shown in Figure 6. Supplement 2B is evidence for (group 2-group 3) male sample comparison volcanic map analysis representing metabolites, as shown in Figure 7. Supplement2C is evidence for (group 1-group 2 and group 3-group 4) full-sample comparison volcanic map analysis representing metabolites, as shown in Figure 5. Supplement 3A is evidence for (group 1A and group 4A) female sample clustering heat map analysis, as shown in Figure 9. Supplement 3B is evidence for (group 2A and group 3A) male sample clustering heat map analysis, as shown in Figure 10. Supplement 3C is evidence for (group 1A-2A and group 3A-4A) whole-sample clustering heat map analysis， as shown in Figure 8. Supplement 4A is evidence for (group 1A and group 4A) accumulation of metabolic pathways in female samples—Top20, as shown in Figure 13. Supplement 4B is evidence for (group 2 and group 3) accumulation of metabolic pathways in male samples—Top20, as shown in Figure 15. Supplement 4C is evidence for (group 1-2 and group 3-4) enrichment of metabolic pathways in the whole sample—Top20, as shown in Figure 11. Supplement 5A is evidence for (group 1 and group 4) metabolic bubble of female sample, as shown in Figure 14. Supplement 5B is evidence for (group 2 and group 3) metabolic bubble of male sample, as shown in Figure 16. Supplement 5C is evidence for metabolic pathways of (group 1-2 and group 3-4) metabolic bubble of the whole sample, as shown in Figure 12. [file 1364408.f1.zip › 1364408.f1/Supplement 2 C.pdf]

Supplement 2C

Metabolite name	P-value	log2(FC)	VIP
Ethylamine	0.150100175	0.089871377	0.406539436
Methylamine	0.000703017	0.309056291	0.951029355
Tranexamic acid	0.860123515	0.024734497	0.13802159
Diethylamine	0.562790468	0.032538904	0.203333589
Boric acid	5.86708E-06	0.646847269	1.515698118
Digitoxose	1.33471E-06	0.118787506	0.696936193
Ethylene glycol	3.11308E-07	0.05736327	0.495470895
Malonic acid	1.70487E-05	0.347991819	1.147731904
Nicotinamide	4.69384E-05	0.196875592	0.863325296
Piperidone	0.000263617	0.189581754	0.824466965
Ferulic acid	0.000213626	0.217393994	0.89270856
Dimethylethanolamine	0.001598866	0.520334958	1.198101071
2-picolinic acid	0.008075656	-0.367334825	0.858881186
Butane-2,3-diol	6.06682E-06	0.845443929	2.006932246
Norvaline	0.19287258	-0.097001342	0.336178045
3-pyridinol	0.012625125	0.162152282	0.650087654
Kynurenic acid	1.28359E-06	0.5887327	1.518207097
Pyruvic acid	0.551180328	0.116203118	0.09746058
Beta-alanine	0.0102975	0.514460256	1.131377328
Diethylcarbamic acid	0.000183869	0.221494491	0.876531936
Phenol	0.361406608	0.225147096	0.826696904
L-lactic acid	0.628649467	0.054939252	0.149510418
Maleic acid	0.210432467	0.029094823	0.194087303
Carbamic acid	0.052337137	0.410841979	0.822543321
Cyclohexylamine	5.32478E-07	-0.435112567	1.330084433
2-ketoadipic acid	0.020980643	0.183404368	0.634722196
4-methylvaleric acid	4.47264E-05	0.332727576	1.139418716
Glycolic acid	0.031813954	0.239636142	0.659691345
Sulfuric acid	3.85414E-06	0.520713985	1.417312873
Cytosin	0.001637998	0.247516648	0.88045729

2-oxo-propanoic acid	0.01244153	-1.195082082	1.021245637
Resorcinol	0.000100219	0.330914954	1.073145542
Maleimide	0.007226972	0.326341212	1.057229621
2-ketobutyric acid	0.009590989	0.413171569	1.06848781
L-alanine	0.032753159	0.297974197	0.865986516
2-ketoisovaleric acid	0.005089439	0.488837593	1.143904374
4-aminophenol	0.298954415	0.1745467	0.441668349
2-hydroxybutyric acid	0.21486456	0.325108763	0.662025544
Oxalic acid	0.551237484	0.057216511	0.180599395
Propane-1,3-diol	0.582960696	-0.046729378	0.042979881
3-hydroxypropionic acid	0.013228464	0.497786417	1.202935624
P-cresol	0.093956591	0.914754297	0.420831073
(r)-3-hydroxybutyric acid	0.486254965	0.250298999	0.412224585
2-hydroxy-3-methylbutyric acid	0.378663479	0.672555869	0.83926655
3-hydroxybutyric acid	0.516068603	-0.008736046	0.056647239
Alizarin	0.000206675	0.618539499	1.514556346
Urocanic acid	0.961249058	0.007647069	0.021506137
2-aminoisobutyric acid	0.080162902	0.297072919	0.736000055
Methanephosphonothioic acid	1.13562E-06	0.583755918	1.593827377
4-hydroxycinnamic acid	3.45557E-06	0.069702153	0.524258038
N-methylalanine	0.18015167	-0.117846049	0.324334813
N-acetylglutamate	0.002245665	0.544248808	1.384069394
3-methyl-2-oxovaleric acid	0.011376361	0.527115724	1.12083991
Succinic acid	0.026694357	0.246710616	0.706986758
Epsilon-caprolactam	0.000664609	0.435042635	1.143385375
2-methylglyceric acid	0.003047017	0.269357396	0.884667077
2-hydroxyvaleric acid	0.002737066	0.218378702	0.864862098
Thymidine	0.985263201	-0.001637922	0.029101958
3-hydroxyisovaleric acid	0.123164083	0.229173606	0.544519305
Gamma-aminobutyric acid	0.00055246	0.266370619	0.997718481
2-ketoisocaproic acid	0.008061178	0.491316459	1.24812097
L-valine	0.032911879	0.315340231	0.860905796
Hypoxanthine	0.616592932	0.066971839	0.172462699

Cyano-l-alanine	0.179437651	-0.105285301	0.357250255
Adipic acid	0.13549906	0.389355967	0.280057795
L-alanine-alanine	0.001743057	0.157347739	0.715487366
Dopamine	0.027152006	0.118912599	0.512302735
2-piperidinobenzonitrile	0.106394735	0.379789333	0.996297126
2-hydroxy-3-methylpentanoic acid	7.0283E-06	0.243915297	0.993102776
Urea	0.028695618	0.221676992	0.836164528
Amine	0.092103399	0.496407609	0.147985834
Benzoic acid	0.000791062	0.532623478	1.212341441
Sinapinic acid	1.75308E-06	0.357293167	1.177588047
N-methylglutamic acid	0.777810451	-0.068514368	0.100454792
1-methylhydantoin	0.256942926	2.926046917	1.750336892
Ethanolamine	0.028820427	0.038899637	0.286299687
Leucine	0.0090705	0.504288935	1.190378136
Ciliatine	0.005616286	0.195901017	0.714247624
Glycerol	0.086577134	0.417442768	0.494513227
Pyridoxine	0.643819241	0.065283589	0.157215421
Citraconic acid	0.328725088	0.117196329	0.253107943
Dodecanoic acid, isopropanol ester	0.000536803	0.16233946	0.74620782
Pyrazin-2-carboxylic acid	1.7934E-10	1.029316031	2.239357274
3,6-anhydro-d-galactose	0.025814809	-0.406993847	0.816738746
L-isoleucine	0.003314926	0.699442304	1.492298892
L-proline	0.425842129	0.15819978	0.542619618
Glycine	0.032335526	0.500871211	1.061204367
Hydroquinone	0.334471038	-0.094387341	0.288058041
Xanthine	0.586099226	0.068136014	0.190963599
Allantoin	0.174858279	0.289165337	0.543244681
Pyrrole-2-carboxylic acid	0.173264573	0.097004458	0.397021319
Butanedioic acid	0.592779603	0.101991691	0.325311134
Metharbital	0.000546061	0.71905378	1.488982771
Oleamide	1.40711E-06	0.454561178	1.367507722
Glucosamine	0.211716251	-0.264487218	0.315537479
Phytol	7.90421E-05	0.224897912	0.911922793

Glyceric acid	0.121123599	0.327023493	0.531468787
Oxoproline	0.569466314	0.337118955	0.085961876
Uracil	0.000108445	0.615439524	1.528793395
L-threonic acid	0.038477475	-0.86221449	1.186808647
1,2,4-benzenetriol	1.94445E-07	0.478586204	1.400389222
4-deoxyerythronic acid	0.017498504	0.656436326	1.392039244
Fumaric acid	0.780742091	-0.066130364	0.206949271
Pinitol	0.61533364	-0.086981703	0.080281973
Formononetin	0.057122394	0.354320215	0.933744532
Erythrose	0.589433273	-0.088476073	0.151954044
Serine	0.004283108	0.362317403	1.085931944
Dihydroxymalonic acid	0.0262692	0.730104309	1.290147197
Pelargonic acid	0.007218691	0.208936552	0.719214323
D-erythro-sphingosine	0.015279571	0.325771968	0.838440851
Phytosphingosine	0.003386922	0.322946832	1.035250149
Udp-n-acetylglucosamine	0.003122866	0.332054874	1.059253457
Shikimic acid	0.642974455	-0.182230536	0.603345172
O-phosphoserine	0.379576115	0.169768592	0.217391371
L-threonine	0.022268263	0.400726498	0.986818806
Hydroxypropanedioic acid	0.122053244	0.340883033	0.874955366
Abietic acid	0.001583165	-0.370388598	1.062476497
2,3-dihydroxy-acrylic acid	0.124550182	0.274604485	0.650089644
N-carbamoylaspartate	0.003700542	0.968519523	1.600692938
Glutaric acid	0.389052713	0.222235617	0.482533657
Glycyl proline	0.925421951	0.027870573	0.764666689
Putrescine	3.62917E-05	0.238770377	0.934419434
1,2,4-butanetriol	0.548199192	-0.141036586	0.509331739
(s)-3,4-dihydroxybutyric acid	0.000456049	0.630830984	1.50609399
D-fructose-1-phosphate	0.071493379	0.176641027	0.559956319
N-methyl-l-leucine	0.094409657	1.027003739	1.501886888
2,4-diaminobutyric acid	0.001946528	-0.631650363	1.403794612
9-decenoic acid	0.172282569	0.696363092	0.801708082
Phosphenodiimidic amide	0.010030127	0.700070678	1.21321133

Cytidine-5-monophosphate	0.136025142	0.378493799	0.6736123
Capric acid	0.000151275	0.237859346	0.908051115
Adrenaline	0.201859872	-1.800097023	0.39905822
Aminomalonate	0.004068225	0.649047753	1.374229031
L-glutamine dehydrated	0.080461653	0.268911043	0.787699631
1,3-dihydroxypyridine	0.019041492	-0.195353291	0.649791038
L-cysteine-glycine	0.016007872	0.246007933	0.739770203
Biphenyl	0.459810787	-0.234543272	0.008037647
Noradrenaline	0.272562048	-0.243761681	0.519554674
Malic acid	0.545677345	0.163372319	0.37316783
N-acetylornithine	0.193019544	-0.109368876	0.408444636
Tetracosane	0.047807835	0.016852826	0.170942108
Scopoletin	0.000430482	0.200344855	0.82388197
Galactitol	0.006412215	0.659346149	1.333223197
N-acetylputrescine	0.28084477	-0.081547598	0.330795103
Erythritol	0.040644179	0.315010186	0.871415819
Tromethamine	0.044339182	0.332814131	0.801001725
L-methionine	0.001344405	0.633085575	1.459075218
Dehydroascorbic acid	0.000207706	0.490749846	1.341491657
L-aspartic acid	0.048111117	0.305313337	0.84557303
4-hydroxyproline	0.440712207	0.273092256	0.182803863
Pentadecanol	0.213021762	0.251869765	0.938043038
2,6-bis(tert-butyl)phenol	0.000496025	0.29388316	1.002986248
Aminoadipic acid	0.295907021	0.342391752	0.252625415
Creatinine	0.018414606	0.446926808	0.917449916
L-cysteine	0.232407394	0.196177216	0.75196026
Glucosaminic acid	0.015310667	-0.397863502	1.31916291
Erythronic acid	0.31281222	-0.223457922	0.696012923
4-acetamino phenol	0.12834896	0.571072174	0.854485195
Naproxen	0.031177709	0.583716407	1.284698944
L-2-hydroxyglutaric acid	0.322947938	0.184028646	0.455104159
Oxoglutaric acid	0.97832435	0.014625493	0.699031376
5-hydroxy-3-indoleacetic acid	0.002153538	0.685715897	1.491762336

Nicotianamine	0.059112812	0.617632942	1.085268743
Dithiothreitol	0.027331404	0.193534268	0.699565502
Allantoic acid	0.215212254	0.134142769	0.474115907
N-acetyl-l-aspartic acid	0.202315781	0.477966946	0.36286645
Cyanuric acid	0.09274634	2.683087484	2.155488373
Glucose-1-phosphate	3.61125E-05	0.608265605	1.553299466
Xylonolactone	0.320580327	0.286693543	0.987291939
L-glutamic acid	0.174512951	0.309787108	0.646459371
Beta-glutamic acid	0.011092343	0.383839343	0.841349566
L-phenylalanine	0.001053695	0.453336918	1.233933845
3-hydroxybenzoic acid	2.92884E-05	0.682649461	1.650562802
Homocystine	0.257593744	0.423628848	0.693864705
Cerotinic acid	0.359109067	0.097500345	0.280482381
Glycerol-3-galactoside	0.003974774	1.059081205	1.60682356
2,3,4,5-tetrahydroxypentanoic acid-1,4-lactone	5.32745E-05	0.281333892	1.000362538
Tartaric acid	0.403123241	0.15814995	0.538600088
3-hydroxyphenylacetic acid	0.002980566	0.60238673	1.249749688
L-kynurenine	4.16726E-05	0.299102147	1.036435491
nethoxy-5-prop-2-en-1-yl-2-hydroxy-n-2-hydroxyethylbenzam	0.421104471	0.22666697	0.64336901
Cellobiose	0.023173792	0.265579789	0.634798441
Dodecanoic acid	0.076491636	0.535752518	0.964417605
D-arabinose	0.000828174	0.773791217	1.465587403
D-ribose	6.91065E-05	1.020285822	2.072520026
Pyrophosphate	0.071396673	-0.302450431	0.927974963
Taurine	8.57495E-05	0.926296265	1.946569128
L-asparagine	0.150423804	0.284433346	0.614231491
D-xylulose	9.47173E-05	0.526029486	1.342467861
4-(dimethylamino)azobenzene	0.094855564	0.609298754	0.935587194
Indoxyl sulfate	0.085240308	0.527699717	0.870518696
Dl-dopa	0.964827682	0.011200949	0.24726121
Sophorose	0.367235103	0.180375122	0.016521173
2-hydroxy-2-pentenedioic acid	0.010495294	-1.976723252	2.021067214
Psilocin	0.052137284	-0.236972204	0.757441522

Piceatannol	0.002308628	-0.482416724	1.075224935
L-arabitol	0.211087528	0.311136001	0.845410831
Isoheptadecanoic acid	0.903998903	-0.101747338	0.257986235
6-deoxyglucitol	0.0006435	0.623217024	1.3854333
Coniferin	3.82595E-05	0.82458868	1.799306347
Myristyl myristate	0.28989354	-0.087687683	0.280572451
1-methylinosine	0.012922576	-0.433846275	0.910366686
D-fucose	0.000330545	0.641682738	1.386660572
Udp-glucuronic acid	0.266393975	0.167279048	0.500353994
Orotic acid	0.017593963	0.492499069	0.937975517
Digalacturonic acid	0.003838797	0.735544809	1.397601549
3-hydroxy-3-(4'-hydroxy-3'-methoxyphenyl)propionic acid	0.165295967	2.392245614	1.710031706
Aconitic acid	2.1654E-05	0.645571434	1.586553163
L-cystine	0.793417763	-0.04134143	0.228899401
Glycerol 3-phosphate	0.154451832	0.338047212	0.798536051
D-ribulose 5-phosphate	0.394527304	0.176348403	0.479455075
Arabinofuranose	0.777153814	-0.042368908	0.148328766
Galactonic acid	0.090862033	0.275992896	0.642589229
L-glutamine	0.310447131	0.165945981	0.527550663
Ribonic acid	0.073689164	0.342328398	0.75275384
O-phosphoethanolamine	0.182359948	-0.256494738	0.511609021
Trehalose-6-phosphate	0.248246493	0.504843821	0.636663088
Glucose	0.31640256	1.274000059	0.948226677
Terephthalic acid	0.00017932	0.383040143	1.139713811
Catechin	0.712390074	0.059772737	0.364638594
Ascorbic acid	0.384580016	-0.512151503	0.348459825
D-fructose	0.731868771	0.220187648	0.177320152
1-deoxy-1-piperidino-alpha-d-fructose	0.000122376	0.59598051	1.487169688
D-mannose	0.489084651	-0.293842481	0.755268906
3-phosphoglyceric acid	0.091039572	0.372538814	0.812995909
Palatinitol	0.87982083	0.117116246	0.524556789
Ornithine	0.947627011	-0.015517195	0.14314479
Citric acid	0.000118751	0.495641394	1.340696683

Isocitric acid	0.108904709	0.28168527	0.628903008
Citrulline	0.046482096	0.472667718	0.9395639
Guanosine	0.021101505	0.627783937	0.94512264
Epicatechin	1.96978E-06	0.942094575	2.048708191
(e)-9-tetradecenoic acid	0.492110323	0.228618741	0.641324859
N-acetyl-d-hexosamine	0.31830553	0.2508957	0.599626065
Xylofuranose	0.655752418	0.145415265	0.006676977
Maltitol	0.145052202	0.642397274	0.939470399
1,5-anhydroglucitol	0.012846936	0.588728938	1.276274821
Triacetin	0.000714085	0.646088139	1.537615729
Myristic acid	0.286466136	0.304659459	0.661728366
Serotonin	0.006294732	-0.536078081	1.033340102
Chlorogenic acid	0.087609408	0.825768786	1.385003245
Quinic acid	0.006522546	0.952587753	1.641953582
Adenine	0.000533018	0.711061491	1.570869358
D-tagatose	0.021640697	0.526232909	1.080381066
D-ribose-5-phosphate	0.543740407	-0.54325599	0.673533898
Delta-tocopherol	0.222276156	0.295597695	0.601441963
Carbamazepine	0.002538364	0.661705099	1.434834181
Beta-gentiobiose	0.062097747	0.418204145	0.998001473
Maltotriose	0.106059483	0.730335675	1.547814243
Lactitol	0.107259048	1.564945259	1.838860565
Galactinol	0.346674964	-0.384658081	0.331612484
Phthalimide	0.013348473	0.327585503	0.901365673
L-histidine	0.971188352	0.011013929	0.278426766
L-lysine	0.322583939	0.180118659	0.479832987
Sorbitol	0.113177292	0.905169479	1.931643066
Galacturonic acid	0.000406314	0.891646249	1.864662441
2'-deoxyguanosine	0.165575656	0.442364849	0.682354852
Ethyl beta-d-glucopyranoside	0.198450276	0.879277564	0.517904168
L-tyrosine	0.040593098	0.413652184	1.121455564
Sedoheptulose	0.000210493	0.807145843	1.571676767
Saccharic acid	0.000356965	0.769044703	1.704794157

Lignoceric acid	0.05810164	0.649890984	1.05479244
Lactobionic acid	0.006585718	0.537327633	1.317540418
Melezitose	0.027526623	0.444787636	1.258686215
Gallic acid	1.27189E-06	1.14459778	2.275333652
Resveratrol	0.003398592	0.75447361	1.553584742
Isohexonic acid	0.150591815	0.330520479	0.71251957
hyl-2-{4-[(1-methylethyl)sulfanyl]phenyl}-2-hydroxy]ethyl)octar	0.829096754	-0.04006633	0.129355399
Cis-gondoic acid	0.00715955	0.503048147	1.353083114
2-amino[1,3]thiazolo[4,5-d]pyrimidine-5,7-diol	0.125737402	2.018084003	1.421169603
1-kestose	0.119587266	0.363256628	0.530919252
Galactose	0.295746209	0.150597758	0.399977478
Ethyl beta-d-galactofuranoside	0.019113362	0.44421282	1.165626921
Gluconic acid	0.003655606	0.619089794	1.369516095
Spermine	0.003292531	0.773180138	1.454220908
Acebutolol	0.078641929	0.259323257	0.774103885
Galactaric acid	0.002260462	0.584807916	1.397653104
Erucic acid	0.563482753	0.151298387	0.000868221
Palmitelaidic acid	0.773688739	-0.148504316	0.324808604
Alloxanic acid	0.973465441	0.004351944	0.030981513
3,4-methylenedioxyamphetamine	0.069161498	0.342486823	0.495016182
Barbital	0.000391743	1.117070097	1.583987764
Montanic acid	0.221160284	0.140277914	0.434895178
Palmitic acid	0.287121001	0.738100927	1.954468524
Indole-3-propionic acid	0.415072321	-1.091786951	0.471983532
Homoserine	0.111127555	0.497140096	1.255775229
P-octopamine	0.215804374	-1.882018561	0.081117125
Erythrotetrofuranose	0.845692044	-0.2370869	0.359274637
Myo-inositol	0.019115849	0.318314781	0.863551191
Glycyl tyrosine	0.089661819	0.524638285	0.542907731
1-deoxy-1-(4-morpholinyl)-alpha-d-fructopyranose	0.803759738	-0.104538483	0.188824296
Uric acid	0.001462685	0.801234192	1.758847227
Heptadecanoic acid	0.019410609	0.608780694	0.939636374
N-(2-acetamido)iminodiacetic acid	0.126999637	-0.204097814	0.662555184

Alloxanoic acid	0.120200909	-0.338167754	0.970131465
2-aminoethyl methacrylate	0.280678664	0.386811704	0.247813301
Talose	0.041584283	0.463310555	1.198625634
Caffeic acid	9.32795E-06	1.454836793	2.548536797
Cetaben	0.031498517	1.109281439	1.598385641
6-hydroxy-alpha-methylnaphthaleneacetic acid	0.075424012	-0.272738489	0.741138937
Octadecanol	0.02569931	0.17273595	0.66920232
Indolelactic acid	0.042091551	0.646187682	0.391409627
Methyl beta-d-glucopyranoside	0.199909823	-0.604463748	0.617007811
5,7-dihydroxy-4'-methoxyisoflavone	0.040713954	0.40697922	0.796063725
1h-indole-3-acetamide	0.799357549	-0.052258102	0.061630248
Cadaverine	0.042472859	0.783851099	1.154599751
L-tryptophan	4.84258E-06	1.001457035	2.148134842
Stigmasterol	0.021763774	0.388520122	1.097502742
Linoleic acid	0.215961451	0.384813976	0.6151568
Oleic acid	0.585239156	0.123457701	0.322282708
Aprobarbital	0.403805815	-0.361415109	0.301977075
3-aminoisobutanoic acid	0.515282936	0.248094444	0.026464587
4-hydroxyhippuric acid	0.808005466	-0.06970137	0.310779768
Bumetanide	0.690002229	-0.165217859	0.216894594
Indoleacetic acid	0.121757716	0.870641562	0.556238176
6,7-dimethyl-4-hydroxy-2-pteridinamine	0.711177959	0.131813235	0.031959363
Mannobiose	0.454864562	0.40764705	0.227472286
3-hydroxyestra-1,3,5(10)-triene-6,17-dione	5.726E-07	1.132137385	2.248722261
2-butyl-1-octanol	0.59871686	0.112732611	0.343004753
Stearic acid	0.089718595	0.323180706	0.74288527
Cholic acid	0.293261927	-0.184371865	0.495081516
6(5h)-benzo[c]phenanthridinone, 11,12-dihydro-	0.553624783	-0.229168148	0.040534657
D-fructose-6-phosphate	0.061452213	0.406548219	0.975235092
D-fructose-1,6-bisphosphate	0.012792921	0.487342849	1.107595391
Glucose-6-phosphate	0.039069133	0.479987234	1.119087606
Zeranol	0.697837189	0.066987637	0.125479974
L-fucose	0.419559566	0.317324043	0.510434876

Lithocholic acid	0.490166755	0.121186729	0.361045923
D-myo-inositol 4-phosphate	0.000665839	0.641242657	1.511456835
N-acetyl-5-hydroxytryptamine	0.638631255	-0.27739002	0.582623594
Triethylene glycol monomethyl ether	0.343463297	0.105128124	0.303890129
Acetamide	0.415948498	0.093591607	0.254773363
Uridine	0.202615348	-0.578209244	0.290883158
Xanthosine	0.884182294	0.050762539	0.392579559
5-methoxytryptamine	0.124279894	0.358877073	0.558863313
16a-hydroxydehydroisoandrosterone	0.189903131	-0.295213634	0.773687017
Isochlorogenic acid	0.000217397	-0.648554489	1.595420399
Epigallocatechin	0.68938038	0.076418265	0.020186155
2-monostearin	2.29342E-05	0.536134408	1.450563569
Docosahexaenoic acid	0.554899403	0.165752588	0.273819636
1-monopalmitin	0.023331967	0.212361064	0.702819466
D-xylose	0.053908734	0.424385842	0.798991452
Behenic acid	0.144942777	0.328908052	0.925697063
Pectin	0.296628076	0.318903207	0.288542168
Trisaccharide	0.010626829	0.608232626	1.247981138
Gallocatechin	0.413622421	0.292469884	0.206829234
1-monostearin	0.005007554	0.29642376	0.891317583
Inosine-5'-monophosphate	0.012896862	0.805836789	1.400207604
Gamma-tocopherol	0.012134295	0.853209893	1.145106924
Adenosine-5'-monophosphate	0.065042638	0.441698458	0.869178721
Alpha-tocopherol	0.004035398	0.535368528	1.152001419
Cholesterol	1.93368E-05	0.481148397	1.381408895
Dihydrocholesterol	0.410953792	-1.332244584	0.310831298
Cholesterone	3.07741E-05	0.772824669	1.750690858
Zymosterol	0.127942597	0.312235359	0.487998896
Chenodeoxycholic acid	0.000526331	0.760037146	1.710604362
Deoxycholic acid	0.104789183	0.607037462	0.892210473
Beta-sitosterol	0.047426021	0.419392845	0.785701593
Lanosterol	0.010212453	0.611180997	1.53985152

Supplement 2C:This is evidence of group1-group2&group3-group4 volcanic map analysis representing metabolites. Analyzing the differences in metabolite expression in the samples.
