# Supplementary material for: Study on the Metabonomics Mechanism of Mongolian Medical Andai Therapy on Healthy People
Source: Evid Based Complement Alternat Med. 2022 Jun 20;2022:1364408. doi: 10.1155/2022/1364408 (PMC9236767; doi:10.1155/2022/1364408)
Supplement: Supplementary Materials — Supplement 1 is evidence for the principal component analysis (PCA) diagram. Supplement 2A is evidence for (group 1-group 4) female sample comparison volcanic map analysis representing metabolites, as shown in Figure 6. Supplement 2B is evidence for (group 2-group 3) male sample comparison volcanic map analysis representing metabolites, as shown in Figure 7. Supplement2C is evidence for (group 1-group 2 and group 3-group 4) full-sample comparison volcanic map analysis representing metabolites, as shown in Figure 5. Supplement 3A is evidence for (group 1A and group 4A) female sample clustering heat map analysis, as shown in Figure 9. Supplement 3B is evidence for (group 2A and group 3A) male sample clustering heat map analysis, as shown in Figure 10. Supplement 3C is evidence for (group 1A-2A and group 3A-4A) whole-sample clustering heat map analysis， as shown in Figure 8. Supplement 4A is evidence for (group 1A and group 4A) accumulation of metabolic pathways in female samples—Top20, as shown in Figure 13. Supplement 4B is evidence for (group 2 and group 3) accumulation of metabolic pathways in male samples—Top20, as shown in Figure 15. Supplement 4C is evidence for (group 1-2 and group 3-4) enrichment of metabolic pathways in the whole sample—Top20, as shown in Figure 11. Supplement 5A is evidence for (group 1 and group 4) metabolic bubble of female sample, as shown in Figure 14. Supplement 5B is evidence for (group 2 and group 3) metabolic bubble of male sample, as shown in Figure 16. Supplement 5C is evidence for metabolic pathways of (group 1-2 and group 3-4) metabolic bubble of the whole sample, as shown in Figure 12. [file 1364408.f1.zip › 1364408.f1/Supplement 2B.pdf]

Supplement 2B

Metabolite name	P-value	log2(FC)	VIP
Ethylamine	0.804968544	0.022545457	0.083095939
Methylamine	0.280771897	0.145673359	0.498052705
Tranexamic acid	0.726259437	-0.067742347	0.101362738
Diethylamine	0.915756394	-0.008263444	0.047322758
Boric acid	0.007449974	0.468972273	1.579090259
Digitoxose	0.014923488	0.065718462	0.556195174
Ethylene glycol	0.006666521	0.041425074	0.506123709
Malonic acid	0.02090273	0.138341528	0.800914569
Nicotinamide	0.111413835	0.090603895	0.5382123
Piperidone	0.192125772	0.085304093	0.459909967
Ferulic acid	0.203352398	0.104150395	0.510685056
Dimethylethanolamine	0.042401299	0.246575842	1.024870735
2-picolinic acid	0.075241921	-0.296219819	1.089160813
Butane-2,3-diol	0.053817421	0.400087357	1.305800231
Norvaline	0.353883877	-0.096343696	0.480278212
3-pyridinol	0.873639189	0.01284355	0.020249381
Kynurenic acid	0.003279889	0.363751458	1.49144024
Pyruvic acid	0.178543741	0.332181414	0.851383638
Beta-alanine	0.084031798	0.13858641	0.653994316
Diethylcarbamic acid	0.158417025	0.08066809	0.474060359
Phenol	0.903646969	-0.051439457	0.456614366
L-lactic acid	0.44917054	0.072778593	0.298087751
Maleic acid	0.375793704	0.039647746	0.269099031
Carbamic acid	0.567170232	0.123072609	0.444833047
Cyclohexylamine	0.000805374	-0.317863856	1.503909312
2-ketoadipic acid	0.684766486	-0.032853068	0.207311328
4-methylvaleric acid	0.087694531	0.211462965	0.969336909
Glycolic acid	0.022367202	0.306935034	1.22498167
Sulfuric acid	0.003332698	0.301434308	1.346394935
Cytosin	0.57789186	0.041303356	0.174127734

2-oxo-propanoic acid	0.038204357	-1.244629476	2.137548045
Resorcinol	0.027507806	0.205139178	0.946233904
Maleimide	0.900640298	-0.018670613	0.198917847
2-ketobutyric acid	0.083614841	0.420220496	1.258134756
L-alanine	0.252737072	0.180772976	0.620035321
2-ketoisovaleric acid	0.293047791	0.260147821	0.744156886
4-aminophenol	0.312696997	0.258697731	0.756937475
2-hydroxybutyric acid	0.28960502	0.366626636	0.86373781
Oxalic acid	0.760473066	0.029438895	0.104537797
Propane-1,3-diol	0.325847021	-0.106484291	0.571185853
3-hydroxypropionic acid	0.530784462	0.136481184	0.576146593
P-cresol	0.636105947	0.30831296	0.012999134
(r)-3-hydroxybutyric acid	0.384190732	0.395779609	0.552240032
2-hydroxy-3-methylbutyric acid	0.445196423	0.73365793	0.906893384
3-hydroxybutyric acid	0.991458954	-0.000201084	0.03361029
Alizarin	0.070887649	0.437630802	1.287584198
Urocanic acid	0.70571459	0.066321216	0.360447902
2-aminoisobutyric acid	0.124313376	0.401706609	1.139247255
Methanephosphonothioic acid	0.017664702	0.335661383	1.355759115
4-hydroxycinnamic acid	0.008460853	0.045771725	0.520604641
N-methylalanine	0.259385494	-0.133973761	0.578568737
N-acetylglutamate	0.099553753	0.388431189	1.254772808
3-methyl-2-oxovaleric acid	0.029097431	0.533989725	1.619977239
Succinic acid	0.843736634	0.016551245	0.03569175
Epsilon-caprolactam	0.113003395	0.368961537	1.130798711
2-methylglyceric acid	0.374193872	0.076614894	0.317665479
2-hydroxyvaleric acid	0.26744194	0.101774604	0.475875407
Thymidine	0.936738388	-0.009118077	0.025836606
3-hydroxyisovaleric acid	0.088461519	0.293130867	1.090348668
Gamma-aminobutyric acid	0.245767496	0.107518799	0.499732799
2-ketoisocaproic acid	0.214287784	0.279489391	0.95403402
L-valine	0.16840326	0.298092826	0.945980282
Hypoxanthine	0.456485853	0.138248796	0.54949879

Cyano-l-alanine	0.014229353	-0.279439226	1.308639545
Adipic acid	0.971762646	-0.012485314	0.816126867
L-alanine-alanine	0.286684758	0.06731044	0.347019352
Dopamine	0.509912419	0.045476225	0.229303954
2-piperidinobenzonitrile	0.309715654	0.293554827	1.026863517
2-hydroxy-3-methylpentanoic acid	0.045908982	0.112717768	0.682378646
Urea	0.251696989	0.233152413	1.727017194
Amine	0.951109282	-0.029016382	1.347784657
Benzoic acid	0.135367463	0.34891292	1.084805521
Sinapinic acid	0.008532305	0.292817802	1.258721599
N-methylglutamic acid	0.725382101	-0.055572391	0.358174139
1-methylhydantoin	0.200066095	0.662718431	1.538402346
Ethanolamine	0.180583959	0.030550397	0.296315408
Leucine	0.109756354	0.419976069	1.133848324
Ciliatine	0.370679813	0.080686642	0.323576866
Glycerol	0.58112613	0.262863775	0.220472898
Pyridoxine	0.463333474	0.143212707	0.552362273
Citraconic acid	0.034996371	0.296638401	1.147365822
Dodecanoic acid, isopropanol ester	0.12278134	0.090784544	0.514667487
Pyrazin-2-carboxylic acid	0.000325652	0.697749466	2.237143373
3,6-anhydro-d-galactose	0.08994945	-0.415436259	1.362499253
L-isoleucine	0.04025415	0.707116063	1.782457111
L-proline	0.7975824	0.061066204	0.031795488
Glycine	0.579569722	0.149075565	0.502943741
Hydroquinone	0.517071009	-0.080570152	0.369162631
Xanthine	0.541967721	0.10679051	0.424014438
Allantoin	0.746706454	0.043724602	0.110706643
Pyrrole-2-carboxylic acid	0.800597058	0.023596532	0.023268073
Butanedioic acid	0.750148321	0.084567954	0.176435777
Metharbital	0.015198604	0.576529111	1.909044337
Oleamide	0.010515861	0.307206701	1.248228317
Glucosamine	0.238309775	-0.335210926	0.889859938
Phytol	0.00559854	0.186732516	1.048436617

Glyceric acid	0.323151132	0.227993919	0.638396915
Oxoproline	0.544602451	0.244831383	0.678976559
Uracil	0.039101567	0.423050585	1.468394766
L-threonic acid	0.097110437	-0.878117181	1.648528267
1,2,4-benzenetriol	0.002875329	0.296141848	1.316529239
4-deoxyerythronic acid	0.059278328	0.587940494	1.670866047
Fumaric acid	0.867839952	0.047475815	0.121646595
Pinitol	0.056751525	-0.4175274	1.676834404
Formononetin	0.27344028	0.249708859	0.720271494
Erythrose	0.831121172	-0.036220153	0.13932565
Serine	0.382311833	0.135457293	0.445396729
Dihydroxymalonic acid	0.026536092	0.243803531	1.034503219
Pelargonic acid	0.181696887	0.148203477	0.629076338
D-erythro-sphingosine	0.499854764	0.122640497	0.205746727
Phytosphingosine	0.365618531	0.095434272	0.375428216
Udp-n-acetylglucosamine	0.227963225	0.129007151	0.525182541
Shikimic acid	0.071349938	-0.85209714	2.241269443
O-phosphoserine	0.37813287	0.203033551	0.460290709
L-threonine	0.103403864	0.418921772	1.053671106
Hydroxypropanedioic acid	0.521653654	0.200859184	0.621146089
Abietic acid	0.054876026	-0.249921506	1.107502978
2,3-dihydroxy-acrylic acid	0.780517867	-0.03689773	0.082017122
N-carbamoylaspartate	0.111717279	0.555716873	1.838962787
Glutaric acid	0.572694322	0.170517151	0.533365465
Glycyl proline	0.206045123	-0.445290438	1.350847676
Putrescine	0.131746231	0.109688918	0.557735006
1,2,4-butanetriol	0.700485493	-0.088792295	0.440100901
(s)-3,4-dihydroxybutyric acid	0.093105115	0.352258735	1.117161052
D-fructose-1-phosphate	0.276065522	0.105922199	0.479804518
N-methyl-l-leucine	0.349606771	0.590392882	0.993820091
2,4-diaminobutyric acid	0.043543946	-0.601097741	1.826124167
9-decenoic acid	0.232550241	0.947900149	1.408637236
Phosphenodiimidic amide	0.453437977	0.184569138	0.340888115

Cytidine-5-monophosphate	0.205048138	0.381698982	1.300586018
Capric acid	0.038659883	0.168057782	0.841585458
Adrenaline	0.445197684	-1.456980769	0.346113288
Aminomalonate	0.117454574	0.380072643	1.191733982
L-glutamine dehydrated	0.324364266	-0.208121082	0.787615549
1,3-dihydroxypyridine	0.143813975	-0.155036431	0.802497992
L-cysteine-glycine	0.404613096	0.113593928	0.363587644
Biphenyl	0.294733821	-0.537176119	1.158094988
Noradrenaline	0.456371731	-0.139701621	0.897371242
Malic acid	0.481265422	0.211906235	0.568875229
N-acetylornithine	0.99922041	-7.79673E-05	0.013993192
Tetracosane	0.127120128	0.017949674	0.253619396
Scopoletin	0.144400417	0.104213386	0.528281501
Galactitol	0.328664349	0.331605325	0.914669486
N-acetylputrescine	0.928584992	0.00710123	0.019389611
Erythritol	0.829981217	0.039237222	0.057384678
Tromethamine	0.606683863	-0.098410226	0.379387269
L-methionine	0.106363511	0.462902181	1.105543936
Dehydroascorbic acid	0.047310191	0.337351929	1.131451586
L-aspartic acid	0.282229547	0.210358624	0.70158292
4-hydroxyproline	0.230902502	0.496905792	1.065327639
Pentadecanol	0.88660351	-0.047614004	0.459142281
2,6-bis(tert-butyl)phenol	0.24546839	0.103956639	0.504146034
Aminoadipic acid	0.303596029	0.418247855	1.163379095
Creatinine	0.110059132	0.425264757	1.101211295
L-cysteine	0.885473619	-0.033886161	0.14002807
Glucosaminic acid	0.119911247	-0.33108789	1.525820901
Erythronic acid	0.730124275	0.1067667	0.010956476
4-acetamino phenol	0.457411629	0.110938811	0.368117323
Naproxen	0.153755734	0.489141389	0.999197263
L-2-hydroxyglutaric acid	0.323603328	0.26099077	0.657468886
Oxoglutaric acid	0.824204749	-0.155295608	0.292177986
5-hydroxy-3-indoleacetic acid	0.018404065	0.655109805	1.844490291

Nicotianamine	0.037979251	0.825444856	2.103794342
Dithiothreitol	0.391100392	0.092573904	0.464977372
Allantoic acid	0.823538522	-0.019998315	0.070794454
N-acetyl-l-aspartic acid	0.374840338	0.458045249	1.031095713
Cyanuric acid	0.30828536	1.365928551	1.149696441
Glucose-1-phosphate	0.525656542	0.092560208	0.279044176
Xylonolactone	0.797946927	0.028784054	0.065260624
L-glutamic acid	0.189226014	0.384053846	0.988079662
Beta-glutamic acid	0.061935639	0.411928423	1.123633667
L-phenylalanine	0.128978224	0.295149926	0.865026705
3-hydroxybenzoic acid	0.008142552	0.22905663	1.066972468
Homocystine	0.495820933	0.336587357	0.976588201
Cerotinic acid	0.075686709	0.216302527	0.909818076
Glycerol-3-galactoside	0.093445194	0.756829153	1.915645433
2,3,4,5-tetrahydroxypentanoic acid-1,4-lactone	0.070461923	0.142038061	0.699689322
Tartaric acid	0.964329169	-0.010900767	0.694290677
3-hydroxyphenylacetic acid	0.119773349	0.450789511	1.122831809
L-kynurenine	0.041027996	0.15870294	0.821325471
y-5-prop-2-en-1-yl-2-hydroxy-n-2-hydroxyethylb	0.381694476	0.300973069	0.472973711
Cellobiose	0.979119967	-0.004562257	0.232539975
Dodecanoic acid	0.163661096	0.688308084	1.431601685
D-arabinose	0.045278928	0.631490951	1.614200518
D-ribose	0.031048047	0.762655693	1.982437499
Pyrophosphate	0.871139388	-0.019288502	0.168090267
Taurine	0.11614083	0.504123903	1.275114443
L-asparagine	0.306197475	0.252003696	0.562354881
D-xylulose	0.030854401	0.337409186	1.235825506
4-(dimethylamino)azobenzene	0.470613305	0.100567944	0.349161181
Indoxyl sulfate	0.087155909	0.770230262	1.787206533
Dl-dopa	0.481947065	0.161772951	0.523598252
Sophorose	0.783372649	0.069403108	0.405710097
2-hydroxy-2-pentenedioic acid	0.108743178	-1.106703629	1.691801846
Psilocin	0.858891141	-0.018957709	0.181896817

Piceatannol	0.04354553	-0.359310129	1.279418843
L-arabitol	0.863680648	-0.052330749	0.128765589
Isoheptadecanoic acid	0.534107443	0.625104056	0.655607006
6-deoxyglucitol	0.016235012	0.453369859	1.419345009
Coniferin	0.075300594	0.44935814	1.292950275
Myristyl myristate	0.165386818	-0.147268974	0.772795506
1-methylinosine	0.092671222	-0.327116021	1.142613794
D-fucose	0.016237446	0.446954132	1.457431625
Udp-glucuronic acid	0.598868699	0.107236658	0.497640273
Orotic acid	0.002815558	0.676097306	1.985411003
Digalacturonic acid	0.242566565	0.206212904	0.54192962
lroxy-3-(4'-hydroxy-3'-methoxyphenyl)propionic	0.377035436	0.879435664	0.890578313
Aconitic acid	0.002275694	0.532350463	1.865248844
L-cystine	0.850036238	0.043361348	0.344933511
Glycerol 3-phosphate	0.245472448	0.337868948	0.855274935
D-ribulose 5-phosphate	0.96070323	0.012133367	0.177819396
Arabinofuranose	0.644816741	-0.094258676	0.481308788
Galactonic acid	0.489950484	0.167937545	0.337505762
L-glutamine	0.937028616	-0.019866715	0.34648663
Ribonic acid	0.90613339	0.025122899	0.100585774
O-phosphoethanolamine	0.074281868	-0.42709352	1.335929959
Trehalose-6-phosphate	0.45211901	0.213609578	0.839517416
Glucose	0.892239037	-0.102773414	0.478740782
Terephthalic acid	0.061558507	0.207594646	0.842212236
Catechin	0.51959385	-0.123799147	0.376866182
Ascorbic acid	0.805255374	-0.188391702	1.018218308
D-fructose	0.656517987	-0.261739257	0.295315005
1-deoxy-1-piperidino-alpha-d-fructose	0.042084206	0.472266986	1.566809882
D-mannose	0.134498307	-0.74549547	1.645109171
3-phosphoglyceric acid	0.187602365	0.375269086	1.051186696
Palatinitol	0.603371844	-0.422272626	0.400672846
Ornithine	0.861661662	0.048582391	0.35291512
Citric acid	0.01292685	0.384792179	1.444683865

Isocitric acid	0.055973459	0.377063693	1.230306223
Citrulline	0.243946616	0.425509219	0.885894115
Guanosine	0.298393464	0.275380418	1.158882613
Epicatechin	0.001670973	0.415889928	1.628975585
(e)-9-tetradecenoic acid	0.755588266	0.185424289	0.958798324
N-acetyl-d-hexosamine	0.713407182	0.11646158	0.70952655
Xylofuranose	0.605800722	-0.133976084	0.166776744
Maltitol	0.762167159	0.057370173	0.315906595
1,5-anhydroglucitol	0.146547142	0.482187021	1.466986106
Triacetin	0.11537094	0.405402627	1.12025299
Myristic acid	0.443001906	0.332892694	1.033600056
Serotonin	0.019068136	-0.527089205	1.615283908
Chlorogenic acid	0.079637295	0.657442125	1.914982061
Quinic acid	0.160671895	0.794255532	1.759489991
Adenine	0.219820799	0.267241763	0.841678885
D-tagatose	0.499610717	0.131895276	0.535126751
D-ribose-5-phosphate	0.090812703	-2.312048563	2.41502094
Delta-tocopherol	0.097216518	0.380618008	1.229574455
Carbamazepine	0.235064084	0.37364576	1.108996799
Beta-gentiobiose	0.392934329	0.201524453	0.387365446
Maltotriose	0.298036088	-0.557770895	0.983445771
Lactitol	0.528473805	-1.037199117	1.301219591
Galactinol	0.408747784	-0.333166795	0.883457225
Phthalimide	0.182706158	0.225362448	0.884045036
L-histidine	0.7218897	-0.174351444	0.621449774
L-lysine	0.652024428	0.112356028	0.188826652
Sorbitol	0.984391075	0.018880216	0.498720018
Galacturonic acid	0.110542519	0.483790785	1.336466763
2'-deoxyguanosine	0.406324358	-0.14509121	0.460855557
Ethyl beta-d-glucopyranoside	0.488101271	-0.341756049	1.148305395
L-tyrosine	0.526252051	0.177027819	0.546264381
Sedoheptulose	0.131572539	0.347696448	0.823634949
Saccharic acid	0.128086766	0.392427897	1.123309174

Lignoceric acid	0.079919457	0.618360748	1.773124057
Lactobionic acid	0.54224324	0.159302616	0.163968185
Melezitose	0.626450444	-0.123905948	0.366337357
Gallic acid	0.000500365	0.495656062	1.834507704
Resveratrol	0.71113155	0.089076207	0.287643179
Isohexonic acid	0.551324153	0.167175509	0.539237382
-[(1-methylethyl)sulfanyl]phenyl}-2-hydroxy]eth	0.351442642	-0.268711154	0.889347864
Cis-gondoic acid	0.722070145	0.076928059	0.437570421
2-amino[1,3]thiazolo[4,5-d]pyrimidine-5,7-diol	0.198418998	1.098300811	1.125103018
1-kestose	0.282387617	-0.257917853	0.772780992
Galactose	0.949875456	0.008772338	0.110355483
Ethyl beta-d-galactofuranoside	0.681089719	-0.103482449	0.243573942
Gluconic acid	0.751294343	0.079434706	0.351841828
Spermine	0.017355905	0.884331997	2.109372822
Acebutolol	0.49980576	0.156355492	0.502814161
Galactaric acid	0.158427276	0.317121154	0.983794725
Erucic acid	0.478222789	0.283804423	0.379035044
Palmitelaidic acid	0.648334502	-0.399904591	0.124380535
Alloxanic acid	0.841803578	-0.025265377	0.181281988
3,4-methylenedioxyamphetamine	0.771197572	0.095204141	0.208016527
Barbital	0.231415443	0.534597181	0.863082275
Montanic acid	0.373608724	0.107499879	0.429627421
Palmitic acid	0.728407103	0.338372244	1.181505236
Indole-3-propionic acid	0.407892661	-1.675196074	1.038467564
Homoserine	0.510490323	0.225607193	0.620385153
P-octopamine	0.165154552	-3.011883464	1.740454152
Erythrotetrofuranose	0.306546009	2.806765162	1.911471405
Myo-inositol	0.126831346	0.22572833	0.881796261
Glycyl tyrosine	0.092435108	0.679543488	1.662482362
-deoxy-1-(4-morpholinyl)-alpha-d-fructopyranos	0.641894616	-0.25591797	0.545853665
Uric acid	0.032976133	0.547913967	1.582419986
Heptadecanoic acid	0.010634987	0.931510482	2.150179749
N-(2-acetamido)iminodiacetic acid	0.853121572	-0.020680938	0.163295514

Alloxanoic acid	0.804766707	-0.048433077	0.219580618
2-aminoethyl methacrylate	0.04981556	0.708784506	1.805417231
Talose	0.674276306	-0.120129357	0.560223232
Caffeic acid	0.000989058	0.616639808	1.995208151
Cetaben	0.241724138	1.160639643	1.643995391
6-hydroxy-alpha-methylnaphthaleneacetic acid	0.320173879	-0.135647058	0.625302421
Octadecanol	0.418929751	0.06926532	0.342363921
Indolelactic acid	0.060990649	0.657788628	1.71554795
Methyl beta-d-glucopyranoside	0.3059813	-0.637629364	0.457094663
5,7-dihydroxy-4'-methoxyisoflavone	0.186640152	0.382348135	0.822621839
1h-indole-3-acetamide	0.812171437	-0.081179032	0.230684346
Cadaverine	0.05116783	0.950297149	2.030127295
L-tryptophan	0.056381973	0.592928231	1.642516208
Stigmasterol	0.411173183	0.192708902	0.791356916
Linoleic acid	0.440032226	0.384799267	0.718310483
Oleic acid	0.854541146	0.059354661	0.158187701
Aprobarbital	0.650039404	-0.24217597	0.665611077
3-aminoisobutanoic acid	0.376764828	0.428546708	0.287779923
4-hydroxyhippuric acid	0.844627235	0.082947344	0.077206534
Bumetanide	0.536082916	-0.340480104	0.023563315
Indoleacetic acid	0.399806733	0.731209784	0.853856997
6,7-dimethyl-4-hydroxy-2-pteridinamine	0.849055265	0.085149066	0.791921904
Mannobiose	0.744933777	0.18576508	0.924860777
3-hydroxyestra-1,3,5(10)-triene-6,17-dione	0.015307258	0.676691848	1.911879038
2-butyl-1-octanol	0.515459192	-0.128702178	0.459562024
Stearic acid	0.121429428	0.341274198	1.130283059
Cholic acid	0.11471107	-0.280827702	0.989975155
6(5h)-benzo[c]phenanthridinone, 11,12-dihydro	0.608065212	-0.254544506	0.305834228
D-fructose-6-phosphate	0.931218366	0.026279752	0.023584925
D-fructose-1,6-bisphosphate	0.258208843	0.287630725	0.779262941
Glucose-6-phosphate	0.770232658	0.089942303	0.210968149
Zeranol	0.714411958	0.05211546	0.115584738
L-fucose	0.578133209	0.255298687	1.189477237

Lithocholic acid	0.905472429	-0.03394189	0.036263282
D-myo-inositol 4-phosphate	0.371609302	0.217481133	0.853838895
N-acetyl-5-hydroxytryptamine	0.956352095	-0.045940886	0.211860483
Triethylene glycol monomethyl ether	0.292760143	0.113183625	0.468468542
Acetamide	0.295123011	0.114438347	0.470040573
Uridine	0.336811607	-0.657867211	0.199609945
Xanthosine	0.548507502	-0.253722129	0.083308033
5-methoxytryptamine	0.13956362	0.469797756	1.028560614
16a-hydroxydehydroisoandrosterone	0.593057962	-0.099977304	0.371336028
Isochlorogenic acid	0.043925765	-0.298743826	1.225939342
Epigallocatechin	0.21043227	0.295869682	0.739940703
2-monostearin	0.005937924	0.494478198	1.739713523
Docosahexaenoic acid	0.432274256	0.305806461	0.714519991
1-monopalmitin	0.244321719	0.133805544	0.578668344
D-xylose	0.125555151	0.388365977	1.482410008
Behenic acid	0.449046335	0.17769172	0.86556222
Pectin	0.09127427	0.551524061	1.70288497
Trisaccharide	0.070920325	0.588248695	1.467775714
Gallocatechin	0.805503569	0.118827835	0.252966215
1-monostearin	0.194409408	0.179352714	0.679107976
Inosine-5'-monophosphate	0.085024241	0.553899782	1.559177811
Gamma-tocopherol	0.55181913	0.224832856	0.010354062
Adenosine-5'-monophosphate	0.04039705	0.487108963	1.460643818
Alpha-tocopherol	0.279066936	0.222754744	0.441950818
Cholesterol	0.157393034	0.180506965	0.716936229
Dihydrocholesterol	0.350956474	-2.261945563	0.760331132
Cholesterone	0.062597754	0.469423622	1.270731368
Zymosterol	0.713908784	0.112038537	0.13806474
Chenodeoxycholic acid	0.018649374	0.614443503	1.788566845
Deoxycholic acid	0.322281818	0.679025037	0.979802574
Beta-sitosterol	0.187612712	0.332203903	1.129071393
Lanosterol	0.364803916	0.33824553	1.414066731

Supplement 2B:This is evidence of group2-group3 volcanic map analysis representing metabolites. Analyzing the differences in metabolite expression in the samples.
