# Supplementary material for: Study on the Metabonomics Mechanism of Mongolian Medical Andai Therapy on Healthy People
Source: Evid Based Complement Alternat Med. 2022 Jun 20;2022:1364408. doi: 10.1155/2022/1364408 (PMC9236767; doi:10.1155/2022/1364408)
Supplement: Supplementary Materials — Supplement 1 is evidence for the principal component analysis (PCA) diagram. Supplement 2A is evidence for (group 1-group 4) female sample comparison volcanic map analysis representing metabolites, as shown in Figure 6. Supplement 2B is evidence for (group 2-group 3) male sample comparison volcanic map analysis representing metabolites, as shown in Figure 7. Supplement2C is evidence for (group 1-group 2 and group 3-group 4) full-sample comparison volcanic map analysis representing metabolites, as shown in Figure 5. Supplement 3A is evidence for (group 1A and group 4A) female sample clustering heat map analysis, as shown in Figure 9. Supplement 3B is evidence for (group 2A and group 3A) male sample clustering heat map analysis, as shown in Figure 10. Supplement 3C is evidence for (group 1A-2A and group 3A-4A) whole-sample clustering heat map analysis， as shown in Figure 8. Supplement 4A is evidence for (group 1A and group 4A) accumulation of metabolic pathways in female samples—Top20, as shown in Figure 13. Supplement 4B is evidence for (group 2 and group 3) accumulation of metabolic pathways in male samples—Top20, as shown in Figure 15. Supplement 4C is evidence for (group 1-2 and group 3-4) enrichment of metabolic pathways in the whole sample—Top20, as shown in Figure 11. Supplement 5A is evidence for (group 1 and group 4) metabolic bubble of female sample, as shown in Figure 14. Supplement 5B is evidence for (group 2 and group 3) metabolic bubble of male sample, as shown in Figure 16. Supplement 5C is evidence for metabolic pathways of (group 1-2 and group 3-4) metabolic bubble of the whole sample, as shown in Figure 12. [file 1364408.f1.zip › 1364408.f1/Supplement 3.pdf]

Supplement 3A

Metabolite name	2	16	38	5	35	40	3	20	39	6	36	41	8	37	42	15	57	62	31	59	64	11	55	60	12	56	61	17	58	63
Sorbitol	0.037765493	0.037765493	0.037765493	0.084483189	0.084483189	0.084483189	0.134114312	0.134114312	0.134114312	0.085268486	0.085268486	0.085268486	0.041795098	0.041795098	0.041795098	0.006706552	0.006706552	0.006706552	0.011755251	0.011755251	0.011755251	0.013178773	0.013178773	0.013178773	0.007817814	0.007817814	0.007817814	0.004127237	0.004127237	0.004127237
Lactitol	0.301927774	0.301927774	0.301927774	0.302578679	0.302578679	0.302578679	0.389302583	0.379302583	0.385302583	0.003298745	0.003298745	0.003298745	0.000133141	0.000133141	0.000133141	0.001141039	0.001141039	0.001141039	0.000846069	0.000846069	0.000846069	0.001414584	0.001414584	0.001414584	0.00167479	0.00167479	0.00167479	0.001238779	0.001238779	0.001238779
Caffeic acid	0.003349568	0.003349568	0.003349568	0.002228413	0.002228413	0.002228413	0.002999058	0.002999058	0.002999058	0.002755413	0.002555413	0.002355413	0.002064876	0.002064876	0.002064876	0.000702622	0.000702622	0.000702622	0.000480078	0.000480078	0.000480078	0.000535728	0.000535728	0.000535728	0.000586475	0.000586475	0.000586475	0.000661232	0.000661232	0.000661232
Palmitic acid	0.001554413	0.001554413	0.001554413	0.001167106	0.001167106	0.001167106	0.006072339	0.006072339	0.006072339	0.002643267	0.002643267	0.002643267	0.002343604	0.002343604	0.002343604	0.000645644	0.000665644	0.000655644	0.000727281	0.000727281	0.000727281	0.000587044	0.000587044	0.000587044	0.0004024	0.0004024	0.0004024	0.000364556	0.000364556	0.000364556
Maltotriose	0.118067816	0.118067816	0.118067816	0.09177256	0.09177256	0.09177256	0.257137147	0.257137147	0.257137147	0.015072156	0.015072156	0.015072156	0.105582936	0.105582936	0.105582936	0.007477366	0.007277366	0.007077366	0.022459225	0.022459225	0.022459225	0.006989328	0.006989328	0.006989328	0.010207227	0.010207227	0.010207227	0.059852029	0.059852029	0.059852029
Gallic acid	0.128266799	0.128266799	0.128266799	0.094309725	0.094309725	0.094309725	0.128211702	0.128211702	0.128211702	0.1023882	0.1023882	0.1023882	0.081714412	0.081714412	0.081714412	0.03704997	0.03704997	0.03704997	0.02808422	0.02808422	0.02808422	0.035051713	0.035051713	0.035051713	0.028951866	0.028951866	0.028951866	0.030223991	0.030223991	0.030223991
Barbital	0.006950239	0.006950239	0.006950239	0.00501282	0.00501282	0.00501282	0.006437585	0.006437585	0.006437585	0.005074131	0.005074131	0.005074131	0.004468195	0.004468195	0.004468195	0.002374015	0.002374015	0.002374015	0.000238631	0.000238631	0.000238631	0.002014567	0.002014567	0.002014567	0.002128729	0.002128729	0.002128729	0.002459698	0.002459698	0.002459698
3-hydroxyestra-1,3,5(10)-triene-6,17-dione	0.000758303	0.000758303	0.000758303	0.000735756	0.000735756	0.000735756	0.000884207	0.000884207	0.000884207	0.001089084	0.001089084	0.001089084	0.00094773	0.00094773	0.00094773	0.000375577	0.000375577	0.000375577	0.000227701	0.000227701	0.000227701	0.00026478	0.00026478	0.00026478	0.000389662	0.000389662	0.000389662	0.000326412	0.000316412	0.000306412
L-tryptophan	0.682704973	0.682704973	0.682704973	0.490064212	0.491064212	0.492064212	0.496259275	0.496259275	0.496259275	0.677167921	0.677167921	0.677167921	0.409197439	0.309197439	0.408197439	0.222608507	0.222608507	0.222608507	0.179111432	0.179111432	0.179111432	0.255663494	0.250663494	0.245663494	0.172433493	0.172433493	0.172433493	0.150248936	0.150248936	0.150248936
Epicatechin	0.003194223	0.003194223	0.003194223	0.00232243	0.002632639	0.002228228	0.00302709	0.00302709	0.00302709	0.002939469	0.002939469	0.002939469	0.00222031	0.00222031	0.00222031	0.001146878	0.001146878	0.001146878	0.000988975	0.000988975	0.000988975	0.001053386	0.001052386	0.001051386	0.001004792	0.001004792	0.001004792	0.000949313	0.000949313	0.000949313
Pyrazin-2-carboxylic acid	0.01233121	0.01233121	0.01233121	0.015500327	0.015500327	0.015500327	0.014885844	0.014885844	0.014885844	0.017984829	0.017984829	0.017984829	0.013930814	0.013930814	0.013930814	0.005737867	0.005737867	0.005737867	0.005019252	0.005019252	0.005019252	0.006175507	0.006175507	0.006175507	0.006464496	0.006364496	0.006264496	0.005017166	0.005017166	0.005017166
Galacturonic acid	0.014005552	0.014005552	0.014005552	0.013375633	0.013375633	0.013375633	0.020854175	0.020854175	0.020854175	0.014254399	0.014254399	0.014254399	0.031632626	0.031632626	0.031632626	0.006163723	0.006163723	0.006163723	0.008637146	0.008537146	0.008607146	0.007701117	0.007701117	0.007701117	0.006061901	0.006061901	0.006061901	0.003765694	0.003765694	0.003765694
Coniferin	0.000742089	0.000742089	0.000742089	0.001051198	0.001051198	0.001051198	0.000653278	0.000653278	0.000653278	0.001008833	0.001008833	0.001008833	0.000601084	0.000601084	0.000601084	0.000233988	0.000233988	0.000233988	0.000127151	0.000127151	0.000127151	0.000406622	0.000416622	0.000426622	0.000482814	0.000482814	0.000482814	0.000281326	0.000281326	0.000281326
Melezitose	0.379247588	0.379247588	0.379247588	0.401046661	0.401046661	0.401046661	0.442105248	0.432105248	0.422105248	0.239462501	0.259462501	0.279462501	0.333429167	0.333029167	0.323429167	0.207834801	0.207834801	0.207834801	0.179628575	0.179628575	0.179628575	0.086590831	0.086500831	0.086510831	0.086426073	0.086426073	0.086426073	0.141314344	0.131314344	0.121314344
Glucose-1-phosphate	0.007578817	0.007578817	0.007578817	0.007222587	0.007222587	0.007222587	0.008750068	0.007750068	0.008350068	0.006538812	0.006538812	0.006538812	0.008330475	0.008030475	0.008310475	0.00364076	0.00364076	0.00364076	0.003134819	0.003134819	0.003134819	0.003275474	0.003175474	0.003205474	0.003130067	0.003130067	0.003130067	0.003134286	0.003034286	0.003334286
Butane-2,3-diol	0.002407819	0.002407819	0.002407819	0.001633627	0.001633627	0.001633627	0.001786379	0.001786379	0.001786379	0.001483204	0.001483204	0.001483204	0.001328179	0.001328179	0.001328179	0.001029387	0.001029387	0.001029387	0.000593685	0.000593685	0.000593685	0.000669099	0.000669099	0.000669099	0.000629858	0.000629858	0.000629858	0.00061302	0.00062302	0.00063302
Cholesterone	0.008652543	0.008652543	0.008652543	0.008113896	0.008113896	0.008113896	0.008938412	0.008938412	0.008938412	0.006386477	0.006386477	0.006386477	0.006439415	0.006439415	0.006439415	0.003866348	0.003866348	0.003866348	0.002664173	0.002664173	0.002664173	0.003049792	0.003049792	0.003049792	0.003744516	0.003744516	0.003744516	0.002939046	0.002939046	0.002939046
Sedoheptulose	0.011789934	0.011789934	0.011789934	0.010446995	0.010446995	0.010446995	0.015304722	0.015304722	0.015304722	0.021163546	0.021163546	0.021163546	0.010323263	0.010323263	0.010323263	0.005312729	0.005312729	0.005312729	0.004623312	0.004623312	0.004623312	0.007810205	0.007710205	0.007819205	0.006002006	0.006002006	0.006002006	0.00331235	0.00331235	0.00331235
Xylonolactone	0.007571481	0.007571481	0.007571481	0.007220207	0.007220207	0.007220207	0.011768398	0.011768398	0.011768398	0.00653446	0.00653446	0.00653446	0.0058824	0.0058824	0.0058824	0.003637809	0.003637809	0.003637809	0.003132242	0.003132242	0.003132242	0.003273968	0.003263968	0.003253968	0.003128754	0.003128754	0.003128754	0.003132028	0.003132028	0.003132028
Saccharic acid	0.00305249	0.00305249	0.00305249	0.002893439	0.002893439	0.002893439	0.004389952	0.004289952	0.004309952	0.003543977	0.003543977	0.003543977	0.005947046	0.005947046	0.005947046	0.001452071	0.001452071	0.001452071	0.002088856	0.002088856	0.002088856	0.001855602	0.001855602	0.001855602	0.001337738	0.001337738	0.001337738	0.001326706	0.001326706	0.001326706
Cis-gondoic acid	0.005097195	0.005097195	0.005097195	0.006073052	0.006073052	0.006073052	0.008986604	0.008886604	0.008906604	0.006258856	0.006258856	0.006258856	0.006338841	0.006338841	0.006338841	0.003376409	0.003376409	0.003376409	0.003194855	0.003194855	0.003194855	0.003694691	0.003694691	0.003694691	0.001022222	0.001022222	0.001022222	0.002474265	0.002474265	0.002474265
Gluconic acid	0.015969714	0.015969714	0.015969714	0.015981129	0.015981129	0.015981129	0.016887991	0.016887991	0.016887991	0.023433797	0.023433797	0.023433797	0.015350963	0.015350963	0.015350963	0.007631061	0.007731061	0.007031061	0.009372	0.009272	0.009072	0.006769116	0.006869116	0.006969116	0.008349711	0.008349711	0.008349711	0.006389591	0.006389591	0.006389591
Ethyl beta-d-galactofuranoside	0.018809973	0.018809973	0.018809973	0.018629818	0.018629818	0.018629818	0.031651722	0.031651722	0.031651722	0.019055472	0.019055472	0.019055472	0.017502491	0.017502491	0.017502491	0.010905826	0.011905826	0.010805826	0.011200785	0.011200785	0.011200785	0.007699192	0.007799192	0.007999192	0.008218415	0.008118415	0.008018415	0.007634868	0.007634868	0.007634868
D-ribose	0.001320716	0.001320716	0.001320716	0.000901324	0.000901324	0.000901324	0.001448569	0.001448569	0.001448569	0.000865702	0.000865702	0.000865702	0.000901943	0.000901943	0.000901943	0.000531202	0.000581202	0.000631202	0.000672628	0.000672628	0.000672628	0.0006897	0.0006857	0.0006827	0.000243142	0.000233142	0.000223142	0.000136452	0.000136452	0.000136452
Talose	0.005866428	0.005866428	0.005866428	0.004229089	0.004229089	0.004229089	0.007914587	0.007814587	0.007714587	0.003546147	0.003546147	0.003546147	0.003764216	0.003564216	0.003464216	0.002707483	0.002707483	0.002707483	0.002847263	0.002847263	0.002847263	0.001609007	0.001609007	0.001609007	0.001284914	0.001284914	0.001284914	0.002104667	0.002104667	0.002104667
Isochlorogenic acid	0.000942084	0.000942084	0.000942084	0.000826394	0.000826394	0.000826394	0.001320187	0.001330187	0.001350187	0.001416258	0.001416258	0.001416258	0.000885991	0.000895991	0.000880991	0.002757014	0.002757014	0.002757014	0.001776371	0.001776371	0.001776371	0.002679143	0.002679143	0.002679143	0.002472045	0.002472045	0.002472045	0.00231903	0.00221903	0.00239903
Taurine	0.034671427	0.034671427	0.034671427	0.019118146	0.019118146	0.019118146	0.019983743	0.019983743	0.019983743	0.029187024	0.029187024	0.029187024	0.015369099	0.015369099	0.015369099	0.010606478	0.010706478	0.010806478	0.009528553	0.009028553	0.009128553	0.006924336	0.006924336	0.006924336	0.012985942	0.012985942	0.012985942	0.009440577	0.009440577	0.009440577
3-hydroxybenzoic acid	0.001803081	0.001803081	0.001803081	0.001102922	0.001102922	0.001102922	0.001385053	0.001385053	0.001385053	0.001485677	0.001485677	0.001485677	0.001007735	0.001007735	0.001007735	0.000758327	0.000748327	0.000738327	0.000511452	0.000521452	0.000531452	0.000613163	0.000613163	0.000613163	0.000612115	0.000612115	0.000612115	0.00057159	0.00057159	0.00057159
Chenodeoxycholic acid	0.016091761	0.016091761	0.016091761	0.007867789	0.007867789	0.007867789	0.012583888	0.012583888	0.012583888	0.00968221	0.00968221	0.00968221	0.016529294	0.016529294	0.016529294	0.002983796	0.002983796	0.002983796	0.004091754	0.004091754	0.004091754	0.008031936	0.008031936	0.008031936	0.008325167	0.008325167	0.008325167	0.003917891	0.003817891	0.003717891
Adenine	0.002786405	0.002686405	0.002706405	0.002262205	0.002262205	0.002262205	0.002573575	0.002573575	0.002573575	0.002472459	0.002472459	0.002472459	0.001412156	0.001412156	0.001412156	0.001211755	0.001211755	0.001211755	0.000860003	0.000860003	0.000860003	0.001213765	0.001213765	0.001213765	0.000998589	0.000998589	0.000998589	0.001063518	0.001063518	0.001063518
Beta-gentiobiose	0.185201212	0.175201212	0.180201212	0.121526365	0.121526365	0.121526365	0.100469541	0.100469541	0.100469541	0.084181026	0.084181026	0.084181026	0.053747833	0.053747833	0.053747833	0.017644293	0.017644293	0.017644293	0.059606187	0.058606187	0.057606187	0.018237809	0.018237809	0.018237809	0.037688515	0.037688515	0.037688515	0.092998466	0.092998466	0.092998466
Alloxanoic acid	0.003435051	0.003435051	0.003435051	0.004036504	0.004036504	0.004036504	0.005723117	0.005723117	0.005723117	0.009089692	0.009089692	0.009089692	0.007344542	0.007344542	0.007344542	0.011456212	0.011456212	0.011456212	0.011385616	0.011375616	0.011395616	0.011884327	0.011884327	0.011884327	0.011400821	0.011400821	0.011400821	0.018538606	0.018538606	0.018538606
Alpha-tocopherol	0.225166189	0.224166189	0.223166189	0.320264095	0.320264095	0.320264095	0.247234417	0.257234417	0.247934417	0.339385254	0.329385254	0.330385254	0.223490043	0.223490043	0.223490043	0.120212733	0.119212733	0.118212733	0.091923864	0.091923864	0.091923864	0.118636418	0.118636418	0.118636418	0.189141054	0.185141054	0.180141054	0.121078975	0.121078975	0.121078975
Digalacturonic acid	0.001276368	0.001276368	0.001276368	0.001017741	0.001017741	0.001017741	0.001412345	0.001412345	0.001412345	0.002405409	0.002505409	0.002605409	0.000809215	0.000809215	0.000809215	0.00045915	0.00045915	0.00045915	0.000456638	0.000456638	0.000456638	0.000869528	0.000859528	0.000849528	0.000739007	0.000729007	0.000719007	0.000385044	0.000385044	0.000385044
D-myo-inositol 4-phosphate	0.022966214	0.022966214	0.022966214	0.025216951	0.025216951	0.025216951	0.033647365	0.033647365	0.033647365	0.031863026	0.031863026	0.031863026	0.021163552	0.021163552	0.021163552	0.012362645	0.012362645	0.012362645	0.013392387	0.013392387	0.013392387	0.014433211	0.014833211	0.014463211	0.015311365	0.015011365	0.015211365	0.009683535	0.009683535	0.009683535
L-arabitol	0.011104407	0.011104407	0.011104407	0.012582463	0.012582463	0.012582463	0.019140988	0.019140988	0.019140988	0.017144979	0.017144979	0.017144979	0.011908427	0.011908427	0.011908427	0.010126091	0.010126091	0.010126091	0.007264744	0.007264744	0.007264744	0.006182183	0.006182183	0.006182183	0.006255119	0.006255119	0.006255119	0.0042878	0.0042878	0.0042878
Dihydrocholesterol	0.007938094	0.007938094	0.007938094	0.00799798	0.00799798	0.00799798	0.010671558	0.010671558	0.010671558	0.008011406	0.008011406	0.008011406	0.007777884	0.007777884	0.007777884	0.00500527	0.00500527	0.00500527	0.003419934	0.003419934	0.003419934	0.003898783	0.003898783	0.003898783	0.004578685	0.004578685	0.004578685	0.004624705	0.004624705	0.004624705
Lactobionic acid	0.014902096	0.014802096	0.014702096	0.014203447	0.014203447	0.014203447	0.013668129	0.013668129	0.013668129	0.017288777	0.017188777	0.017088777	0.014746571	0.014746571	0.014746571	0.011401426	0.011401426	0.011401426	0.00670472	0.00670472	0.00670472	0.004214449	0.004514449	0.004714449	0.006192274	0.006192274	0.006192274	0.008463499	0.008463499	0.008463499
1-deoxy-1-piperidino-alpha-d-fructose	0.000899512	0.000899512	0.000899512	0.001140239	0.001140239	0.001140239	0.000852955	0.000852955	0.000852955	0.000789955	0.000799955	0.000780955	0.000976576	0.000976576	0.000976576	0.000420324	0.000420324	0.000420324	0.000484562	0.000484562	0.000484562	0.000550193	0.000580193	0.000530193	0.000474528	0.000474528	0.000474528	0.000422679	0.000422679	0.000422679
L-methionine	0.072903131	0.070903131	0.071903131	0.055413839	0.055413839	0.055413839	0.049227684	0.049227684	0.049227684	0.08982697	0.08982697	0.08982697	0.056167253	0.056167253	0.056167253	0.033479451	0.033479451	0.033479451	0.040791728	0.040791728	0.040791728	0.033397425	0.033397425	0.033397425	0.025017036	0.025017036	0.025017036	0.024671915	0.024671915	0.024671915
Guanosine	0.001468634	0.001468634	0.001468634	0.000834961	0.000834961	0.000834961	0.001401833	0.001401833	0.001401833	0.002817756	0.002817756	0.002817756	0.001202642	0.001202642	0.001202642	0.000799831	0.000799831	0.000799831	0.00066774	0.00066774	0.00066774	0.000695308	0.000695308	0.000695308	0.000688219	0.000688219	0.000688219	0.00047437	0.00047437	0.00047437
Cetaben	0.016893105	0.016893105	0.016893105	0.021965478	0.021965478	0.021965478	0.018828526	0.018828526	0.018828526	0.031993411	0.031993411	0.031993411	0.020609556	0.020609556	0.020609556	0.0051802	0.0051802	0.0051802	0.010327156	0.010527156	0.010927156	0.015645087	0.015645087	0.015645087	0.010504738	0.010504738	0.010504738	0.01172714	0.01172714	0.01172714
D-arabinose	0.001255741	0.001255741	0.001255741	0.00085225	0.00085225	0.00085225	0.001448569	0.001448569	0.001448569	0.000865702	0.000865702	0.000865702	0.000866166	0.000866166	0.000866166	0.000522224	0.000522224	0.000522224	0.000640764	0.000648764	0.000642764	0.000659465	0.000659465	0.000659465	0.000449134	0.000449134	0.000449134	0.000376603	0.000376603	0.000376603
L-glutamine dehydrated	0.019898975	0.019898975	0.019898975	0.015974422	0.015974422	0.015974422	0.013177109	0.013177109	0.013177109	0.017899902	0.017899902	0.017899902	0.014372169	0.014372169	0.014372169	0.012460102	0.012460102	0.012460102	0.009562272	0.009562272	0.009562272	0.006205394	0.006105394	0.006005394	0.006074009	0.006074009	0.006074009	0.007822782	0.007822782	0.007822782
Glycine	0.903589216	0.893589216	0.903089216	0.388544009	0.398544009	0.399544009	0.478993227	0.498993227	0.508993227	0.64370563	0.64370563	0.64370563	0.665643752	0.665643752	0.665643752	0.500253712	0.500253712	0.500253712	0.236881063	0.236881063	0.236881063	0.235017191	0.245017191	0.255017191	0.279066783	0.279066783	0.279066783	0.237750229	0.247750229	0.257750229
Cholesterol	8.670486795	8.570486795	8.470486795	8.081183705	8.181183705	8.091183705	9.865619045	9.565619045	9.365619045	8.811885489	8.711885489	8.611885489	7.828517734	7.828517734	7.828517734	4.696729213	4.706729213	4.699729213	4.474342129	4.484342129	4.494342129	5.040815843	5.040815843	5.040815843	5.193812143	5.093812143	5.103812143	4.57858237	4.77858237	4.67858237
Triacetin	0.181443886	0.171443886	0.151443886	0.103544556	0.103044556	0.103544555	0.08914283	0.08914283	0.08914283	0.155966879	0.155966879	0.155966879	0.081888373	0.081888373	0.081888373	0.061914775	0.062914775	0.061994775	0.063931272	0.064931272	0.063991272	0.066628954	0.066628954	0.066628954	0.050489882	0.050489882	0.050489882	0.050996923	0.050996923	0.050996923
Methanephosphonothioic acid	0.016340839	0.016340839	0.016340839	0.022940477	0.020147492	0.019640309	0.020147492	0.020147492	0.020147492	0.026616045	0.026616045	0.026616045	0.019640309	0.019640309	0.019640309	0.012246939	0.012246939	0.012246939	0.009869442	0.009969442	0.009899442	0.010885292	0.010885292	0.010885292	0.013453194	0.013453194	0.013453194	0.010607034	0.010607034	0.010607034
Galactaric acid	0.002809342	0.002809342	0.002809342	0.002096653	0.002096653	0.002096653	0.003051568	0.003051568	0.003051568	0.003482502	0.003482502	0.003482502	0.002330254	0.002330254	0.002330254	0.001404801	0.001404801	0.001404801	0.001438479	0.001438479	0.001438479	0.001390459	0.001390459	0.001390459	0.002002917	0.002002917	0.002002917	0.001001815	0.001001815	0.001001815
Kynurenic acid	0.017124066	0.017124066	0.017124066	0.016526579	0.016526579	0.016526579	0.020183683	0.020183683	0.020183683	0.0205102	0.0205102	0.0205102	0.014727722	0.014727722	0.014727722	0.008199212	0.008199212	0.008199212	0.010413941	0.010413941	0.010413941	0.011312693	0.011312693	0.011312693	0.011013284	0.011013284	0.011013284	0.008116886	0.008116886	0.008116886
Galactitol	0.0037426	0.0037426	0.0037426	0.003289182	0.003289182	0.003289182	0.004177978	0.004177978	0.004177978	0.003163194	0.003163194	0.003163194	0.002136093	0.002136093	0.002136093	0.001960676	0.001960676	0.001960676	0.001939723	0.001939723	0.001939723	0.00218651	0.00218651	0.00218651	0.001050113	0.001050113	0.001050113	0.001526886	0.001526886	0.001526886
Carbamazepine	0.204445914	0.204445914	0.204445914	0.123198086	0.123198086	0.123198086	0.096565783	0.093565783	0.092565783	0.0827252	0.0827252	0.0827252	0.079526406	0.078526406	0.077526406	0.053759459	0.053759459	0.053759459	0.048259719	0.048259719	0.048259719	0.054491838	0.054491838	0.054491838	0.070243262	0.069243262	0.071243262	0.055203463	0.055203463	0.055203463
Alizarin	0.002599077	0.002599077	0.002599077	0.002017984	0.002017984	0.002017984	0.002214627	0.002214627	0.002214627	0.003623291	0.003623291	0.003623291	0.002576623	0.002476623	0.002376623	0.001127556	0.001127556	0.001127556	0.00220841	0.00220841	0.00220841	0.001413509	0.001413509	0.001413509	0.001233382	0.001233382	0.001233382	0.000966522	0.000966522	0.000966522
Naproxen	0.002437255	0.002437255	0.002437255	0.002407286	0.002407286	0.002407286	0.003386956	0.003386956	0.003386956	0.003905374	0.003905374	0.003905374	0.002437254	0.002437254	0.002437254	0.001628238	0.001628238	0.001628238	0.002684548	0.002684548	0.002684548	0.001667562	0.001667562	0.001667562	0.000663732	0.000663732	0.000663732	0.00111388	0.00111388	0.00111388
(s)-3,4-dihydroxybutyric acid	0.001273034	0.001273034	0.001273034	0.001348791	0.001348791	0.001348791	0.001285861	0.001285861	0.001285861	0.001575095	0.001575095	0.001575095	0.001286889	0.001286889	0.001286889	0.000792194	0.000792194	0.000792194	0.000957381	0.000957381	0.000957381	0.000858537	0.000858537	0.000858537	0.000719688	0.000719688	0.000719688	0.000654572	0.000654572	0.000654572
Lanosterol	0.008529626	0.008529626	0.008529626	0.011302556	0.011302556	0.011302556	0.00836661	0.00836661	0.00836661	0.012706599	0.012706599	0.012706599	0.00489927	0.00489927	0.00489927	0.007898221	0.007898221	0.007898221	0.005515764	0.005515764	0.005515764	0.001520995	0.001520995	0.001520995	0.006135896	0.006135896	0.006135896	0.002142086	0.002142086	0.002142086
D-fucose	0.001352256	0.001352256	0.001352256	0.001893104	0.001893104	0.001893104	0.001750464	0.001750464	0.001750464	0.00170036	0.00170036	0.00170036	0.001399145	0.001399145	0.001399145	0.000812439	0.000852439	0.000842439	0.000876585	0.000876585	0.000876585	0.001415791	0.001415791	0.001415791	0.001262737	0.001262737	0.001262737	6.22413E-05	6.22413E-05	6.22413E-05
16a-hydroxydehydroisoandrosterone	0.005052815	0.005052815	0.005052815	0.005235986	0.005235986	0.005235986	0.006532337	0.006532337	0.006532337	0.00946494	0.00946494	0.00946494	0.007069572	0.007069572	0.007069572	0.012588831	0.012508831	0.011588831	0.009591212	0.009491212	0.009291212	0.011699081	0.011699081	0.011699081	0.011852971	0.011852971	0.011852971	0.012425818	0.012425818	0.012425818
Boric acid	0.083933049	0.083933049	0.083933049	0.067123331	0.077123331	0.080123331	0.086839476	0.086839476	0.086839476	0.102599906	0.102599906	0.102599906	0.069061668	0.069061668	0.069061668	0.037010574	0.037010574	0.037010574	0.05762607	0.05762607	0.05762607	0.045713276	0.045713276	0.045713276	0.052230856	0.052230856	0.052230856	0.043683686	0.043683686	0.043683686
L-isoleucine	0.272235861	0.273235861	0.270235861	0.287582997	0.277582997	0.280582997	0.276819997	0.296819997	0.279819997	0.398261297	0.388261297	0.399261297	0.250355805	0.250355805	0.250355805	0.161792624	0.161792624	0.161792624	0.192959021	0.192959021	0.192959021	0.202973307	0.192973307	0.202073307	0.150139468	0.155139468	0.154139468	0.159694438	0.158694438	0.157694438
Erythronic acid	0.018442482	0.017442482	0.018042482	0.013842571	0.013842571	0.013842571	0.015218824	0.015218824	0.015218824	0.019285025	0.019285025	0.019285025	0.00776671	0.00776671	0.00776671	0.038567206	0.038567206	0.038567206	0.03423265	0.03423265	0.03423265	0.023571115	0.022571115	0.021571115	0.014734784	0.014834784	0.014934784	0.037385289	0.037385289	0.037385289
Pyrophosphate	0.014435561	0.014435561	0.014435561	0.020687377	0.020687377	0.020687377	0.018267343	0.018267343	0.018267343	0.030660655	0.030660655	0.030660655	0.028702903	0.028702903	0.028702903	0.036178616	0.036178616	0.036178616	0.033473155	0.033473155	0.033473155	0.037805689	0.037805689	0.037805689	0.04804624	0.04804624	0.04804624	0.038178594	0.038178594	0.038178594
L-histidine	0.032500678	0.032500678	0.032500678	0.021011379	0.021011379	0.021011379	0.023791114	0.023791114	0.023791114	0.036201372	0.036201372	0.036201372	0.017653302	0.017953302	0.018653302	0.024209454	0.024209454	0.024209454	0.014224717	0.014224717	0.014224717	0.008774637	0.008774637	0.008774637	0.008492218	0.008492218	0.008492218	0.016367928	0.016367928	0.016367928
Sulfuric acid	0.001667601	0.001607601	0.001660601	0.001420018	0.001420018	0.001420018	0.001769373	0.001769373	0.001769373	0.001806287	0.001806287	0.001806287	0.001384981	0.001384981	0.001384981	0.000825894	0.000825894	0.000825894	0.000924903	0.000924903	0.000924903	0.001160111	0.001160111	0.001160111	0.001122604	0.001122604	0.001122604	0.000851058	0.000851058	0.000851058
1,2,4-benzenetriol	0.01923027	0.01903027	0.0192302	0.017950915	0.017950915	0.017950915	0.021034213	0.021034213	0.021034213	0.021432996	0.021432996	0.021432996	0.018323628	0.018323628	0.018323628	0.011331721	0.011331721	0.011331721	0.011354064	0.011354064	0.011354064	0.014065291	0.014065291	0.014065291	0.013232914	0.013232914	0.013232914	0.011156825	0.011156825	0.011156825
L-tyrosine	1.495708796	1.455708796	1.435708796	0.888071126	0.898071126	0.899071126	1.139724146	1.239724146	1.159724146	1.893514284	1.893514284	1.893514284	0.911549986	0.901549986	0.911049986	0.700062688	0.750062688	0.780062688	0.87519186	0.87519186	0.87519186	0.811691822	0.811691822	0.811691822	0.401528573	0.391528573	0.411528573	0.658910411	0.658910411	0.658910411
Oleamide	0.001073583	0.001073583	0.001073583	0.00118723	0.00118723	0.00118723	0.001048374	0.001048374	0.001048374	0.000977055	0.000977055	0.000977055	0.001010022	0.001010022	0.001010022	0.000640201	0.000650201	0.000649201	0.000656691	0.000656691	0.000656691	0.000765101	0.000765101	0.000765101	0.000665452	0.000765452	0.000675452	0.000592748	0.000592748	0.000592748
N-acetylglutamate	0.001449341	0.001449341	0.001449341	0.000931284	0.000931284	0.000931284	0.00099428	0.00099428	0.00099428	0.001749976	0.001749976	0.001749976	0.001514795	0.001514795	0.001514795	0.001153495	0.001153495	0.001153495	0.000669906	0.000669906	0.000669906	0.00047391	0.00047391	0.00047391	0.00039638	0.00039638	0.00039638	0.00103891	0.00103891	0.00103891
5-hydroxy-3-indoleacetic acid	0.000667059	0.000667059	0.000667059	0.000837876	0.000837876	0.000837876	0.001178405	0.001178405	0.001178405	0.001247053	0.001247053	0.001247053	0.000713385	0.000713385	0.000713385	0.000613188	0.000613188	0.000613188	0.000552268	0.000552268	0.000552268	0.000519364	0.000519364	0.000519364	0.000577021	0.000577021	0.000577021	0.00034578	0.00034578	0.00034578
Dehydroascorbic acid	0.003264712	0.003264712	0.003264712	0.002762705	0.002762705	0.002762705	0.002754351	0.002754351	0.002754351	0.004681596	0.004681596	0.004681596	0.002954982	0.002954982	0.002954982	0.001875193	0.001875193	0.001875193	0.002207921	0.002207921	0.002207921	0.002223898	0.002223898	0.002223898	0.001852522	0.001852522	0.001852522	0.001519668	0.001519668	0.001519668
Erythritol	0.012922248	0.012922248	0.012922248	0.015295111	0.015295111	0.015295111	0.017458	0.017458	0.017458	0.019050301	0.019050301	0.019050301	0.009289514	0.009289514	0.009289514	0.011475828	0.011475828	0.011475828	0.008763642	0.008763642	0.008763642	0.008712009	0.008712009	0.008712009	0.007377869	0.007277869	0.007177869	0.005827256	0.005827256	0.005827256
Maleimide	0.005162139	0.005162139	0.005162139	0.003697715	0.003697715	0.003697715	0.003903494	0.003903494	0.003903494	0.004410375	0.004410375	0.004410375	0.004013557	0.004013557	0.004013557	0.003426	0.003426	0.003426	0.002529537	0.002529537	0.002529537	0.00192201	0.00192201	0.00192201	0.002480951	0.002480951	0.002480951	0.002664756	0.002664756	0.002664756
Serine	0.464764905	0.454764905	0.434764905	0.34558967	0.35558967	0.34598967	0.374432092	0.374432092	0.374432092	0.544496978	0.514496978	0.504496978	0.340405571	0.340405571	0.340405571	0.371593876	0.371593876	0.371593876	0.194865614	0.194865614	0.194865614	0.243672278	0.243672278	0.243672278	0.194101529	0.199101529	0.194901529	0.236756614	0.226756614	0.216756614
2-ketoisovaleric acid	0.004146636	0.004146636	0.004146636	0.007630099	0.007930099	0.008030099	0.008299372	0.008299372	0.008299372	0.006167563	0.006067563	0.005967563	0.005978479	0.005978479	0.005978479	0.00332463	0.00332463	0.00332463	0.005188063	0.005188063	0.005188063	0.003671133	0.003671133	0.003671133	0.003263482	0.003273482	0.003283482	0.003351278	0.003351278	0.003351278
2-monostearin	0.004386468	0.004386468	0.004386468	0.003634156	0.003634156	0.003634156	0.004625363	0.004625363	0.004625363	0.003260083	0.003260083	0.003260083	0.002834699	0.002834699	0.002834699	0.002602501	0.002602501	0.002602501	0.001856503	0.001856503	0.001856503	0.002488778	0.002388778	0.002408778	0.002418366	0.002418366	0.002418366	0.002046093	0.002046093	0.002046093
Quinic acid	0.003886949	0.003886949	0.003886949	0.004024056	0.004024056	0.004024056	0.003656032	0.003656032	0.003656032	0.003033059	0.003033059	0.003033059	0.001600517	0.001600517	0.001600517	0.001285721	0.001285721	0.001285721	0.001201202	0.001201202	0.001201202	0.003011656	0.003011656	0.003011656	0.001026116	0.001026116	0.001026116	0.002477161	0.002477161	0.002477161
Leucine	0.589509097	0.579509097	0.599509097	0.605148841	0.605148841	0.605148841	0.509768518	0.589768518	0.529768518	0.791547813	0.761547813	0.751547813	0.46195127	0.46195127	0.46195127	0.37005318	0.38005318	0.39005318	0.449941818	0.449941818	0.449941818	0.391453656	0.381453656	0.371453656	0.280884859	0.280884859	0.280884859	0.300835915	0.300535915	0.300035915
Benzoic acid	0.001462889	0.001462889	0.001462889	0.001517791	0.001517791	0.001517791	0.00101602	0.00101602	0.00101602	0.001418779	0.001318779	0.001218779	0.001065297	0.001065297	0.001065297	0.000606076	0.000616076	0.000609076	0.000617997	0.000617997	0.000617997	0.001074831	0.001074831	0.001074831	0.000860827	0.000860827	0.000860827	0.000752164	0.000752164	0.000752164
Stigmasterol	0.072765702	0.082765702	0.092765702	0.097784456	0.097784456	0.097784456	0.129455325	0.129455325	0.129455325	0.079344253	0.079344253	0.079344253	0.074365662	0.072365662	0.070365662	0.063751206	0.063751206	0.063751206	0.070193811	0.070193811	0.070193811	0.041174488	0.041174488	0.041174488	0.058405465	0.058405465	0.058405465	0.034770188	0.034770188	0.034770188
thoxy-5-prop-2-en-1-yl-2-hydroxy-n-2-hydroxyethylbenza	0.005473801	0.005473801	0.005473801	0.004412675	0.004412675	0.004412675	0.006250693	0.006250693	0.006250693	0.004905643	0.004905643	0.004905643	0.004752425	0.004952425	0.004752425	0.004904984	0.004904984	0.004904984	0.003654396	0.003654396	0.003654396	0.002893294	0.002893294	0.002893294	0.002319963	0.002319963	0.002319963	0.002350222	0.002350222	0.002350222
Phytosphingosine	0.001321934	0.001321934	0.001321934	0.000778226	0.000778226	0.000778226	0.000864277	0.000864277	0.000864277	0.001122071	0.001122071	0.001122071	0.000801971	0.000791971	0.000801071	0.000780528	0.000780528	0.000780528	0.000557388	0.000557388	0.000557388	0.000571254	0.000571254	0.000571254	0.00059388	0.00058388	0.00059988	0.000498972	0.000498972	0.000498972
Udp-n-acetylglucosamine	0.001321934	0.001321934	0.001321934	0.000774448	0.000774448	0.000774448	0.000793426	0.000793426	0.000793426	0.001031363	0.001031363	0.001031363	0.000792583	0.000792583	0.000792583	0.000780528	0.000790528	0.000770528	0.000557388	0.000557388	0.000557388	0.000466967	0.000466967	0.000466967	0.00059388	0.00059388	0.00059388	0.000478942	0.000478942	0.000478942
Malonic acid	0.000419397	0.000419397	0.000419397	0.000388342	0.000388342	0.000388342	0.000434091	0.000434091	0.000434091	0.000461288	0.000461288	0.000461288	0.000351523	0.000351523	0.000351523	0.000263578	0.000263578	0.000263578	0.000251147	0.000251147	0.000251147	0.000300797	0.000300797	0.000300797	0.000313557	0.000313557	0.000313557	0.00023074	0.00023074	0.00023074
Cyclohexylamine	0.000914509	0.000914509	0.000914509	0.000994262	0.000994262	0.000994262	0.000849857	0.000849857	0.000849857	0.001200907	0.001200907	0.001200907	0.000968946	0.000968946	0.000968946	0.001607756	0.001607756	0.001607756	0.001399786	0.001399786	0.001399786	0.001316238	0.001316238	0.001316238	0.001448633	0.001448633	0.001448633	0.001627142	0.001627142	0.001627142
L-phenylalanine	0.110937189	0.111937189	0.110837189	0.115045864	0.115045864	0.115045864	0.104143197	0.104143197	0.104143197	0.162490314	0.152490314	0.142490314	0.108845684	0.108845684	0.108845684	0.094913432	0.094913432	0.094913432	0.105480955	0.105480955	0.105480955	0.068718685	0.066718685	0.065718685	0.052284711	0.055284711	0.056284711	0.056586179	0.056586179	0.056586179
Terephthalic acid	0.00801414	0.00801414	0.00801414	0.006641584	0.006641584	0.006641584	0.008422464	0.008422464	0.008422464	0.007892786	0.007592786	0.007392786	0.006752031	0.006752031	0.006752031	0.005267497	0.005267497	0.005267497	0.00460268	0.00460268	0.00460268	0.005664003	0.005564003	0.005464003	0.005056738	0.004956738	0.005006738	0.004939155	0.004939155	0.004939155
L-glutamine	1.091623169	1.081623169	1.071623169	0.973074177	0.973074177	0.973074177	0.77335272	0.87335272	0.79335272	1.150128736	1.140128736	1.130128736	1.094741839	1.094741839	1.094741839	0.985914076	0.985914076	0.985914076	0.795572443	0.785572443	0.775572443	0.427922273	0.427922273	0.427922273	0.378958328	0.378958328	0.378958328	0.625538702	0.525538702	0.605538702
Phenol	0.000722696	0.000722696	0.000722696	0.000749702	0.000749702	0.000749702	0.001109229	0.001109229	0.001109229	0.000793923	0.000793923	0.000793923	0.000902686	0.000902686	0.000902686	0.00053085	0.00053085	0.00053085	0.000491918	0.000481918	0.000492918	0.000435793	0.000435793	0.000435793	0.000762379	0.000762379	0.000762379	0.000527939	0.000527939	0.000527939
Methylamine	1.724209652	1.774209652	1.754209652	1.761881697	1.761881697	1.761881697	1.840520536	1.845520536	1.849520536	2.042828273	2.032828273	2.022828273	1.933190411	1.933190411	1.933190411	1.434284446	1.334284446	1.404284446	1.154435958	1.150435958	1.153435958	1.420472608	1.410472608	1.400472608	1.203462477	1.203462477	1.203462477	1.188179925	1.198179925	1.178179925
L-cysteine	0.029853017	0.028853017	0.029953017	0.028359947	0.028359947	0.028359947	0.019423013	0.019423013	0.019423013	0.034702807	0.033702807	0.031702807	0.024650444	0.024650444	0.024650444	0.0269214	0.0279214	0.0259214	0.017863572	0.017763572	0.017963572	0.011280749	0.012280749	0.011580749	0.012957391	0.013957391	0.015957391	0.016879023	0.015879023	0.016079023
L-proline	0.67399967	0.68399967	0.69399967	0.767135544	0.767135544	0.767135544	0.712076873	0.712076873	0.712076873	0.794042425	0.784042425	0.754042425	0.514017849	0.514017849	0.514017849	0.615021301	0.610021301	0.612021301	0.567949084	0.557949084	0.560949084	0.373070563	0.373070563	0.373070563	0.367750477	0.360750477	0.365750477	0.288684565	0.288684565	0.288684565
Indolelactic acid	0.035427207	0.035427207	0.035427207	0.028053632	0.028053632	0.028053632	0.050239465	0.050239465	0.050239465	0.044464509	0.044464509	0.044464509	0.020289922	0.020289922	0.020289922	0.02023423	0.02023423	0.02023423	0.019511561	0.020511561	0.021511561	0.026451062	0.026451062	0.026451062	0.024346626	0.024346626	0.024346626	0.014383711	0.014383711	0.014383711
Tromethamine	0.00081277	0.00081277	0.00081277	0.000501262	0.000501262	0.000501262	0.000919973	0.000919973	0.000919973	0.000619294	0.000619294	0.000619294	0.000468905	0.000468905	0.000468905	0.000339459	0.000339459	0.000339459	0.00038469	0.00038469	0.00038469	0.000380376	0.000380376	0.000380376	0.000505524	0.000505524	0.000505524	0.000431546	0.000431546	0.000431546
2-ketoisocaproic acid	0.010128907	0.010128907	0.010128907	0.014693047	0.014693047	0.014693047	0.014988302	0.014988302	0.014988302	0.016931026	0.016931026	0.016931026	0.016228551	0.016228551	0.016228551	0.007305789	0.007305789	0.007305789	0.011562484	0.011562484	0.011562484	0.010423807	0.010423807	0.010423807	0.008610918	0.008610918	0.008610918	0.009663923	0.009663923	0.009663923
Pentadecanol	0.000970072	0.000970072	0.000970072	0.000662435	0.000662435	0.000662435	0.000718666	0.000718666	0.000718666	0.000804095	0.000804095	0.000804095	0.000647423	0.000647423	0.000647423	0.000803226	0.000803226	0.000803226	0.000429071	0.000429071	0.000429071	0.000234351	0.000234351	0.000234351	0.000421488	0.000421488	0.000421488	0.000555836	0.000555836	0.000555836
2,6-bis(tert-butyl)phenol	0.013734769	0.013534769	0.013634769	0.013255306	0.013255306	0.013255306	0.012830777	0.012830777	0.012830777	0.016487843	0.016487843	0.016487843	0.011194581	0.011194581	0.011194581	0.010345134	0.010345134	0.010345134	0.008755749	0.008755749	0.008755749	0.008975194	0.008975194	0.008975194	0.009076868	0.009176868	0.009276868	0.009235563	0.009235563	0.009235563
L-threonine	0.222421022	0.222421022	0.222421022	0.132167555	0.132167555	0.132167555	0.165930276	0.165930276	0.165930276	0.248742019	0.238742019	0.208742019	0.180465622	0.180465622	0.180465622	0.130858176	0.135858176	0.136858176	0.141788626	0.141788626	0.141788626	0.126102028	0.126102028	0.126102028	0.108459463	0.105459463	0.106459463	0.103834824	0.103834824	0.103834824
Cellobiose	0.000487704	0.000487704	0.000487704	0.000426185	0.000426185	0.000426185	0.00047063	0.00047063	0.00047063	0.000345001	0.000348001	0.000340001	0.000358503	0.000358503	0.000358503	0.000299964	0.000299964	0.000299964	0.000257131	0.000257131	0.000257131	0.000288992	0.000288992	0.000288992	0.000298273	0.000298273	0.000298273	0.000289404	0.000289404	0.000289404
N-(2-acetamido)iminodiacetic acid	0.073587937	0.073587937	0.073587937	0.078837509	0.078837509	0.078837509	0.089611832	0.089611832	0.089611832	0.114212109	0.114212109	0.114212109	0.093910982	0.092910982	0.091910982	0.135489378	0.135489378	0.135489378	0.120509074	0.120509074	0.120509074	0.135435077	0.135435077	0.135435077	0.129782857	0.129782857	0.129782857	0.126532151	0.126532151	0.126532151
Uracil	0.003022433	0.003022433	0.003022433	0.002266498	0.002266498	0.002266498	0.00317209	0.00317209	0.00317209	0.002748366	0.002748366	0.002748366	0.001932113	0.001932113	0.001932113	0.001962695	0.001962695	0.001962695	0.001226229	0.001226229	0.001226229	0.001453153	0.001453153	0.001453153	0.001922333	0.001922333	0.001922333	0.002103794	0.002103794	0.002103794
Gamma-aminobutyric acid	0.085215124	0.085215124	0.085215124	0.072210019	0.072210019	0.072210019	0.081026429	0.081026429	0.081026429	0.077340416	0.077340416	0.077340416	0.081984737	0.080984737	0.081084737	0.066410088	0.066410088	0.066410088	0.05688688	0.05688688	0.05688688	0.055633984	0.055633984	0.055633984	0.057205018	0.057205018	0.057205018	0.044775749	0.044775749	0.044775749
6-hydroxy-alpha-methylnaphthaleneacetic acid	0.198849625	0.198749625	0.198649625	0.196842271	0.196842271	0.196842271	0.185257623	0.195257623	0.205257623	0.295965121	0.295965121	0.295965121	0.210989462	0.210989462	0.210989462	0.367167644	0.357167644	0.347167644	0.282187101	0.282187101	0.282187101	0.314998504	0.314998504	0.314998504	0.319608272	0.319608272	0.319608272	0.290006032	0.290006032	0.290006032
L-alanine	1.595679599	1.137364541	1.085107258	0.81720469	0.81720469	0.81720469	1.085107258	1.585107258	1.285107258	1.633090539	1.533090539	1.433090539	0.969992283	0.969992283	0.969992283	0.883237856	0.893237856	0.889237856	0.965377234	0.955377234	0.925377234	0.688715657	0.688715657	0.688715657	0.777886396	0.777886396	0.777886396	0.4889801	0.4889801	0.4889801
D-erythro-sphingosine	0.000989424	0.000989424	0.000989424	0.000953438	0.000953438	0.000953438	0.001192162	0.001192162	0.001192162	0.001382925	0.001382925	0.001382925	0.001114826	0.001114826	0.001114826	0.000961997	0.000961997	0.000961997	0.000548444	0.000548444	0.000548444	0.00091034	0.00091034	0.00091034	0.000713903	0.000713903	0.000713903	0.000716497	0.000716497	0.000716497
Erucic acid	0.003358909	0.003358909	0.003358909	0.002485646	0.002485646	0.002485646	0.004915989	0.004915989	0.004915989	0.004088403	0.004088403	0.004088403	0.002074937	0.002074937	0.002074937	0.004635708	0.004935708	0.004835708	0.005709273	0.005709273	0.005709273	0.006239766	0.006239766	0.006239766	0.005586234	0.005586234	0.005586234	0.003776109	0.003776109	0.003776109
Abietic acid	0.000386389	0.000386389	0.000386389	0.000446569	0.000446569	0.000446569	0.000441921	0.000441921	0.000441921	0.000541663	0.000541663	0.000541663	0.000484271	0.000484271	0.000484271	0.000794597	0.000784597	0.000774597	0.000589648	0.000589648	0.000589648	0.000629474	0.000629474	0.000629474	0.000604225	0.000604225	0.000604225	0.000679242	0.000679242	0.000679242
Dimethylethanolamine	0.002255165	0.002255165	0.002255165	0.001896154	0.001896154	0.001896154	0.002999105	0.002999105	0.002999105	0.003567668	0.003567668	0.003567668	0.001783925	0.001783925	0.001783925	0.001525708	0.001525708	0.001525708	0.001071548	0.001071548	0.001071548	0.00184923	0.00184923	0.00184923	0.002063009	0.002063009	0.002063009	0.00144551	0.00144551	0.00144551
3-hydroxypropionic acid	0.00202345	0.00202345	0.00202345	0.000880041	0.000880041	0.000880041	0.001147979	0.001147979	0.001147979	0.00149057	0.00149057	0.00149057	0.001441443	0.001441443	0.001441443	0.000843308	0.000843308	0.000843308	0.001023963	0.001023963	0.001023963	0.001017796	0.001017796	0.001017796	0.000897874	0.000897874	0.000897874	0.000628817	0.000628817	0.000628817
Psilocin	0.000744049	0.000744049	0.000744049	0.000767346	0.000767346	0.000767346	0.00080507	0.00080507	0.00080507	0.001094469	0.001094469	0.001094469	0.000871148	0.000871148	0.000871148	0.001487599	0.001487599	0.001487599	0.001050068	0.001050068	0.001050068	0.001218529	0.001218529	0.001218529	0.001211246	0.001211246	0.001211246	0.001145778	0.001145778	0.001145778
2,3,4,5-tetrahydroxypentanoic acid-1,4-lactone	0.003963341	0.003963341	0.003963341	0.003721235	0.003721235	0.003721235	0.004487869	0.004487869	0.004487869	0.004414836	0.004414836	0.004414836	0.004085653	0.004185653	0.004005653	0.003091615	0.003091615	0.003091615	0.002794781	0.002794781	0.002794781	0.00322601	0.00322601	0.00322601	0.003010437	0.003110437	0.003210437	0.003107558	0.003107558	0.003107558
Spermine	0.006538031	0.006538031	0.006538031	0.002867591	0.002867591	0.002867591	0.004246348	0.004246348	0.004246348	0.00545062	0.00545062	0.00545062	0.004136282	0.004106282	0.004036282	0.002823313	0.002823313	0.002823313	0.00295518	0.00295518	0.00295518	0.003966424	0.003966424	0.003966424	0.002179601	0.002179601	0.002179601	0.003033471	0.003033471	0.003033471
4-methylvaleric acid	0.002183635	0.002183635	0.002183635	0.00215791	0.00215791	0.00215791	0.002107275	0.002107275	0.002107275	0.002047098	0.002047098	0.002047098	0.002077686	0.002077686	0.002077686	0.001574037	0.001574037	0.001574037	0.001491621	0.001491621	0.001491621	0.001755927	0.001755927	0.001755927	0.001474376	0.001474376	0.001474376	0.001570349	0.001570349	0.001570349

3.Supplement 3A：This is evidence of group1A and group4A-heatmap，cluster graph analysis representing metabolites. In order to more intuitively display the relationship between QC samples and other samples and the stability among QC samples, we conducted Hierarchical Clustering of all metabolite expression levels.

Supplement 3B

Metabolite name	23	43	46	29	45	48	24	44	47	14	49	52	19	51	54	18	50	53
2-oxo-propanoic acid	0.003896268	0.003896268	0.003896268	0.004239779	0.004239779	0.004239779	0.004836617	0.004836617	0.004836617	0.019824477	0.019824477	0.019824477	0.021704073	0.021704073	0.021704073	0.016472404	0.016472404	0.016472404
2-hydroxy-2-pentenedioic acid	0.001504013	0.001504013	0.001504013	0.00135036	0.00135036	0.00135036	0.001665197	0.001665197	0.001665197	0.008992969	0.008992969	0.008992969	0.003912471	0.003912471	0.003912471	0.005702403	0.005702403	0.005702403
1-deoxy-1-(4-morpholinyl)-alpha-d-fructopyranose	0.014543823	0.014543823	0.014543823	0.006849241	0.006849241	0.006849241	0.011033787	0.011033787	0.011033787	0.004257638	0.004257638	0.004257638	0.000900455	0.000900455	0.000900455	0.00352813	0.00352813	0.00352813
Aprobarbital	0.033620049	0.033620049	0.033620049	0.01557074	0.01557074	0.01557074	0.026473299	0.026473299	0.026473299	0.009564659	0.009564659	0.009564659	0.002794069	0.002794069	0.002794069	0.008046076	0.008046076	0.008046076
6(5h)-benzo[c]phenanthridinone, 11,12-dihydro-	0.041920547	0.041920547	0.041920547	0.023098573	0.023098573	0.023098573	0.035584043	0.035584043	0.035584043	0.013230939	0.013230939	0.013230939	0.006771941	0.006771941	0.006771941	0.0120766	0.0120766	0.0120766
(e)-9-tetradecenoic acid	0.001865214	0.001865214	0.001865214	0.002049759	0.002049759	0.002049759	0.003637944	0.003637944	0.003637944	0.000531544	0.000531544	0.000531544	0.001249219	0.001249219	0.001249219	0.000764213	0.000764213	0.000764213
Chenodeoxycholic acid	0.018111027	0.018111027	0.018111027	0.018100991	0.018100991	0.018100991	0.025177359	0.025177359	0.025177359	0.00887651	0.00887651	0.00887651	0.009676733	0.009676733	0.009676733	0.007863564	0.007863564	0.007863564
3-methyl-2-oxovaleric acid	0.00595051	0.00595051	0.00595051	0.00454116	0.00454116	0.00454116	0.007190517	0.007190517	0.007190517	0.002714326	0.002714326	0.002714326	0.002697867	0.002597867	0.002607867	0.002336632	0.002336632	0.002336632
Caffeic acid	0.002219585	0.002219585	0.002219585	0.002125423	0.002125423	0.002125423	0.001895731	0.001895731	0.001895731	0.000983619	0.000973619	0.000980619	0.001048488	0.001048488	0.001048488	0.000850266	0.000850266	0.000850266
Xanthosine	0.009210685	0.009210685	0.009210685	0.005357403	0.005357403	0.005357403	0.009899137	0.009899137	0.009899137	0.00414723	0.00414723	0.00414723	0.002832783	0.002832783	0.002832783	0.003422123	0.003422123	0.003422123
Heptadecanoic acid	0.003617414	0.003617414	0.003617414	0.001946635	0.001946635	0.001946635	0.003230972	0.003230972	0.003230972	0.001140153	0.001140153	0.001140153	0.001673643	0.001673643	0.001673643	0.000935963	0.000935963	0.000935963
D-arabinose	0.001008092	0.001008092	0.001008092	0.001742011	0.001742011	0.001742011	0.001243037	0.001243037	0.001243037	0.000460633	0.000460633	0.000460633	0.000790748	0.000790748	0.000790748	0.000552564	0.000552564	0.000552564
Metharbital	0.002238	0.002238	0.002238	0.002878356	0.002878356	0.002878356	0.001928531	0.001928531	0.001928531	0.001176269	0.001176269	0.001176269	0.000635534	0.000635534	0.000635534	0.001394276	0.001394276	0.001394276
Orotic acid	0.002740593	0.002740593	0.002740593	0.002120196	0.002120196	0.002120196	0.00325947	0.00325947	0.00325947	0.001493927	0.001493927	0.001493927	0.001199796	0.001199796	0.001199796	0.001157919	0.001147919	0.001150919
D-ribose	0.001008092	0.001008092	0.001008092	0.001777191	0.001777191	0.001777191	0.001301099	0.001501099	0.001601099	0.000502552	0.000502552	0.000502552	0.000818123	0.000818123	0.000818123	0.000552564	0.000552564	0.000552564
D-fucose	0.001824551	0.001824551	0.001824551	0.002204562	0.002204562	0.002204562	0.002240047	0.002240047	0.002240047	0.001068228	0.001068228	0.001068228	0.00099162	0.00099162	0.00099162	0.001171757	0.001171757	0.001171757
Behenic acid	0.005010287	0.005010287	0.005010287	0.005594676	0.005584676	0.005504676	0.005620657	0.004620657	0.005020657	0.003276871	0.003276871	0.003276871	0.002638017	0.002638017	0.002638017	0.00253281	0.00243281	0.00233281
Adenosine-5'-monophosphate	0.078149052	0.078149052	0.078149052	0.045994309	0.046994309	0.047994309	0.087219118	0.086219118	0.085219118	0.037717606	0.037717606	0.037717606	0.022917982	0.022917982	0.022917982	0.037565285	0.036565285	0.037065285
Benzoic acid	0.001286566	0.001286566	0.001286566	0.000796339	0.000786339	0.000799339	0.001158484	0.001158484	0.001158484	0.000569442	0.000569442	0.000569442	0.000524629	0.000534629	0.000544629	0.000618734	0.000618734	0.000618734
Pyrazin-2-carboxylic acid	0.015019043	0.015019043	0.015019043	0.008183954	0.008183954	0.008183954	0.013749169	0.012749169	0.013049169	0.006568349	0.006568349	0.006568349	0.006295915	0.006195915	0.006205915	0.006145794	0.006145794	0.006145794
2,4-diaminobutyric acid	0.000262995	0.000262995	0.000262995	0.000229606	0.000229606	0.000229606	0.000423116	0.000483116	0.000473116	0.000521227	0.000521227	0.000521227	0.000660898	0.000660898	0.000660898	0.000500965	0.000500965	0.000500965
Gluconic acid	0.014919776	0.014919776	0.014919776	0.011748007	0.011848007	0.011948007	0.018465746	0.019465746	0.018965746	0.007725636	0.007825636	0.007925636	0.009832806	0.009832806	0.009832806	0.007279	0.007379	0.007479
6-deoxyglucitol	0.00658961	0.00648961	0.00638961	0.010571068	0.010571068	0.010571068	0.010328795	0.010328795	0.010328795	0.005646688	0.005746688	0.005606688	0.0057257	0.0057257	0.0057257	0.003614966	0.003614966	0.003614966
D-xylose	0.005198784	0.005398784	0.005298784	0.00443833	0.00443833	0.00443833	0.003954923	0.003954923	0.003954923	0.002165878	0.002165878	0.002165878	0.00247751	0.00247751	0.00247751	0.003227153	0.003227153	0.003227153
(s)-3,4-dihydroxybutyric acid	0.001612999	0.001612999	0.001612999	0.001220232	0.001220232	0.001220232	0.001794749	0.001794749	0.001794749	0.000770123	0.000770123	0.000770123	0.001007353	0.001007353	0.001007353	0.000953945	0.000953945	0.000953945
D-xylulose	0.000723271	0.000723271	0.000723271	0.000748443	0.000708443	0.000728443	0.000800139	0.000790139	0.000780139	0.000268149	0.000268149	0.000268149	0.000551507	0.000561507	0.000550507	0.00051647	0.00051647	0.00051647
Kynurenic acid	0.013994113	0.013994113	0.013994113	0.012183059	0.012183059	0.012183059	0.015514262	0.015514262	0.015514262	0.008378271	0.008378271	0.008378271	0.009537886	0.009437886	0.009530886	0.008918844	0.008918844	0.008918844
Uracil	0.002864743	0.002864743	0.002864743	0.002355397	0.002455397	0.002755397	0.00293717	0.00293717	0.00293717	0.002042494	0.002042494	0.002042494	0.001556576	0.001556576	0.001556576	0.001803225	0.001803225	0.001803225
Boric acid	0.058920178	0.057920178	0.059920178	0.059498078	0.060498078	0.061498078	0.062681122	0.062681122	0.062681122	0.034344344	0.034344344	0.034344344	0.045330197	0.044330197	0.045030197	0.043289284	0.043289284	0.043289284
Citric acid	1.042376112	1.041376112	1.040376112	1.070885319	1.170885319	1.270885319	1.307467186	1.297467186	1.287467186	0.619435112	0.639435112	0.659435112	0.849480082	0.846480082	0.844480082	0.840563344	0.830563344	0.820563344
1-methylinosine	0.003766057	0.003766057	0.003766057	0.002611896	0.002611896	0.002611896	0.003442367	0.003442367	0.003442367	0.004378168	0.004378168	0.004378168	0.005150071	0.005150071	0.005150071	0.004874368	0.004774368	0.004804368
Glucose-1-phosphate	0.006297486	0.006297486	0.006297486	0.005550893	0.005550893	0.005550893	0.007640015	0.007640015	0.007640015	0.00443459	0.00443459	0.00443459	0.004877593	0.004877593	0.004877593	0.00391095	0.00391095	0.00391095
2-picolinic acid	0.00929165	0.00929165	0.00929165	0.007268909	0.007268909	0.007268909	0.00852521	0.00852521	0.00852521	0.010644295	0.010644295	0.010644295	0.012717933	0.012717933	0.012717933	0.012357826	0.012357826	0.012357826
Terephthalic acid	0.008860911	0.008860911	0.008860911	0.010594534	0.010594534	0.010594534	0.008622577	0.008622577	0.008622577	0.006438438	0.006438438	0.006438438	0.007510022	0.007510022	0.007510022	0.005874838	0.005874838	0.005874838
1,2,4-benzenetriol	0.019254465	0.019254465	0.019254465	0.015037843	0.015037843	0.015037843	0.018215171	0.018215171	0.018215171	0.011733944	0.011733944	0.011733944	0.013617328	0.013617328	0.013617328	0.012042718	0.012042718	0.012042718
Coniferin	0.000725263	0.000725263	0.000725263	0.000714306	0.000794306	0.000814306	0.000737865	0.000727865	0.000717865	0.000545808	0.000545808	0.000545808	0.000586684	0.000586684	0.000586684	0.00045433	0.00045433	0.00045433
Oleamide	0.001184711	0.001184711	0.001184711	0.000905022	0.000905022	0.000905022	0.001258774	0.001258774	0.001258774	0.0008291	0.0008291	0.0008291	0.000828021	0.000828021	0.000828021	0.00068776	0.00068776	0.00068776
5-hydroxy-3-indoleacetic acid	0.000920978	0.000920978	0.000920978	0.001055713	0.001055713	0.001055713	0.001233302	0.001233302	0.001233302	0.000735286	0.000735286	0.000735286	0.00076359	0.00076359	0.00076359	0.000812089	0.000812089	0.000812089
Piceatannol	0.000544537	0.000544537	0.000544537	0.000374152	0.000374152	0.000374152	0.000431357	0.000431357	0.000431357	0.000576745	0.000576745	0.000576745	0.000704601	0.000704601	0.000704601	0.000611149	0.000611149	0.000611149
Sinapinic acid	0.001145444	0.001145444	0.001145444	0.00087455	0.00087455	0.00087455	0.000922926	0.000912926	0.000902926	0.000699572	0.000699572	0.000699572	0.000765286	0.000755286	0.000745286	0.000641906	0.000641906	0.000641906
Dimethylethanolamine	0.001958891	0.001958891	0.001958891	0.001564896	0.001564896	0.001564896	0.001986769	0.001986769	0.001986769	0.001338327	0.001338327	0.001338327	0.00131229	0.00135229	0.00136229	0.001357754	0.001357754	0.001357754
D-fructose-1-phosphate	0.00031103	0.00031103	0.00031103	0.000274932	0.000274932	0.000274932	0.000298822	0.000298822	0.000298822	0.000228652	0.000228652	0.000228652	0.000219706	0.000219706	0.000219706	0.00022688	0.00022688	0.00022688

3.Supplement 3B：This is evidence of group2A and group3A-heatmap，cluster graph analysis representing metabolites. In order to more intuitively display the relationship between QC samples and other samples and the stability among QC samples, we conducted Hierarchical Clustering of all metabolite expression levels.

Supplement 3C

Metabolite name	2	16	38	5	35	40	43	46
Lactitol	0.301927774	0.301927774	0.301927774	0.302578679	0.302578679	0.302578679	0.002541129	0.002541129
Sorbitol	0.037765493	0.037765493	0.037765493	0.084483189	0.084483189	0.084483189	0.085268486	0.084268486
2-hydroxy -2-pentenedioic acid	0.000133123	0.000133123	0.000133123	6.86902E -05	6.86902E -05	6.86902E -05	0.001504013	0.001504013
Palmitic acid	0.001554413	0.001554413	0.001554413	0.001167106	0.001167106	0.001167106	0.003192656	0.003192656
Caffeic acid	0.003349568	0.003349568	0.003349568	0.002228413	0.002228413	0.002228413	0.002219585	0.002219585
3-hydroxyestra -1,3,5(10) -triene -6,17 -dione	0.000758303	0.000758303	0.000758303	0.000735756	0.000735756	0.000735756	0.001350739	0.001350739
Gallic acid	0.128266799	0.128266799	0.128266799	0.094309725	0.094309725	0.094309725	0.094348712	0.094348712
Pyrazin -2-carboxylic acid	0.01233121	0.01233121	0.01233121	0.015500327	0.015400327	0.015500327	0.015019043	0.015019043
L-tryptophan	0.682704973	0.682704973	0.682704973	0.490064212	0.490064212	0.490064212	0.433139958	0.433139958
Epicatechin	0.003194223	0.003194223	0.003194223	0.00232243	0.002632639	0.002228228	0.002632639	0.002632639
D-ribose	0.001320716	0.001320716	0.001320716	0.000901324	0.001008092	0.000901324	0.001008092	0.001008092
Chenodeoxycholic acid	0.016091761	0.016091761	0.016091761	0.007867789	0.007867789	0.007867789	0.018111027	0.018111027
Gluconic acid	0.015969714	0.015969714	0.015969714	0.015981129	0.015981129	0.015981129	0.013919776	0.012919776
Galacturonic acid	0.014005552	0.014005552	0.014005552	0.013375633	0.013375633	0.013375633	0.020371108	0.020371108
Sedoheptulose	0.011789934	0.011789934	0.011789934	0.010446995	0.010446995	0.010446995	0.010883127	0.010883127
Glucose -1-phosphate	0.007578817	0.007578817	0.007578817	0.007222587	0.007640015	0.006297486	0.006297486	0.006297486
D-myo -inositol 4-phosphate	0.022966214	0.022966214	0.022966214	0.025216951	0.025216951	0.025216951	0.038937774	0.038937774
D-arabinose	0.001255741	0.001255741	0.001255741	0.00085225	0.00085225	0.00085225	0.001008092	0.001008092
Saccharic acid	0.00305249	0.00305249	0.00305249	0.002893439	0.002893439	0.002893439	0.004936594	0.004936594
Cholesterone	0.008652543	0.008652543	0.008113896	0.008113896	0.008013896	0.007913896	0.011247346	0.011247346
Coniferin	0.000742089	0.000725263	0.000742089	0.001051198	0.001051198	0.001051198	0.000725263	0.000725263
Quinic acid	0.003886949	0.003886949	0.003886949	0.004024056	0.004024056	0.004024056	0.004394949	0.004394949
1-deoxy -1-piperidino -alpha -d-fructose	0.000899512	0.000847309	0.000847309	0.001140239	0.001140239	0.001140239	0.000881839	0.000881839
Butane -2,3-diol	0.002407819	0.001029387	0.000629858	0.001633627	0.001786379	0.001676343	0.001676343	0.001676343
N-carbamoylaspartate	0.00568935	0.00568935	0.00568935	0.002789171	0.029825452	0.028825452	0.022339756	0.022339756
Alpha -tocopherol	0.225166189	0.225166189	0.225166189	0.320264095	0.320264095	0.320264095	0.234358662	0.234358662
Kynurenic acid	0.017124066	0.017124066	0.017124066	0.016526579	0.016526579	0.016526579	0.013994113	0.013994113
Xylonolactone	0.007571481	0.007571481	0.007571481	0.007220207	0.007220207	0.007220207	0.006293372	0.006293372
D-fucose	0.001352256	0.001352256	0.001352256	0.001893104	0.001893104	0.001893104	0.001824551	0.001824551
(s)-3,4-dihydroxybutyric acid	0.001273034	0.001273034	0.001273034	0.001348791	0.001348791	0.001348791	0.001612999	0.001612999
3-hydroxybenzoic acid	0.001803081	0.001803081	0.001803081	0.001102922	0.001102922	0.001102922	0.001028136	0.001028136
Methanephosphonothioic acid	0.016340839	0.016340839	0.016340839	0.022940477	0.020147492	0.019640309	0.025597179	0.025597179
Ethyl beta -d-galactofuranoside	0.018809973	0.018809973	0.018809973	0.018629818	0.018629818	0.018629818	0.013111231	0.013111231
Cis -gondoic acid	0.005097195	0.005097195	0.005097195	0.006073052	0.006073052	0.006073052	0.006092384	0.006092384
Taurine	0.034671427	0.034671427	0.034671427	0.019118146	0.019118146	0.019118146	0.018249117	0.018249117
Benzoic acid	0.001462889	0.001462889	0.001286566	0.001517791	0.001517791	0.001517791	0.001286566	0.001286566
Adenine	0.002786405	0.001412156	0.001412156	0.002262205	0.002262205	0.002262205	0.002194925	0.002194925
Melezitose	0.379247588	0.302568511	0.244114023	0.401046661	0.401046661	0.401046661	0.244114023	0.244114023
Digalacturonic acid	0.001276368	0.001276368	0.001276368	0.001017741	0.001017741	0.001017741	0.000660488	0.000660488
2-ketoisocaproic acid	0.010128907	0.010128907	0.010128907	0.014693047	0.014693047	0.014693047	0.01705906	0.01705906
Galactitol	0.0037426	0.0037426	0.0037426	0.003289182	0.003189182	0.003179182	0.002587453	0.002587453
Boric acid	0.083933049	0.058920178	0.067123331	0.067123331	0.059498078	0.058920178	0.058920178	0.058920178
Cholesterol	8.670486795	9.847899993	7.828517734	8.081183705	8.081183705	8.081183705	8.847899993	8.847899993
2,4-diaminobutyric acid	0.000125034	0.000339134	0.000500132	0.000111988	0.000111988	0.000111988	0.000262995	0.000262995
1,2,4 -benzenetriol	0.01923027	0.018215171	0.018323628	0.017950915	0.017950915	0.017950915	0.019254465	0.019254465
Alizarin	0.002599077	0.002576623	0.002576623	0.002017984	0.002017984	0.002017984	0.00167935	0.00167935
Dihydrocholesterol	0.007938094	0.0079798	0.007777884	0.0079798	0.0079798	0.0079798	0.010615271	0.010615271
Oleamide	0.001073583	0.001048374	0.001258774	0.00118723	0.00118723	0.00118723	0.001184711	0.001184711
Galactaric acid	0.002809342	0.002809342	0.002809342	0.002096653	0.002086653	0.002112564	0.003000626	0.003000626
2-ketoisovaleric acid	0.004146636	0.003681271	0.003681271	0.007630099	0.007630099	0.007630099	0.003681271	0.003681271
6-deoxyglucitol	0.007127718	0.007127718	0.007127718	0.010105146	0.010105146	0.010105146	0.00658961	0.00658961
L-methionine	0.072903131	0.07982697	0.062903131	0.055413839	0.055413839	0.055413839	0.039136266	0.049136266
2-piperidinobenzonitrile	0.010304745	0.026172939	0.026172939	0.012222939	0.012222939	0.012222939	0.024740626	0.024740626
L-isoleucine	0.272235861	0.272235861	0.272235861	0.287582997	0.277582997	0.267582997	0.257665204	0.247665204
N-acetylglutamate	0.001449341	0.001449341	0.001449341	0.000931284	0.000931284	0.000931284	0.001960333	0.001960333
Lactobionic acid	0.014902096	0.014902096	0.014902096	0.014203447	0.014203447	0.014203447	0.019207217	0.019207217
Glycerol -3-galactoside	0.001570551	0.001570551	0.001570551	0.001111587	0.001111587	0.001111587	0.006338694	0.006338694
L-arabitol	0.011104407	0.011104407	0.011104407	0.012582463	0.012582463	0.012582463	0.011004364	0.011004364
Phosphenodiimidic amide	0.001453415	0.001453415	0.001453415	0.001463058	0.001463058	0.001463058	0.002105052	0.002105052
Sulfuric acid	0.001667601	0.001667601	0.001667601	0.001420018	0.001384981	0.001264773	0.001518947	0.001518947
Triacetin	0.181443886	0.181443886	0.181443886	0.103544556	0.103544556	0.103544556	0.086008831	0.085008831
Metharbital	0.001777073	0.001777073	0.001777073	0.003083091	0.003083091	0.003083091	0.002238	0.002238
2-oxo-propanoic acid	0.00410032	0.00410032	0.00410032	0.002959976	0.002959976	0.002959976	0.003896268	0.003896268
Stigmasterol	0.072765702	0.072765702	0.072765702	0.097784456	0.087784456	0.080229375	0.115115669	0.115115669
Talose	0.005866428	0.005866428	0.005866428	0.004229089	0.004219089	0.004129089	0.003037455	0.003037455
D-xylulose	0.000786498	0.000786498	0.000786498	0.000695233	0.000695233	0.000695233	0.000723271	0.000723271
5-hydroxy -3-indoleacetic acid	0.000667059	0.000667059	0.000667059	0.000837876	0.000837876	0.000837876	0.000920978	0.000920978
Aconitic acid	0.003087212	0.002968789	0.002968789	0.003318594	0.003318594	0.003318594	0.003759476	0.003759476
Spermine	0.006538031	0.00645062	0.005856898	0.002867591	0.002867591	0.002867591	0.005856898	0.005856898
Uracil	0.003022433	0.00293737	0.00317209	0.002266498	0.002266498	0.002266498	0.002864743	0.002864743
Isochlorogenic acid	0.000942084	0.000826394	0.000942084	0.000826394	0.000826394	0.000826394	0.002980469	0.002980469
Dehydroascorbic acid	0.003264712	0.002954982	0.003064712	0.002762705	0.002754351	0.002759348	0.002759348	0.002759348
Creatinine	0.022191622	0.023281284	0.022444409	0.023281284	0.023281284	0.023281284	0.053410805	0.053410805
Leucine	0.589509097	0.605148841	0.561017472	0.605148841	0.605148841	0.605148841	0.561017472	0.561017472
Carbamic acid	0.091593784	0.08748955	0.079797199	0.094689241	0.094689241	0.094689241	0.176464209	0.176464209
Hydroxypropanedioic acid	0.000315316	0.000361885	0.00034589	0.000361885	0.000361885	0.000361885	0.000407895	0.000407895
2-monostearin	0.004386468	0.004386468	0.004386468	0.003634156	0.003634156	0.003634156	0.00402198	0.00402198
3-methyl -2-oxovaleric acid	0.002246415	0.002246415	0.002246415	0.002923835	0.002923835	0.002923835	0.00595051	0.00595051
Carbamazepine	0.204445914	0.204445914	0.204445914	0.123198086	0.122198086	0.123198086	0.135170024	0.135170024
Sinapinic acid	0.001067416	0.001067416	0.001067416	0.001024007	0.001024007	0.001024007	0.001145444	0.001145444
Indolelactic acid	0.035427207	0.035427207	0.035427207	0.028053632	0.028053632	0.028053632	0.060029714	0.060029714
Terephthalic acid	0.00801414	0.00801414	0.00801414	0.006641584	0.006641584	0.006641584	0.006860911	0.006860911
Aminomalonate	0.37030759	0.37030759	0.37030759	0.175321876	0.175321876	0.175321876	0.415540045	0.405540045
Cyclohexylamine	0.000914509	0.000914509	0.000914509	0.000994262	0.000994262	0.000994262	0.001467915	0.001467915
L-tyrosine	1.495708796	1.495708796	1.495708796	0.888071126	0.888071126	0.888071126	0.959195381	0.949195381
Epsilon -caprolactam	0.001314008	0.001314008	0.001314008	0.0010489	0.0010489	0.0010489	0.0023859	0.0023859
1-methylinosine	0.002184342	0.002184342	0.002184342	0.002152465	0.002152465	0.002152465	0.003766057	0.003766057
Dimethylethanolamine	0.002255165	0.002255165	0.002255165	0.001896154	0.001896154	0.001896154	0.001958891	0.001958891
Methylamine	1.724209652	1.724209652	1.724209652	1.761681697	1.761681697	1.761681697	2.267724187	2.267724187
Malonic acid	0.000419397	0.000419397	0.000419397	0.000388342	0.000388342	0.000388342	0.000345161	0.000345161
Trisaccharide	0.494406371	0.494406371	0.494406371	0.346043697	0.346043697	0.346043697	0.754368616	0.756368616
Citric acid	0.825469656	0.825469656	0.825469656	1.044054025	1.044054025	1.044054025	1.042376112	1.042376112
Glycerol	0.159571861	0.159571861	0.159571861	0.228639965	0.228639965	0.228639965	0.138979009	0.138979009
Guanosine	0.001468634	0.001468634	0.001468634	0.000834961	0.000834961	0.000834961	0.000602099	0.000602099
Adipic acid	0.000408944	0.000408944	0.000408944	1.77949E -05	1.77949E -05	1.77949E -05	0.000445384	0.000445384
4-methylvaleric acid	0.002183635	0.002183635	0.002183635	0.00215791	0.00215791	0.00215791	0.001963585	0.001963585
Maleimide	0.005162139	0.005162139	0.005162139	0.003697715	0.003597715	0.003605684	0.004279372	0.004279372
Erythritol	0.012922248	0.012922248	0.012922248	0.015295111	0.015295111	0.015295111	0.014308772	0.014308772
3-hydroxypropionic acid	0.00202345	0.00202345	0.00202345	0.000880041	0.000763642	0.000712009	0.001621839	0.001621839
L-phenylalanine	0.110937189	0.108845684	0.104143197	0.115045864	0.115045864	0.115045864	0.088480525	0.088480525
L-alanine	1.595679599	1.137364541	1.085107258	0.81720469	0.81720469	0.81720469	1.137364541	1.138364541
2,6-bis(tert -butyl)phenol	0.013734769	0.013255306	0.013076675	0.013255306	0.013255306	0.013255306	0.013076675	0.013076675
Barbital	0.006950239	0.006950239	0.006950239	0.00501282	0.00501282	0.00501282	0.005680141	0.005680141
2-picolinic acid	0.006165349	0.006165349	0.006165349	0.005841515	0.005841515	0.005841515	0.00929165	0.00929165
2-ketobutyric acid	0.002983235	0.002983235	0.002983235	0.002288259	0.002288259	0.002288259	0.002819219	0.002819219
Piceatannol	0.000316428	0.000316428	0.000316428	0.00027761	0.00027761	0.00027761	0.000544537	0.000544537
Udp-n-acetylglucosamine	0.001321934	0.001321934	0.001321934	0.000774448	0.000774448	0.000774448	0.000951353	0.000951353
L-kynurenine	0.003827781	0.003827781	0.003827781	0.003874876	0.003999774	0.003899774	0.004001381	0.004001381
1-monostearin	0.076885408	0.076885408	0.076885408	0.068799224	0.068799224	0.068799224	0.119316769	0.118516769
Resorcinol	0.003301217	0.003268502	0.003097917	0.002878321	0.002878321	0.002878321	0.003097917	0.003097917
2,3,4,5 -tetrahydroxypentanoic acid-1,4 -lactone	0.003963341	0.00389811	0.003721235	0.003721235	0.003721235	0.003721235	0.004863603	0.004863603
Phytosphingosine	0.001321934	0.001321934	0.001321934	0.000778226	0.000778226	0.000778226	0.000951353	0.000951353

3.Supplement 3C：This is evidence of group1A -2A and group3A -4A-heatmap， cluster graph analysis representing metabolites. In order to more intuitively display the relationship between QC samples and other samples and the stability among QC samples, we conducted Hierarchical Clustering of all metabolite expression levels.
