# Supplementary material for: Study on the Metabonomics Mechanism of Mongolian Medical Andai Therapy on Healthy People
Source: Evid Based Complement Alternat Med. 2022 Jun 20;2022:1364408. doi: 10.1155/2022/1364408 (PMC9236767; doi:10.1155/2022/1364408)
Supplement: Supplementary Materials — Supplement 1 is evidence for the principal component analysis (PCA) diagram. Supplement 2A is evidence for (group 1-group 4) female sample comparison volcanic map analysis representing metabolites, as shown in Figure 6. Supplement 2B is evidence for (group 2-group 3) male sample comparison volcanic map analysis representing metabolites, as shown in Figure 7. Supplement2C is evidence for (group 1-group 2 and group 3-group 4) full-sample comparison volcanic map analysis representing metabolites, as shown in Figure 5. Supplement 3A is evidence for (group 1A and group 4A) female sample clustering heat map analysis, as shown in Figure 9. Supplement 3B is evidence for (group 2A and group 3A) male sample clustering heat map analysis, as shown in Figure 10. Supplement 3C is evidence for (group 1A-2A and group 3A-4A) whole-sample clustering heat map analysis， as shown in Figure 8. Supplement 4A is evidence for (group 1A and group 4A) accumulation of metabolic pathways in female samples—Top20, as shown in Figure 13. Supplement 4B is evidence for (group 2 and group 3) accumulation of metabolic pathways in male samples—Top20, as shown in Figure 15. Supplement 4C is evidence for (group 1-2 and group 3-4) enrichment of metabolic pathways in the whole sample—Top20, as shown in Figure 11. Supplement 5A is evidence for (group 1 and group 4) metabolic bubble of female sample, as shown in Figure 14. Supplement 5B is evidence for (group 2 and group 3) metabolic bubble of male sample, as shown in Figure 16. Supplement 5C is evidence for metabolic pathways of (group 1-2 and group 3-4) metabolic bubble of the whole sample, as shown in Figure 12. [file 1364408.f1.zip › 1364408.f1/Supplement 4C.pdf]

Supplement 4C

Metabolite name	log2(FC)	KEGG
1,2,4-benzenetriol	0.610861651	C02814
2-ketobutyric acid	0.593227077	C00109
2-ketoisocaproic acid	0.767677677	C00233
2-ketoisovaleric acid	0.789203184	C00141
2-picolinic acid	-0.567114568	C10164
3-hydroxybenzoic acid	0.858359097	C00587
3-hydroxypropionic acid	0.548778284	C01013
3-methyl-2-oxovaleric acid	0.69755374	C03465
5-hydroxy-3-indoleacetic acid	0.675141739	C05635
Aconitic acid	0.633558663	C00417
Adenine	0.915764566	C00147
Adipic acid	0.94630655	C06104
Alpha-tocopherol	0.924327839	C02477
Aminomalonate	0.672456965	C00872
Benzoic acid	0.789501536	C00539
Butane-2,3-diol	0.929153895	C00265
Caffeic acid	1.747512164	C01481
Carbamazepine	0.686714031	C06868
Carbamic acid	0.735112366	C01563
Chenodeoxycholic acid	1.207215989	C02528
Cholesterol	0.658125092	C00187
Cholesterone	0.98089512	C00599
Cis-gondoic acid	0.861825345	C16526
Citric acid	0.505090824	C00158
Coniferin	0.999182792	C00761
Creatinine	0.675156584	C00791
Cyclohexylamine	-0.492635382	C00571
D-fucose	0.906827027	C01018
D-myo-inositol 4-phosphate	1.009152075	C03546
D-ribose	1.199817264	C00121
D-xylulose	0.667739838	C00310
Dehydroascorbic acid	0.624074203	C05422
Digalacturonic acid	0.963119813	C02273
Dimethylethanolamine	0.59037946	C04308
Epicatechin	1.088715214	C09727
Epsilon-caprolactam	0.571584498	C06593
Erythritol	0.600626817	C00503
Galactaric acid	0.711889263	C00879
Galactitol	0.791671688	C01697
Galacturonic acid	1.153617036	C08348
Gallic acid	1.330733695	C01424
Gluconic acid	1.067397337	C00257
Glucose-1-phosphate	0.971192891	C00103
Glycerol	0.574961524	C00116
Guanosine	0.785233778	C00387
Hydroxypropanedioic acid	0.658737674	C02287
Indolelactic acid	0.724236018	C02043

Kynurenic acid	0.784960902	C01717
L-alanine	0.575686259	C00041
L-arabitol	0.719428082	C00532
L-isoleucine	0.749088669	C00407
L-kynurenine	0.389519495	C00328
L-methionine	0.772396071	C00073
L-phenylalanine	0.570531844	C00079
L-tryptophan	1.185379419	C00078
L-tyrosine	0.676425671	C00082
Leucine	0.66624482	C00123
Malonic acid	0.459411269	C04025
Melezitose	0.883914374	C08243
Methylamine	0.475740163	C00218
N-acetylglutamate	0.78491888	C00624
N-carbamoylaspartate	1.403496967	C00438
Oleamide	0.609732747	C19670
Palmitic acid	2.064349767	C00249
Phytosphingosine	0.489141085	C12144
Piceatannol	-0.58597481	C05901
Pyrazin-2-carboxylic acid	1.234445741	C19915
Quinic acid	1.316259659	C00296
Saccharic acid	1.023638579	C00818
Sedoheptulose	1.142773526	C08355
Sinapinic acid	0.46664404	C00482
Sorbitol	2.267170111	C00794
Spermine	0.779387726	C00750
Stigmasterol	0.666893661	C05442
Sulfuric acid	0.599995904	C00059
Taurine	0.997486847	C00245
Terephthalic acid	0.536900351	C06337
Udp-n-acetylglucosamine	0.508638222	C00043
Uracil	0.598099271	C00106
Xylonolactone	0.87272078	C02266

Supplement 4C: Enrichment map analysis evidence of metabolic pathways in Group1-2 and Group3-4. pathway analysis was performed on all samples of control group and experimental group. KEGG(https://www.kegg.jp/) database was used to analyze metabolic pathway enrichment of differen metabolites. Enrichment factor (Rich factor= number of significantly differentiated metabolites/total r in this pathway), the higher the Rich factor, the greater the degree of enrichment; The color from red indicates that p-values decrease successively. The redness indicates that the number of metabolites e this pathway is higher.

RED/BLUE
red
red
red
red
blue
red
red
red
red
red
red
red
red
red
red
red
red
red
red
red
red
red
red
red
red
red
blue
red
red
red
red
red
red
red
red
red
red
red
red
red
red
red
red
red
red
red
red

red
red
red
red
red
red
red
red
red
red
red
red
red
red
red
red
red
red
blue
red
red
red
red
red
red
red
red
red
red
red
red
red
red

Metabolic

ıtial
ıetabolites
to green
ıriched in
