# Supplementary material for: Study on the Metabonomics Mechanism of Mongolian Medical Andai Therapy on Healthy People
Source: Evid Based Complement Alternat Med. 2022 Jun 20;2022:1364408. doi: 10.1155/2022/1364408 (PMC9236767; doi:10.1155/2022/1364408)
Supplement: Supplementary Materials — Supplement 1 is evidence for the principal component analysis (PCA) diagram. Supplement 2A is evidence for (group 1-group 4) female sample comparison volcanic map analysis representing metabolites, as shown in Figure 6. Supplement 2B is evidence for (group 2-group 3) male sample comparison volcanic map analysis representing metabolites, as shown in Figure 7. Supplement2C is evidence for (group 1-group 2 and group 3-group 4) full-sample comparison volcanic map analysis representing metabolites, as shown in Figure 5. Supplement 3A is evidence for (group 1A and group 4A) female sample clustering heat map analysis, as shown in Figure 9. Supplement 3B is evidence for (group 2A and group 3A) male sample clustering heat map analysis, as shown in Figure 10. Supplement 3C is evidence for (group 1A-2A and group 3A-4A) whole-sample clustering heat map analysis， as shown in Figure 8. Supplement 4A is evidence for (group 1A and group 4A) accumulation of metabolic pathways in female samples—Top20, as shown in Figure 13. Supplement 4B is evidence for (group 2 and group 3) accumulation of metabolic pathways in male samples—Top20, as shown in Figure 15. Supplement 4C is evidence for (group 1-2 and group 3-4) enrichment of metabolic pathways in the whole sample—Top20, as shown in Figure 11. Supplement 5A is evidence for (group 1 and group 4) metabolic bubble of female sample, as shown in Figure 14. Supplement 5B is evidence for (group 2 and group 3) metabolic bubble of male sample, as shown in Figure 16. Supplement 5C is evidence for metabolic pathways of (group 1-2 and group 3-4) metabolic bubble of the whole sample, as shown in Figure 12. [file 1364408.f1.zip › 1364408.f1/Supplement 5A.pdf]

Supplement 5A

ID Annotation	Annotation	in set	set	in background	background	RichFactor	p-value	-lg(p-value)	FDR correction	Matching IDs	URL
hsa00970	Aminoacyl-tRNA biosynthesis	14	57	52	3250	0.269230769	5.16746E-14	13.28672314	5.16746E-12	C00037 C00041 C00064 C00065 C00073 C00078 C00079 C00082 C00097 C00123 C00135 C00148 C00188 C00407	https://www.genome.jp/kegg-bin/show_pathway?hsa00970/hsa:C00037%09red/hsa:C00041%09red/hsa:C00064%09red/hsa:C00065%09red/hsa:C00073%09red/hsa:C00078%09red/hsa:C00079%09red/hsa:C00082%09red/hsa:C00097%09red/hsa:C00123%09red/hsa:C00135%09red/hsa:C00148%09red/hsa:C00188%09red/hsa:C00407%09red
hsa05230	Central carbon metabolism in cancer	7	57	30	3250	0.233333333	5.2153E-07	6.282721024	2.60765E-05	C00037 C00041 C00064 C00065 C00097 C00123 C00148	https://www.genome.jp/kegg-bin/show_pathway?hsa05230/hsa:C00037%09red/hsa:C00041%09red/hsa:C00064%09red/hsa:C00065%09red/hsa:C00097%09red/hsa:C00123%09red/hsa:C00148%09red
hsa04974	Protein digestion and absorption	6	57	29	3250	0.206896552	7.74518E-06	5.110968664	0.000258173	C00037 C00041 C00064 C00079 C00097 C00148	https://www.genome.jp/kegg-bin/show_pathway?hsa04974/hsa:C00037%09red/hsa:C00041%09red/hsa:C00064%09red/hsa:C00079%09red/hsa:C00097%09red/hsa:C00148%09red
hsa02010	ABC transporters	9	57	93	3250	0.096774194	2.52658E-05	4.597466321	0.000631646	C00041 C00059 C00064 C00121 C00135 C00245 C00503 C00794 C02273	https://www.genome.jp/kegg-bin/show_pathway?hsa02010/hsa:C00041%09red/hsa:C00059%09red/hsa:C00064%09red/hsa:C00121%09red/hsa:C00135%09red/hsa:C00245%09red/hsa:C00503%09red/hsa:C00794%09red/hsa:C02273%09red
hsa00290	Valine, leucine and isoleucine biosynthesis	5	57	23	3250	0.217391304	3.67291E-05	4.434989285	0.000734583	C00123 C00141 C00188 C00233 C00407	https://www.genome.jp/kegg-bin/show_pathway?hsa00290/hsa:C00123%09red/hsa:C00141%09red/hsa:C00188%09red/hsa:C00233%09red/hsa:C00407%09red
hsa00410	beta-Alanine metabolism	5	57	32	3250	0.15625	0.000194832	3.710339292	0.003247203	C00106 C00135 C00334 C00750 C01013	https://www.genome.jp/kegg-bin/show_pathway?hsa00410/hsa:C00106%09red/hsa:C00135%09red/hsa:C00334%09red/hsa:C00750%09red/hsa:C01013%09red
hsa00400	Phenylalanine, tyrosine and tryptophan biosynthesis	5	57	34	3250	0.147058824	0.0002621	3.581533429	0.003744282	C00078 C00079 C00082 C00296 C00587	https://www.genome.jp/kegg-bin/show_pathway?hsa00400/hsa:C00078%09red/hsa:C00079%09red/hsa:C00082%09red/hsa:C00296%09red/hsa:C00587%09red
hsa00260	Glycine, serine and threonine metabolism	5	57	50	3250	0.1	0.001611398	2.792797288	0.018441408	C00037 C00065 C00078 C00097 C00188	https://www.genome.jp/kegg-bin/show_pathway?hsa00260/hsa:C00037%09red/hsa:C00065%09red/hsa:C00078%09red/hsa:C00097%09red/hsa:C00188%09red
hsa00770	Pantothenate and CoA biosynthesis	4	57	30	3250	0.133333333	0.001659727	2.779963421	0.018441408	C00097 C00106 C00141 C00750	https://www.genome.jp/kegg-bin/show_pathway?hsa00770/hsa:C00097%09red/hsa:C00106%09red/hsa:C00141%09red/hsa:C00750%09red
hsa00920	Sulfur metabolism	4	57	33	3250	0.121212121	0.002383212	2.622837312	0.023832121	C00059 C00065 C00097 C00245	https://www.genome.jp/kegg-bin/show_pathway?hsa00920/hsa:C00059%09red/hsa:C00065%09red/hsa:C00097%09red/hsa:C00245%09red
hsa00480	Glutathione metabolism	4	57	38	3250	0.105263158	0.004028223	2.394886444	0.036620213	C00037 C00097 C00750 C05422	https://www.genome.jp/kegg-bin/show_pathway?hsa00480/hsa:C00037%09red/hsa:C00097%09red/hsa:C00750%09red/hsa:C05422%09red
hsa00270	Cysteine and methionine metabolism	5	57	63	3250	0.079365079	0.004495043	2.347266158	0.037458691	C00041 C00059 C00065 C00073 C00097	https://www.genome.jp/kegg-bin/show_pathway?hsa00270/hsa:C00041%09red/hsa:C00059%09red/hsa:C00065%09red/hsa:C00073%09red/hsa:C00097%09red
hsa00280	Valine, leucine and isoleucine degradation	4	57	41	3250	0.097560976	0.005315208	2.274479711	0.040886218	C00123 C00141 C00233 C00407	https://www.genome.jp/kegg-bin/show_pathway?hsa00280/hsa:C00123%09red/hsa:C00141%09red/hsa:C00233%09red/hsa:C00407%09red
hsa00430	Taurine and hypotaurine metabolism	3	57	22	3250	0.136363636	0.006218769	2.206295559	0.04441978	C00041 C00097 C00245	https://www.genome.jp/kegg-bin/show_pathway?hsa00430/hsa:C00041%09red/hsa:C00097%09red/hsa:C00245%09red
hsa00120	Primary bile acid biosynthesis	4	57	47	3250	0.085106383	0.008660623	2.062450848	0.055930039	C00037 C00187 C00245 C02528	https://www.genome.jp/kegg-bin/show_pathway?hsa00120/hsa:C00037%09red/hsa:C00187%09red/hsa:C00245%09red/hsa:C02528%09red
hsa00600	Sphingolipid metabolism	3	57	25	3250	0.12	0.008948806	2.048234894	0.055930039	C00065 C00319 C12144	https://www.genome.jp/kegg-bin/show_pathway?hsa00600/hsa:C00065%09red/hsa:C00319%09red/hsa:C12144%09red
hsa04727	GABAergic synapse	2	57	9	3250	0.222222222	0.010054104	1.997656606	0.059141791	C00064 C00334	https://www.genome.jp/kegg-bin/show_pathway?hsa04727/hsa:C00064%09red/hsa:C00334%09red
hsa00250	Alanine, aspartate and glutamate metabolism	3	57	28	3250	0.107142857	0.012281958	1.910732394	0.0682331	C00041 C00064 C00334	https://www.genome.jp/kegg-bin/show_pathway?hsa00250/hsa:C00041%09red/hsa:C00064%09red/hsa:C00334%09red
hsa04122	Sulfur relay system	2	57	11	3250	0.181818182	0.015018706	1.823367477	0.077331058	C00041 C00097	https://www.genome.jp/kegg-bin/show_pathway?hsa04122/hsa:C00041%09red/hsa:C00097%09red
hsa00040	Pentose and glucuronate interconversions	4	57	56	3250	0.071428571	0.015874559	1.799298344	0.077331058	C00103 C00532 C02266 C02273	https://www.genome.jp/kegg-bin/show_pathway?hsa00040/hsa:C00103%09red/hsa:C00532%09red/hsa:C02266%09red/hsa:C02273%09red
hsa00730	Thiamine metabolism	3	57	31	3250	0.096774194	0.016239522	1.789426754	0.077331058	C00037 C00082 C00097	https://www.genome.jp/kegg-bin/show_pathway?hsa00730/hsa:C00037%09red/hsa:C00082%09red/hsa:C00097%09red
hsa05217	Basal cell carcinoma	1	57	1	3250	1	0.017538462	1.756008505	0.07972028	C00187	https://www.genome.jp/kegg-bin/show_pathway?hsa05217/hsa:C00187%09red
hsa05131	Shigellosis	2	57	14	3250	0.142857143	0.024027059	1.619299387	0.100993684	C00123 C00407	https://www.genome.jp/kegg-bin/show_pathway?hsa05131/hsa:C00123%09red/hsa:C00407%09red
hsa00230	Purine metabolism	5	57	95	3250	0.052631579	0.024238484	1.615494542	0.100993684	C00037 C00059 C00064 C00147 C00387	https://www.genome.jp/kegg-bin/show_pathway?hsa00230/hsa:C00037%09red/hsa:C00059%09red/hsa:C00064%09red/hsa:C00147%09red/hsa:C00387%09red
hsa04071	Sphingolipid signaling pathway	2	57	15	3250	0.133333333	0.027415153	1.562009334	0.10966061	C00065 C00319	https://www.genome.jp/kegg-bin/show_pathway?hsa04071/hsa:C00065%09red/hsa:C00319%09red
hsa04080	Neuroactive ligand-receptor interaction	3	57	40	3250	0.075	0.031956956	1.495434602	0.122911367	C00037 C00245 C00334	https://www.genome.jp/kegg-bin/show_pathway?hsa04080/hsa:C00037%09red/hsa:C00245%09red/hsa:C00334%09red
hsa04726	Serotonergic synapse	2	57	17	3250	0.117647059	0.034725078	1.459356768	0.128611401	C00078 C05635	https://www.genome.jp/kegg-bin/show_pathway?hsa04726/hsa:C00078%09red/hsa:C05635%09red
hsa00052	Galactose metabolism	3	57	46	3250	0.065217391	0.045628169	1.340766954	0.161793606	C00103 C00794 C01697	https://www.genome.jp/kegg-bin/show_pathway?hsa00052/hsa:C00103%09red/hsa:C00794%09red/hsa:C01697%09red
hsa05012	Parkinson disease	2	57	20	3250	0.1	0.046920146	1.328640647	0.161793606	C00013 C00082	https://www.genome.jp/kegg-bin/show_pathway?hsa05012/hsa:C00013%09blue/hsa:C00082%09red
hsa00053	Ascorbate and aldarate metabolism	3	57	49	3250	0.06122449	0.053391977	1.272524	0.177973256	C00818 C00879 C05422	https://www.genome.jp/kegg-bin/show_pathway?hsa00053/hsa:C00818%09red/hsa:C00879%09red/hsa:C05422%09red
hsa00220	Arginine biosynthesis	2	57	23	3250	0.086956522	0.060434458	1.218715368	0.194949865	C00064 C00624	https://www.genome.jp/kegg-bin/show_pathway?hsa00220/hsa:C00064%09red/hsa:C00624%09red
hsa04150	mTOR signaling pathway	1	57	4	3250	0.25	0.068360471	1.165194951	0.207152944	C00123	https://www.genome.jp/kegg-bin/show_pathway?hsa04150/hsa:C00123%09red
hsa04210	Apoptosis	1	57	4	3250	0.25	0.068360471	1.165194951	0.207152944	C00319	https://www.genome.jp/kegg-bin/show_pathway?hsa04210/hsa:C00319%09red
hsa00100	Steroid biosynthesis	3	57	58	3250	0.051724138	0.080176628	1.095952211	0.229211948	C00187 C01724 C05442	https://www.genome.jp/kegg-bin/show_pathway?hsa00100/hsa:C00187%09red/hsa:C01724%09red/hsa:C05442%09red
hsa04216	Ferroptosis	2	57	27	3250	0.074074074	0.080224182	1.095694703	0.229211948	C00097 C02477	https://www.genome.jp/kegg-bin/show_pathway?hsa04216/hsa:C00097%09red/hsa:C02477%09red
hsa00630	Glyoxylate and dicarboxylate metabolism	3	57	62	3250	0.048387097	0.093644117	1.028519499	0.260122548	C00037 C00064 C00065	https://www.genome.jp/kegg-bin/show_pathway?hsa00630/hsa:C00037%09red/hsa:C00064%09red/hsa:C00065%09red
hsa04916	Melanogenesis	1	57	6	3250	0.166666667	0.100797466	0.996550387	0.272425583	C00082	https://www.genome.jp/kegg-bin/show_pathway?hsa04916/hsa:C00082%09red
hsa00240	Pyrimidine metabolism	3	57	65	3250	0.046153846	0.104321863	0.981624665	0.274531219	C00064 C00106 C01013	https://www.genome.jp/kegg-bin/show_pathway?hsa00240/hsa:C00064%09red/hsa:C00106%09red/hsa:C01013%09red
hsa04979	Cholesterol metabolism	1	57	7	3250	0.142857143	0.116597264	0.933311642	0.287226589	C00187	https://www.genome.jp/kegg-bin/show_pathway?hsa04979/hsa:C00187%09red
hsa05033	Nicotine addiction	1	57	7	3250	0.142857143	0.116597264	0.933311642	0.287226589	C00334	https://www.genome.jp/kegg-bin/show_pathway?hsa05033/hsa:C00334%09red
hsa01040	Biosynthesis of unsaturated fatty acids	3	57	69	3250	0.043478261	0.119274526	0.923452301	0.287226589	C00249 C08316 C16526	https://www.genome.jp/kegg-bin/show_pathway?hsa01040/hsa:C00249%09red/hsa:C08316%09blue/hsa:C16526%09red
hsa00030	Pentose phosphate pathway	2	57	35	3250	0.057142857	0.12459359	0.9045043	0.287226589	C00121 C00257	https://www.genome.jp/kegg-bin/show_pathway?hsa00030/hsa:C00121%09red/hsa:C00257%09red
hsa04724	Glutamatergic synapse	1	57	8	3250	0.125	0.132124231	0.879017527	0.287226589	C00064	https://www.genome.jp/kegg-bin/show_pathway?hsa04724/hsa:C00064%09red
hsa05143	African trypanosomiasis	1	57	8	3250	0.125	0.132124231	0.879017527	0.287226589	C00078	https://www.genome.jp/kegg-bin/show_pathway?hsa05143/hsa:C00078%09red
hsa05030	Cocaine addiction	1	57	8	3250	0.125	0.132124231	0.879017527	0.287226589	C00082	https://www.genome.jp/kegg-bin/show_pathway?hsa05030/hsa:C00082%09red
hsa04915	Estrogen signaling pathway	1	57	8	3250	0.125	0.132124231	0.879017527	0.287226589	C00334	https://www.genome.jp/kegg-bin/show_pathway?hsa04915/hsa:C00334%09red
hsa04977	Vitamin digestion and absorption	2	57	37	3250	0.054054054	0.136462773	0.864985808	0.290346326	C00187 C02477	https://www.genome.jp/kegg-bin/show_pathway?hsa04977/hsa:C00187%09red/hsa:C02477%09red
hsa04217	Necroptosis	1	57	9	3250	0.111111111	0.147382997	0.831552617	0.294765994	C00319	https://www.genome.jp/kegg-bin/show_pathway?hsa04217/hsa:C00319%09red
hsa04929	GnRH secretion	1	57	9	3250	0.111111111	0.147382997	0.831552617	0.294765994	C00334	https://www.genome.jp/kegg-bin/show_pathway?hsa04929/hsa:C00334%09red
hsa05032	Morphine addiction	1	57	9	3250	0.111111111	0.147382997	0.831552617	0.294765994	C00334	https://www.genome.jp/kegg-bin/show_pathway?hsa05032/hsa:C00334%09red
hsa00330	Arginine and proline metabolism	3	57	78	3250	0.038461538	0.155567259	0.808081799	0.305033842	C00148 C00334 C00750	https://www.genome.jp/kegg-bin/show_pathway?hsa00330/hsa:C00148%09red/hsa:C00334%09red/hsa:C00750%09red
hsa05031	Amphetamine addiction	1	57	10	3250	0.1	0.162378112	0.789472511	0.306373797	C00082	https://www.genome.jp/kegg-bin/show_pathway?hsa05031/hsa:C00082%09red
hsa05034	Alcoholism	1	57	10	3250	0.1	0.162378112	0.789472511	0.306373797	C00082	https://www.genome.jp/kegg-bin/show_pathway?hsa05034/hsa:C00082%09red
hsa00380	Tryptophan metabolism	3	57	83	3250	0.036144578	0.177080936	0.751828192	0.322025551	C00078 C01717 C05635	https://www.genome.jp/kegg-bin/show_pathway?hsa00380/hsa:C00078%09red/hsa:C01717%09red/hsa:C05635%09red
hsa04917	Prolactin signaling pathway	1	57	11	3250	0.090909091	0.177114053	0.751746979	0.322025551	C00082	https://www.genome.jp/kegg-bin/show_pathway?hsa04917/hsa:C00082%09red
hsa04728	Dopaminergic synapse	1	57	12	3250	0.083333333	0.19159522	0.717615331	0.330336586	C00082	https://www.genome.jp/kegg-bin/show_pathway?hsa04728/hsa:C00082%09red
hsa04927	Cortisol synthesis and secretion	1	57	12	3250	0.083333333	0.19159522	0.717615331	0.330336586	C00187	https://www.genome.jp/kegg-bin/show_pathway?hsa04927/hsa:C00187%09red
hsa04975	Fat digestion and absorption	1	57	12	3250	0.083333333	0.19159522	0.717615331	0.330336586	C00187	https://www.genome.jp/kegg-bin/show_pathway?hsa04975/hsa:C00187%09red
hsa00471	D-Glutamine and D-glutamate metabolism	1	57	13	3250	0.076923077	0.20582594	0.686499892	0.343043233	C00064	https://www.genome.jp/kegg-bin/show_pathway?hsa00471/hsa:C00064%09red
hsa04934	Cushing syndrome	1	57	13	3250	0.076923077	0.20582594	0.686499892	0.343043233	C00187	https://www.genome.jp/kegg-bin/show_pathway?hsa04934/hsa:C00187%09red
hsa00190	Oxidative phosphorylation	1	57	16	3250	0.0625	0.247057622	0.607201742	0.398480036	C00013	https://www.genome.jp/kegg-bin/show_pathway?hsa00190/hsa:C00013%09blue
hsa04978	Mineral absorption	1	57	16	3250	0.0625	0.247057622	0.607201742	0.398480036	C00037	https://www.genome.jp/kegg-bin/show_pathway?hsa04978/hsa:C00037%09red
hsa04964	Proximal tubule bicarbonate reclamation	1	57	17	3250	0.058823529	0.260328406	0.58447844	0.413219693	C00064	https://www.genome.jp/kegg-bin/show_pathway?hsa04964/hsa:C00064%09red
hsa00360	Phenylalanine metabolism	2	57	60	3250	0.033333333	0.283747974	0.54706723	0.433612548	C00079 C00082	https://www.genome.jp/kegg-bin/show_pathway?hsa00360/hsa:C00079%09red/hsa:C00082%09red
hsa00910	Nitrogen metabolism	1	57	19	3250	0.052631579	0.286184282	0.543354223	0.433612548	C00064	https://www.genome.jp/kegg-bin/show_pathway?hsa00910/hsa:C00064%09red
hsa04723	Retrograde endocannabinoid signaling	1	57	19	3250	0.052631579	0.286184282	0.543354223	0.433612548	C00334	https://www.genome.jp/kegg-bin/show_pathway?hsa04723/hsa:C00334%09red
hsa04931	Insulin resistance	1	57	20	3250	0.05	0.298777131	0.524652647	0.439378134	C00043	https://www.genome.jp/kegg-bin/show_pathway?hsa04931/hsa:C00043%09red
hsa04913	Ovarian steroidogenesis	1	57	20	3250	0.05	0.298777131	0.524652647	0.439378134	C00187	https://www.genome.jp/kegg-bin/show_pathway?hsa04913/hsa:C00187%09red
hsa04925	Aldosterone synthesis and secretion	1	57	21	3250	0.047619048	0.311151652	0.507027889	0.450944423	C00187	https://www.genome.jp/kegg-bin/show_pathway?hsa04925/hsa:C00187%09red
hsa04976	Bile secretion	1	57	22	3250	0.045454545	0.323311564	0.490378761	0.461873663	C00187	https://www.genome.jp/kegg-bin/show_pathway?hsa04976/hsa:C00187%09red
hsa05200	Pathways in cancer	1	57	24	3250	0.041666667	0.347002126	0.459667865	0.478053208	C00187	https://www.genome.jp/kegg-bin/show_pathway?hsa05200/hsa:C00187%09red
hsa00130	Ubiquinone and other terpenoid-quinone biosynthesis	2	57	71	3250	0.028169014	0.355332334	0.449365271	0.478053208	C00082 C02477	https://www.genome.jp/kegg-bin/show_pathway?hsa00130/hsa:C00082%09red/hsa:C02477%09red
hsa04922	Glucagon signaling pathway	1	57	25	3250	0.04	0.358539906	0.4454625	0.478053208	C00103	https://www.genome.jp/kegg-bin/show_pathway?hsa04922/hsa:C00103%09red
hsa04024	cAMP signaling pathway	1	57	25	3250	0.04	0.358539906	0.4454625	0.478053208	C00334	https://www.genome.jp/kegg-bin/show_pathway?hsa04024/hsa:C00334%09red
hsa04742	Taste transduction	1	57	25	3250	0.04	0.358539906	0.4454625	0.478053208	C00334	https://www.genome.jp/kegg-bin/show_pathway?hsa04742/hsa:C00334%09red
hsa04973	Carbohydrate digestion and absorption	1	57	26	3250	0.038461538	0.36987734	0.431942274	0.486680711	C01835	https://www.genome.jp/kegg-bin/show_pathway?hsa04973/hsa:C01835%09red
hsa00450	Selenocompound metabolism	1	57	27	3250	0.037037037	0.381017846	0.419054682	0.494828372	C00041	https://www.genome.jp/kegg-bin/show_pathway?hsa00450/hsa:C00041%09red
hsa01523	Antifolate resistance	1	57	28	3250	0.035714286	0.391964785	0.40675295	0.502518955	C00073	https://www.genome.jp/kegg-bin/show_pathway?hsa01523/hsa:C00073%09red
hsa00750	Vitamin B6 metabolism	1	57	29	3250	0.034482759	0.40272146	0.394995228	0.503401825	C00064	https://www.genome.jp/kegg-bin/show_pathway?hsa00750/hsa:C00064%09red
hsa04070	Phosphatidylinositol signaling system	1	57	29	3250	0.034482759	0.40272146	0.394995228	0.503401825	C03546	https://www.genome.jp/kegg-bin/show_pathway?hsa04070/hsa:C03546%09red
hsa00010	Glycolysis / Gluconeogenesis	1	57	31	3250	0.032258065	0.423676961	0.372965152	0.523057976	C00103	https://www.genome.jp/kegg-bin/show_pathway?hsa00010/hsa:C00103%09red
hsa00500	Starch and sucrose metabolism	1	57	37	3250	0.027027027	0.482304021	0.316679117	0.585104964	C00103	https://www.genome.jp/kegg-bin/show_pathway?hsa00500/hsa:C00103%09red
hsa00561	Glycerolipid metabolism	1	57	38	3250	0.026315789	0.49148817	0.308486931	0.585104964	C00103	https://www.genome.jp/kegg-bin/show_pathway?hsa00561/hsa:C00103%09red
hsa00062	Fatty acid elongation	1	57	38	3250	0.026315789	0.49148817	0.308486931	0.585104964	C00249	https://www.genome.jp/kegg-bin/show_pathway?hsa00062/hsa:C00249%09red
hsa00140	Steroid hormone biosynthesis	2	57	99	3250	0.02020202	0.523373543	0.281188235	0.612384583	C00187 C05139	https://www.genome.jp/kegg-bin/show_pathway?hsa00140/hsa:C00187%09red/hsa:C05139%09blue
hsa00650	Butanoate metabolism	1	57	42	3250	0.023809524	0.526650741	0.2784773	0.612384583	C00334	https://www.genome.jp/kegg-bin/show_pathway?hsa00650/hsa:C00334%09red
hsa00340	Histidine metabolism	1	57	47	3250	0.021276596	0.567259635	0.246218118	0.638845115	C00135	https://www.genome.jp/kegg-bin/show_pathway?hsa00340/hsa:C00135%09red
hsa00562	Inositol phosphate metabolism	1	57	47	3250	0.021276596	0.567259635	0.246218118	0.638845115	C03546	https://www.genome.jp/kegg-bin/show_pathway?hsa00562/hsa:C03546%09red
hsa00520	Amino sugar and nucleotide sugar metabolism	2	57	108	3250	0.018518519	0.57110362	0.243285087	0.638845115	C00043 C00103	https://www.genome.jp/kegg-bin/show_pathway?hsa00520/hsa:C00043%09red/hsa:C00103%09red
hsa00640	Propanoate metabolism	1	57	48	3250	0.020833333	0.574960604	0.240361912	0.638845115	C01013	https://www.genome.jp/kegg-bin/show_pathway?hsa00640/hsa:C01013%09red
hsa00310	Lysine degradation	1	57	50	3250	0.02	0.589960806	0.22917684	0.641261745	C00037	https://www.genome.jp/kegg-bin/show_pathway?hsa00310/hsa:C00037%09red
hsa00071	Fatty acid degradation	1	57	50	3250	0.02	0.589960806	0.22917684	0.641261745	C00249	https://www.genome.jp/kegg-bin/show_pathway?hsa00071/hsa:C00249%09red
hsa00564	Glycerophospholipid metabolism	1	57	52	3250	0.019230769	0.604440595	0.218646376	0.649936124	C04308	https://www.genome.jp/kegg-bin/show_pathway?hsa00564/hsa:C04308%09red
hsa00440	Phosphonate and phosphinate metabolism	1	57	53	3250	0.018867925	0.611490903	0.213609999	0.650522238	C00037	https://www.genome.jp/kegg-bin/show_pathway?hsa00440/hsa:C00037%09red
hsa00051	Fructose and mannose metabolism	1	57	54	3250	0.018518519	0.618417715	0.208718078	0.650966016	C00794	https://www.genome.jp/kegg-bin/show_pathway?hsa00051/hsa:C00794%09red
hsa00760	Nicotinate and nicotinamide metabolism	1	57	55	3250	0.018181818	0.625223157	0.203964945	0.651274122	C00334	https://www.genome.jp/kegg-bin/show_pathway?hsa00760/hsa:C00334%09red
hsa00061	Fatty acid biosynthesis	1	57	56	3250	0.017857143	0.631909316	0.199345242	0.651452903	C00249	https://www.genome.jp/kegg-bin/show_pathway?hsa00061/hsa:C00249%09red
hsa00860	Porphyrin and chlorophyll metabolism	2	57	138	3250	0.014492754	0.705230332	0.151669017	0.719622788	C00037 C00188	https://www.genome.jp/kegg-bin/show_pathway?hsa00860/hsa:C00037%09red/hsa:C00188%09red
hsa00350	Tyrosine metabolism	1	57	78	3250	0.012820513	0.75263807	0.123413818	0.760240474	C00082	https://www.genome.jp/kegg-bin/show_pathway?hsa00350/hsa:C00082%09red
hsa00524	Neomycin, kanamycin and gentamicin biosynthesis	1	57	81	3250	0.012345679	0.765739019	0.115919223	0.765739019	C00043	https://www.genome.jp/kegg-bin/show_pathway?hsa00524/hsa:C00043%09red

Supplement 5A: Metabolic bubble map analysis evidence for metabolic pathways of Group1 and Group4. Metabolic pathway analysis was performed on male samples from the control group and the experimental group. P-value is the significance of the metabolic signaling pathway.
