# Supplementary material for: Study on the Metabonomics Mechanism of Mongolian Medical Andai Therapy on Healthy People
Source: Evid Based Complement Alternat Med. 2022 Jun 20;2022:1364408. doi: 10.1155/2022/1364408 (PMC9236767; doi:10.1155/2022/1364408)
Supplement: Supplementary Materials — Supplement 1 is evidence for the principal component analysis (PCA) diagram. Supplement 2A is evidence for (group 1-group 4) female sample comparison volcanic map analysis representing metabolites, as shown in Figure 6. Supplement 2B is evidence for (group 2-group 3) male sample comparison volcanic map analysis representing metabolites, as shown in Figure 7. Supplement2C is evidence for (group 1-group 2 and group 3-group 4) full-sample comparison volcanic map analysis representing metabolites, as shown in Figure 5. Supplement 3A is evidence for (group 1A and group 4A) female sample clustering heat map analysis, as shown in Figure 9. Supplement 3B is evidence for (group 2A and group 3A) male sample clustering heat map analysis, as shown in Figure 10. Supplement 3C is evidence for (group 1A-2A and group 3A-4A) whole-sample clustering heat map analysis， as shown in Figure 8. Supplement 4A is evidence for (group 1A and group 4A) accumulation of metabolic pathways in female samples—Top20, as shown in Figure 13. Supplement 4B is evidence for (group 2 and group 3) accumulation of metabolic pathways in male samples—Top20, as shown in Figure 15. Supplement 4C is evidence for (group 1-2 and group 3-4) enrichment of metabolic pathways in the whole sample—Top20, as shown in Figure 11. Supplement 5A is evidence for (group 1 and group 4) metabolic bubble of female sample, as shown in Figure 14. Supplement 5B is evidence for (group 2 and group 3) metabolic bubble of male sample, as shown in Figure 16. Supplement 5C is evidence for metabolic pathways of (group 1-2 and group 3-4) metabolic bubble of the whole sample, as shown in Figure 12. [file 1364408.f1.zip › 1364408.f1/Supplement 5B.pdf]

Supplement 5B

ID	Annotation	in set	set	in background	background	RichFactor	p-value	-lg(p-value)	FDR correction	Matching IDs	URL
hsa00040	Pentose and glucuronate interconversions	3	17	56	3250	0.053571429	0.002777169	2.556397652	0.090659532	C00103 C00181 C00310	https://www.genome.jp/kegg -bin/show_pathway?hsa00040/hsa:C00103%09red/hsa:C00181%09red/hsa:C00310%09red
hsa04742	Taste transduction	2	17	25	3250	0.08	0.007199629	2.142689909	0.090659532	C00020 C00158	https://www.genome.jp/kegg -bin/show_pathway?hsa04742/hsa:C00020%09red/hsa:C00158%09red
hsa04922	Glucagon signaling pathway	2	17	25	3250	0.08	0.007199629	2.142689909	0.090659532	C00103 C00158	https://www.genome.jp/kegg -bin/show_pathway?hsa04922/hsa:C00103%09red/hsa:C00158%09red
hsa00380	Tryptophan metabolism	3	17	83	3250	0.036144578	0.008433445	2.073994991	0.090659532	C01717 C05635 C10164	https://www.genome.jp/kegg -bin/show_pathway?hsa00380/hsa:C01717%09red/hsa:C05635%09red/hsa:C10164%09blue
hsa00030	Pentose phosphate pathway	2	17	35	3250	0.057142857	0.013847587	1.858625885	0.119089252	C00121 C00257	https://www.genome.jp/kegg -bin/show_pathway?hsa00030/hsa:C00121%09red/hsa:C00257%09red
hsa04150	mTOR signaling pathway	1	17	4	3250	0.25	0.020768996	1.682584501	0.127580974	C00020	https://www.genome.jp/kegg -bin/show_pathway?hsa04150/hsa:C00020%09red
hsa04151	PI3K-Akt signaling pathway	1	17	4	3250	0.25	0.020768996	1.682584501	0.127580974	C00020	https://www.genome.jp/kegg -bin/show_pathway?hsa04151/hsa:C00020%09red
hsa04068	FoxO signaling pathway	1	17	5	3250	0.2	0.025897439	1.586743178	0.139198736	C00020	https://www.genome.jp/kegg -bin/show_pathway?hsa04068/hsa:C00020%09red
hsa04211	Longevity regulating pathway	1	17	8	3250	0.125	0.041131517	1.385825277	0.15714254	C00020	https://www.genome.jp/kegg -bin/show_pathway?hsa04211/hsa:C00020%09red
hsa04740	Olfactory transduction	1	17	8	3250	0.125	0.041131517	1.385825277	0.15714254	C00020	https://www.genome.jp/kegg -bin/show_pathway?hsa04740/hsa:C00020%09red
hsa00240	Pyrimidine metabolism	2	17	65	3250	0.030769231	0.044163956	1.354932033	0.15714254	C00106 C00295	https://www.genome.jp/kegg -bin/show_pathway?hsa00240/hsa:C00106%09red/hsa:C00295%09red
hsa05032	Morphine addiction	1	17	9	3250	0.111111111	0.046159513	1.335738782	0.15714254	C00020	https://www.genome.jp/kegg -bin/show_pathway?hsa05032/hsa:C00020%09red
hsa04022	cGMP-PKG signaling pathway	1	17	10	3250	0.1	0.051162687	1.291046652	0.15714254	C00020	https://www.genome.jp/kegg -bin/show_pathway?hsa04022/hsa:C00020%09red
hsa04928	Parathyroid hormone synthesis, secretion and action	1	17	10	3250	0.1	0.051162687	1.291046652	0.15714254	C00020	https://www.genome.jp/kegg -bin/show_pathway?hsa04928/hsa:C00020%09red
hsa04927	Cortisol synthesis and secretion	1	17	12	3250	0.083333333	0.06109503	1.213994121	0.174079933	C00020	https://www.genome.jp/kegg -bin/show_pathway?hsa04927/hsa:C00020%09red
hsa04934	Cushing syndrome	1	17	13	3250	0.076923077	0.066024426	1.180295368	0.174079933	C00020	https://www.genome.jp/kegg -bin/show_pathway?hsa04934/hsa:C00020%09red
hsa04923	Regulation of lipolysis in adipocytes	1	17	14	3250	0.071428571	0.070929456	1.149173368	0.174079933	C00020	https://www.genome.jp/kegg -bin/show_pathway?hsa04923/hsa:C00020%09red
hsa02010	ABC transporters	2	17	93	3250	0.021505376	0.083417432	1.078743182	0.174079933	C00121 C00181	https://www.genome.jp/kegg -bin/show_pathway?hsa02010/hsa:C00121%09red/hsa:C00181%09red
hsa04924	Renin secretion	1	17	17	3250	0.058823529	0.085499484	1.068036505	0.174079933	C00020	https://www.genome.jp/kegg -bin/show_pathway?hsa04924/hsa:C00020%09red
hsa04726	Serotonergic synapse	1	17	17	3250	0.058823529	0.085499484	1.068036505	0.174079933	C05635	https://www.genome.jp/kegg -bin/show_pathway?hsa04726/hsa:C05635%09red
hsa00230	Purine metabolism	2	17	95	3250	0.021052632	0.086537347	1.062796423	0.174079933	C00020 C01762	https://www.genome.jp/kegg -bin/show_pathway?hsa00230/hsa:C00020%09red/hsa:C01762%09red
hsa05012	Parkinson disease	1	17	20	3250	0.05	0.099854262	1.000633396	0.174079933	C00020	https://www.genome.jp/kegg -bin/show_pathway?hsa05012/hsa:C00020%09red
hsa00020	Citrate cycle (TCA cycle)	1	17	20	3250	0.05	0.099854262	1.000633396	0.174079933	C00158	https://www.genome.jp/kegg -bin/show_pathway?hsa00020/hsa:C00158%09red
hsa04925	Aldosterone synthesis and secretion	1	17	21	3250	0.047619048	0.104591871	0.980502069	0.174079933	C00020	https://www.genome.jp/kegg -bin/show_pathway?hsa04925/hsa:C00020%09red
hsa00520	Amino sugar and nucleotide sugar metabolism	2	17	108	3250	0.018518519	0.107663037	0.967933372	0.174079933	C00103 C00181	https://www.genome.jp/kegg -bin/show_pathway?hsa00520/hsa:C00103%09red/hsa:C00181%09red
hsa04152	AMPK signaling pathway	1	17	22	3250	0.045454545	0.109306005	0.96135598	0.174079933	C00020	https://www.genome.jp/kegg -bin/show_pathway?hsa04152/hsa:C00020%09red
hsa00232	Caffeine metabolism	1	17	22	3250	0.045454545	0.109306005	0.96135598	0.174079933	C01762	https://www.genome.jp/kegg -bin/show_pathway?hsa00232/hsa:C01762%09red
hsa04024	cAMP signaling pathway	1	17	25	3250	0.04	0.123308643	0.909006482	0.189366844	C00020	https://www.genome.jp/kegg -bin/show_pathway?hsa04024/hsa:C00020%09red
hsa01523	Antifolate resistance	1	17	28	3250	0.035714286	0.137103902	0.862950184	0.196257018	C00020	https://www.genome.jp/kegg -bin/show_pathway?hsa01523/hsa:C00020%09red
hsa00250	Alanine, aspartate and glutamate metabolism	1	17	28	3250	0.035714286	0.137103902	0.862950184	0.196257018	C00158	https://www.genome.jp/kegg -bin/show_pathway?hsa00250/hsa:C00158%09red
hsa00770	Pantothenate and CoA biosynthesis	1	17	30	3250	0.033333333	0.146186956	0.835091377	0.196257018	C00106	https://www.genome.jp/kegg -bin/show_pathway?hsa00770/hsa:C00106%09red
hsa05230	Central carbon metabolism in cancer	1	17	30	3250	0.033333333	0.146186956	0.835091377	0.196257018	C00158	https://www.genome.jp/kegg -bin/show_pathway?hsa05230/hsa:C00158%09red
hsa00010	Glycolysis / Gluconeogenesis	1	17	31	3250	0.032258065	0.150694665	0.821902124	0.196257018	C00103	https://www.genome.jp/kegg -bin/show_pathway?hsa00010/hsa:C00103%09red
hsa00410	beta-Alanine metabolism	1	17	32	3250	0.03125	0.155179968	0.809164343	0.196257018	C00106	https://www.genome.jp/kegg -bin/show_pathway?hsa00410/hsa:C00106%09red
hsa00400	Phenylalanine, tyrosine and tryptophan biosynthesis	1	17	34	3250	0.029411765	0.164083775	0.784934362	0.201588637	C01094	https://www.genome.jp/kegg -bin/show_pathway?hsa00400/hsa:C01094%09red
hsa00500	Starch and sucrose metabolism	1	17	37	3250	0.027027027	0.17727404	0.751354858	0.211080129	C00103	https://www.genome.jp/kegg -bin/show_pathway?hsa00500/hsa:C00103%09red
hsa00561	Glycerolipid metabolism	1	17	38	3250	0.026315789	0.181627087	0.740819381	0.211080129	C00103	https://www.genome.jp/kegg -bin/show_pathway?hsa00561/hsa:C00103%09red
hsa00052	Galactose metabolism	1	17	46	3250	0.02173913	0.215679299	0.666191537	0.242388575	C00103	https://www.genome.jp/kegg -bin/show_pathway?hsa00052/hsa:C00103%09red
hsa00120	Primary bile acid biosynthesis	1	17	47	3250	0.021276596	0.219840801	0.657891703	0.242388575	C02528	https://www.genome.jp/kegg -bin/show_pathway?hsa00120/hsa:C02528%09red
hsa00564	Glycerophospholipid metabolism	1	17	52	3250	0.019230769	0.240338431	0.619176779	0.258363813	C04308	https://www.genome.jp/kegg -bin/show_pathway?hsa00564/hsa:C04308%09red
hsa00051	Fructose and mannose metabolism	1	17	54	3250	0.018518519	0.248394673	0.604857723	0.260511486	C01094	https://www.genome.jp/kegg -bin/show_pathway?hsa00051/hsa:C01094%09red
hsa00630	Glyoxylate and dicarboxylate metabolism	1	17	62	3250	0.016129032	0.279822533	0.553117317	0.286484974	C00158	https://www.genome.jp/kegg -bin/show_pathway?hsa00630/hsa:C00158%09red
hsa01040	Biosynthesis of unsaturated fatty acids	1	17	69	3250	0.014492754	0.306303224	0.513848433	0.306303224	C08281	https://www.genome.jp/kegg -bin/show_pathway?hsa01040/hsa:C08281%09red

Supplement 5B: Metabolic bubble map analysis evidence for metabolic pathways of Group2 and Group3. Metabolic pathway analysis was performed on control and experimental female samples. P-value is the significance of the metabolic signaling pathway.
