# Supplementary material for: Study on the Metabonomics Mechanism of Mongolian Medical Andai Therapy on Healthy People
Source: Evid Based Complement Alternat Med. 2022 Jun 20;2022:1364408. doi: 10.1155/2022/1364408 (PMC9236767; doi:10.1155/2022/1364408)
Supplement: Supplementary Materials — Supplement 1 is evidence for the principal component analysis (PCA) diagram. Supplement 2A is evidence for (group 1-group 4) female sample comparison volcanic map analysis representing metabolites, as shown in Figure 6. Supplement 2B is evidence for (group 2-group 3) male sample comparison volcanic map analysis representing metabolites, as shown in Figure 7. Supplement2C is evidence for (group 1-group 2 and group 3-group 4) full-sample comparison volcanic map analysis representing metabolites, as shown in Figure 5. Supplement 3A is evidence for (group 1A and group 4A) female sample clustering heat map analysis, as shown in Figure 9. Supplement 3B is evidence for (group 2A and group 3A) male sample clustering heat map analysis, as shown in Figure 10. Supplement 3C is evidence for (group 1A-2A and group 3A-4A) whole-sample clustering heat map analysis， as shown in Figure 8. Supplement 4A is evidence for (group 1A and group 4A) accumulation of metabolic pathways in female samples—Top20, as shown in Figure 13. Supplement 4B is evidence for (group 2 and group 3) accumulation of metabolic pathways in male samples—Top20, as shown in Figure 15. Supplement 4C is evidence for (group 1-2 and group 3-4) enrichment of metabolic pathways in the whole sample—Top20, as shown in Figure 11. Supplement 5A is evidence for (group 1 and group 4) metabolic bubble of female sample, as shown in Figure 14. Supplement 5B is evidence for (group 2 and group 3) metabolic bubble of male sample, as shown in Figure 16. Supplement 5C is evidence for metabolic pathways of (group 1-2 and group 3-4) metabolic bubble of the whole sample, as shown in Figure 12. [file 1364408.f1.zip › 1364408.f1/Supplement 5C.pdf]

Supplement 5C

ID Annotation	Annotation	in set	set	in background	background	RichFactor	p-value	-lg(p-value)	FDR correction	Matching IDs	URL
hsa00970	Aminoacyl-tRNA biosynthesis	7	53	52	3250	0.134615385	1.5558E-05	4.808045171	0.001038689	C00041 C00073 C00078 C00079 C00082 C00123 C00407	https://www.genome.jp/kegg-bin/show_pathway?hsa00970/hsa:C00041%09red/hsa:C00073%09red/hsa:C00078%09red/hsa:C00079%09red/hsa:C00082%09red/hsa:C00123%09red/hsa:C00407%09red
hsa00290	Valine, leucine and isoleucine biosynthesis	5	53	23	3250	0.217391304	2.56466E-05	4.590969555	0.001038689	C00109 C00123 C00141 C00233 C00407	https://www.genome.jp/kegg-bin/show_pathway?hsa00290/hsa:C00109%09red/hsa:C00123%09red/hsa:C00141%09red/hsa:C00233%09red/hsa:C00407%09red
hsa02010	ABC transporters	8	53	93	3250	0.086021505	0.000102166	3.990692871	0.002758487	C00041 C00059 C00116 C00121 C00245 C00503 C00794 C02273	https://www.genome.jp/kegg-bin/show_pathway?hsa02010/hsa:C00041%09red/hsa:C00059%09red/hsa:C00116%09red/hsa:C00121%09red/hsa:C00245%09red/hsa:C00503%09red/hsa:C00794%09red/hsa:C02273%09red
hsa00400	Phenylalanine, tyrosine and tryptophan biosynthesis	5	53	34	3250	0.147058824	0.000185096	3.732603312	0.003748191	C00078 C00079 C00082 C00296 C00587	https://www.genome.jp/kegg-bin/show_pathway?hsa00400/hsa:C00078%09red/hsa:C00079%09red/hsa:C00082%09red/hsa:C00296%09red/hsa:C00587%09red
hsa00040	Pentose and glucuronate interconversions	6	53	56	3250	0.107142857	0.000244972	3.610884194	0.003968541	C00103 C00116 C00310 C00532 C02266 C02273	https://www.genome.jp/kegg-bin/show_pathway?hsa00040/hsa:C00103%09red/hsa:C00116%09red/hsa:C00310%09red/hsa:C00532%09red/hsa:C02266%09red/hsa:C02273%09red
hsa00280	Valine, leucine and isoleucine degradation	4	53	41	3250	0.097560976	0.00408609	2.388692086	0.055162213	C00123 C00141 C00233 C00407	https://www.genome.jp/kegg-bin/show_pathway?hsa00280/hsa:C00123%09red/hsa:C00141%09red/hsa:C00233%09red/hsa:C00407%09red
hsa00052	Galactose metabolism	4	53	46	3250	0.086956522	0.00620047	2.207575394	0.069479068	C00103 C00116 C00794 C01697	https://www.genome.jp/kegg-bin/show_pathway?hsa00052/hsa:C00103%09red/hsa:C00116%09red/hsa:C00794%09red/hsa:C01697%09red
hsa05143	African trypanosomiasis	2	53	8	3250	0.25	0.00686213	2.163541046	0.069479068	C00078 C00328	https://www.genome.jp/kegg-bin/show_pathway?hsa05143/hsa:C00078%09red/hsa:C00328%09red
hsa00250	Alanine, aspartate and glutamate metabolism	3	53	28	3250	0.107142857	0.0100616	1.997332938	0.082264101	C00041 C00158 C00438	https://www.genome.jp/kegg-bin/show_pathway?hsa00250/hsa:C00041%09red/hsa:C00158%09red/hsa:C00438%09red
hsa00380	Tryptophan metabolism	5	53	83	3250	0.060240964	0.010562127	1.976248624	0.082264101	C00078 C00328 C01717 C05635 C10164	https://www.genome.jp/kegg-bin/show_pathway?hsa00380/hsa:C00078%09red/hsa:C00328%09red/hsa:C01717%09red/hsa:C05635%09red/hsa:C10164%09blue
hsa05230	Central carbon metabolism in cancer	3	53	30	3250	0.1	0.012187274	1.914093416	0.082264101	C00041 C00123 C00158	https://www.genome.jp/kegg-bin/show_pathway?hsa05230/hsa:C00041%09red/hsa:C00123%09red/hsa:C00158%09red
hsa00770	Pantothenate and CoA biosynthesis	3	53	30	3250	0.1	0.012187274	1.914093416	0.082264101	C00106 C00141 C00750	https://www.genome.jp/kegg-bin/show_pathway?hsa00770/hsa:C00106%09red/hsa:C00141%09red/hsa:C00750%09red
hsa00410	beta-Alanine metabolism	3	53	32	3250	0.09375	0.014552311	1.837068031	0.090672092	C00106 C00750 C01013	https://www.genome.jp/kegg-bin/show_pathway?hsa00410/hsa:C00106%09red/hsa:C00750%09red/hsa:C01013%09red
hsa05217	Basal cell carcinoma	1	53	1	3250	1	0.016307692	1.787607491	0.094351648	C00187	https://www.genome.jp/kegg-bin/show_pathway?hsa05217/hsa:C00187%09red
hsa00270	Cysteine and methionine metabolism	4	53	63	3250	0.063492063	0.018467404	1.733594139	0.099723984	C00041 C00059 C00073 C00109	https://www.genome.jp/kegg-bin/show_pathway?hsa00270/hsa:C00041%09red/hsa:C00059%09red/hsa:C00073%09red/hsa:C00109%09red
hsa00240	Pyrimidine metabolism	4	53	65	3250	0.061538462	0.020495638	1.68833856	0.099819935	C00106 C00438 C01013 C01563	https://www.genome.jp/kegg-bin/show_pathway?hsa00240/hsa:C00106%09red/hsa:C00438%09red/hsa:C01013%09red/hsa:C01563%09red
hsa05131	Shigellosis	2	53	14	3250	0.142857143	0.020949863	1.678818815	0.099819935	C00123 C00407	https://www.genome.jp/kegg-bin/show_pathway?hsa05131/hsa:C00123%09red/hsa:C00407%09red
hsa04726	Serotonergic synapse	2	53	17	3250	0.117647059	0.03035068	1.517831573	0.136578061	C00078 C05635	https://www.genome.jp/kegg-bin/show_pathway?hsa04726/hsa:C00078%09red/hsa:C05635%09red
hsa00120	Primary bile acid biosynthesis	3	53	47	3250	0.063829787	0.040114053	1.396703461	0.16648511	C00187 C00245 C02528	https://www.genome.jp/kegg-bin/show_pathway?hsa00120/hsa:C00187%09red/hsa:C00245%09red/hsa:C02528%09red
hsa00020	Citrate cycle (TCA cycle)	2	53	20	3250	0.1	0.041107435	1.386079626	0.16648511	C00158 C00417	https://www.genome.jp/kegg-bin/show_pathway?hsa00020/hsa:C00158%09red/hsa:C00417%09red
hsa00053	Ascorbate and aldarate metabolism	3	53	49	3250	0.06122449	0.044559798	1.351056783	0.171873508	C00818 C00879 C05422	https://www.genome.jp/kegg-bin/show_pathway?hsa00053/hsa:C00818%09red/hsa:C00879%09red/hsa:C05422%09red
hsa00430	Taurine and hypotaurine metabolism	2	53	22	3250	0.090909091	0.048958741	1.310169763	0.180257181	C00041 C00245	https://www.genome.jp/kegg-bin/show_pathway?hsa00430/hsa:C00041%09red/hsa:C00245%09red
hsa04922	Glucagon signaling pathway	2	53	25	3250	0.08	0.061653406	1.210042924	0.214923641	C00103 C00158	https://www.genome.jp/kegg-bin/show_pathway?hsa04922/hsa:C00103%09red/hsa:C00158%09red
hsa04150	mTOR signaling pathway	1	53	4	3250	0.25	0.063681079	1.195989587	0.214923641	C00123	https://www.genome.jp/kegg-bin/show_pathway?hsa04150/hsa:C00123%09red
hsa04974	Protein digestion and absorption	2	53	29	3250	0.068965517	0.080093019	1.096405338	0.25950138	C00041 C00079	https://www.genome.jp/kegg-bin/show_pathway?hsa04974/hsa:C00041%09red/hsa:C00079%09red
hsa04916	Melanogenesis	1	53	6	3250	0.166666667	0.094012127	1.026816119	0.292883936	C00082	https://www.genome.jp/kegg-bin/show_pathway?hsa04916/hsa:C00082%09red
hsa00920	Sulfur metabolism	2	53	33	3250	0.060606061	0.100005372	0.999976671	0.300016116	C00059 C00245	https://www.genome.jp/kegg-bin/show_pathway?hsa00920/hsa:C00059%09red/hsa:C00245%09red
hsa04979	Cholesterol metabolism	1	53	7	3250	0.142857143	0.108814026	0.963315123	0.308451671	C00187	https://www.genome.jp/kegg-bin/show_pathway?hsa04979/hsa:C00187%09red
hsa00030	Pentose phosphate pathway	2	53	35	3250	0.057142857	0.110433314	0.956899894	0.308451671	C00121 C00257	https://www.genome.jp/kegg-bin/show_pathway?hsa00030/hsa:C00121%09red/hsa:C00257%09red
hsa04977	Vitamin digestion and absorption	2	53	37	3250	0.054054054	0.121136081	0.91672648	0.310700691	C00187 C02477	https://www.genome.jp/kegg-bin/show_pathway?hsa04977/hsa:C00187%09red/hsa:C02477%09red
hsa05030	Cocaine addiction	1	53	8	3250	0.125	0.123378582	0.908760225	0.310700691	C00082	https://www.genome.jp/kegg-bin/show_pathway?hsa05030/hsa:C00082%09red
hsa00561	Glycerolipid metabolism	2	53	38	3250	0.052631579	0.126581763	0.89762886	0.310700691	C00103 C00116	https://www.genome.jp/kegg-bin/show_pathway?hsa00561/hsa:C00103%09red/hsa:C00116%09red
hsa00480	Glutathione metabolism	2	53	38	3250	0.052631579	0.126581763	0.89762886	0.310700691	C00750 C05422	https://www.genome.jp/kegg-bin/show_pathway?hsa00480/hsa:C00750%09red/hsa:C05422%09red
hsa05031	Amphetamine addiction	1	53	10	3250	0.1	0.151810547	0.818698054	0.351332981	C00082	https://www.genome.jp/kegg-bin/show_pathway?hsa05031/hsa:C00082%09red
hsa05034	Alcoholism	1	53	10	3250	0.1	0.151810547	0.818698054	0.351332981	C00082	https://www.genome.jp/kegg-bin/show_pathway?hsa05034/hsa:C00082%09red
hsa04122	Sulfur relay system	1	53	11	3250	0.090909091	0.165685251	0.78071615	0.362716361	C00041	https://www.genome.jp/kegg-bin/show_pathway?hsa04122/hsa:C00041%09red
hsa04917	Prolactin signaling pathway	1	53	11	3250	0.090909091	0.165685251	0.78071615	0.362716361	C00082	https://www.genome.jp/kegg-bin/show_pathway?hsa04917/hsa:C00082%09red
hsa04728	Dopaminergic synapse	1	53	12	3250	0.083333333	0.179337206	0.746329601	0.362998848	C00082	https://www.genome.jp/kegg-bin/show_pathway?hsa04728/hsa:C00082%09red
hsa04927	Cortisol synthesis and secretion	1	53	12	3250	0.083333333	0.179337206	0.746329601	0.362998848	C00187	https://www.genome.jp/kegg-bin/show_pathway?hsa04927/hsa:C00187%09red
hsa04975	Fat digestion and absorption	1	53	12	3250	0.083333333	0.179337206	0.746329601	0.362998848	C00187	https://www.genome.jp/kegg-bin/show_pathway?hsa04975/hsa:C00187%09red
hsa00640	Propanoate metabolism	2	53	48	3250	0.041666667	0.183740158	0.735795915	0.362998848	C00109 C01013	https://www.genome.jp/kegg-bin/show_pathway?hsa00640/hsa:C00109%09red/hsa:C01013%09red
hsa04934	Cushing syndrome	1	53	13	3250	0.076923077	0.19276992	0.714960733	0.368479086	C00187	https://www.genome.jp/kegg-bin/show_pathway?hsa04934/hsa:C00187%09red
hsa00260	Glycine, serine and threonine metabolism	2	53	50	3250	0.04	0.195612354	0.70860372	0.368479086	C00078 C00109	https://www.genome.jp/kegg-bin/show_pathway?hsa00260/hsa:C00078%09red/hsa:C00109%09red
hsa00230	Purine metabolism	3	53	95	3250	0.031578947	0.20084258	0.697144207	0.369732932	C00059 C00147 C00387	https://www.genome.jp/kegg-bin/show_pathway?hsa00230/hsa:C00059%09red/hsa:C00147%09red/hsa:C00387%09red
hsa04923	Regulation of lipolysis in adipocytes	1	53	14	3250	0.071428571	0.205986848	0.686160509	0.370776326	C00116	https://www.genome.jp/kegg-bin/show_pathway?hsa04923/hsa:C00116%09red
hsa00100	Steroid biosynthesis	2	53	58	3250	0.034482759	0.243909951	0.612770481	0.429366796	C00187 C05442	https://www.genome.jp/kegg-bin/show_pathway?hsa00100/hsa:C00187%09red/hsa:C05442%09red
hsa00360	Phenylalanine metabolism	2	53	60	3250	0.033333333	0.256102375	0.591586393	0.429366796	C00079 C00082	https://www.genome.jp/kegg-bin/show_pathway?hsa00360/hsa:C00079%09red/hsa:C00082%09red
hsa00630	Glyoxylate and dicarboxylate metabolism	2	53	62	3250	0.032258065	0.268311706	0.57136038	0.429366796	C00158 C00417	https://www.genome.jp/kegg-bin/show_pathway?hsa00630/hsa:C00158%09red/hsa:C00417%09red
hsa04723	Retrograde endocannabinoid signaling	1	53	19	3250	0.052631579	0.268951893	0.570325395	0.429366796	C00116	https://www.genome.jp/kegg-bin/show_pathway?hsa04723/hsa:C00116%09red
hsa00910	Nitrogen metabolism	1	53	19	3250	0.052631579	0.268951893	0.570325395	0.429366796	C01563	https://www.genome.jp/kegg-bin/show_pathway?hsa00910/hsa:C01563%09red
hsa04931	Insulin resistance	1	53	20	3250	0.05	0.280943706	0.551380693	0.429366796	C00043	https://www.genome.jp/kegg-bin/show_pathway?hsa04931/hsa:C00043%09red
hsa05012	Parkinson disease	1	53	20	3250	0.05	0.280943706	0.551380693	0.429366796	C00082	https://www.genome.jp/kegg-bin/show_pathway?hsa05012/hsa:C00082%09red
hsa04913	Ovarian steroidogenesis	1	53	20	3250	0.05	0.280943706	0.551380693	0.429366796	C00187	https://www.genome.jp/kegg-bin/show_pathway?hsa04913/hsa:C00187%09red
hsa04925	Aldosterone synthesis and secretion	1	53	21	3250	0.047619048	0.292742463	0.533514278	0.439113694	C00187	https://www.genome.jp/kegg-bin/show_pathway?hsa04925/hsa:C00187%09red
hsa04976	Bile secretion	1	53	22	3250	0.045454545	0.304351211	0.516624965	0.440993264	C00187	https://www.genome.jp/kegg-bin/show_pathway?hsa04976/hsa:C00187%09red
hsa01040	Biosynthesis of unsaturated fatty acids	2	53	69	3250	0.028985507	0.310988693	0.5072554	0.440993264	C00249 C16526	https://www.genome.jp/kegg-bin/show_pathway?hsa01040/hsa:C00249%09red/hsa:C16526%09red
hsa04714	Thermogenesis	1	53	23	3250	0.043478261	0.315772954	0.50062507	0.440993264	C00116	https://www.genome.jp/kegg-bin/show_pathway?hsa04714/hsa:C00116%09red
hsa00220	Arginine biosynthesis	1	53	23	3250	0.043478261	0.315772954	0.50062507	0.440993264	C00624	https://www.genome.jp/kegg-bin/show_pathway?hsa00220/hsa:C00624%09red
hsa00130	Ubiquinone and other terpenoid-quinone biosynthesis	2	53	71	3250	0.028169014	0.323117582	0.49063941	0.441464373	C00082 C02477	https://www.genome.jp/kegg-bin/show_pathway?hsa00130/hsa:C00082%09red/hsa:C02477%09red
hsa05200	Pathways in cancer	1	53	24	3250	0.041666667	0.327010647	0.485438108	0.441464373	C00187	https://www.genome.jp/kegg-bin/show_pathway?hsa05200/hsa:C00187%09red
hsa04742	Taste transduction	1	53	25	3250	0.04	0.338067198	0.470996966	0.441668437	C00158	https://www.genome.jp/kegg-bin/show_pathway?hsa04742/hsa:C00158%09red
hsa00600	Sphingolipid metabolism	1	53	25	3250	0.04	0.338067198	0.470996966	0.441668437	C12144	https://www.genome.jp/kegg-bin/show_pathway?hsa00600/hsa:C12144%09red
hsa00450	Selenocompound metabolism	1	53	27	3250	0.037037037	0.359648293	0.444121996	0.454309895	C00041	https://www.genome.jp/kegg-bin/show_pathway?hsa00450/hsa:C00041%09red
hsa04216	Ferroptosis	1	53	27	3250	0.037037037	0.359648293	0.444121996	0.454309895	C02477	https://www.genome.jp/kegg-bin/show_pathway?hsa04216/hsa:C02477%09red
hsa00330	Arginine and proline metabolism	2	53	78	3250	0.025641026	0.365129331	0.437553278	0.454309895	C00750 C00791	https://www.genome.jp/kegg-bin/show_pathway?hsa00330/hsa:C00750%09red/hsa:C00791%09red
hsa01523	Antifolate resistance	1	53	28	3250	0.035714286	0.370178433	0.431588887	0.454309895	C00073	https://www.genome.jp/kegg-bin/show_pathway?hsa01523/hsa:C00073%09red
hsa04070	Phosphatidylinositol signaling system	1	53	29	3250	0.034482759	0.380538626	0.419601254	0.46005416	C03546	https://www.genome.jp/kegg-bin/show_pathway?hsa04070/hsa:C03546%09red
hsa00730	Thiamine metabolism	1	53	31	3250	0.032258065	0.400759896	0.397115745	0.470457269	C00082	https://www.genome.jp/kegg-bin/show_pathway?hsa00730/hsa:C00082%09red
hsa00010	Glycolysis / Gluconeogenesis	1	53	31	3250	0.032258065	0.400759896	0.397115745	0.470457269	C00103	https://www.genome.jp/kegg-bin/show_pathway?hsa00010/hsa:C00103%09red
hsa00500	Starch and sucrose metabolism	1	53	37	3250	0.027027027	0.457616391	0.339498429	0.529527538	C00103	https://www.genome.jp/kegg-bin/show_pathway?hsa00500/hsa:C00103%09red
hsa00062	Fatty acid elongation	1	53	38	3250	0.026315789	0.466563272	0.331089452	0.532276409	C00249	https://www.genome.jp/kegg-bin/show_pathway?hsa00062/hsa:C00249%09red
hsa04080	Neuroactive ligand-receptor interaction	1	53	40	3250	0.025	0.484024806	0.315132381	0.544527906	C00245	https://www.genome.jp/kegg-bin/show_pathway?hsa04080/hsa:C00245%09red
hsa00520	Amino sugar and nucleotide sugar metabolism	2	53	108	3250	0.018518519	0.531268289	0.274686106	0.589489472	C00043 C00103	https://www.genome.jp/kegg-bin/show_pathway?hsa00520/hsa:C00043%09red/hsa:C00103%09red
hsa00562	Inositol phosphate metabolism	1	53	47	3250	0.021276596	0.540835919	0.266934473	0.591996074	C03546	https://www.genome.jp/kegg-bin/show_pathway?hsa00562/hsa:C03546%09red
hsa00071	Fatty acid degradation	1	53	50	3250	0.02	0.563261071	0.249290263	0.608321957	C00249	https://www.genome.jp/kegg-bin/show_pathway?hsa00071/hsa:C00249%09red
hsa00564	Glycerophospholipid metabolism	1	53	52	3250	0.019230769	0.577610467	0.238364945	0.615611156	C04308	https://www.genome.jp/kegg-bin/show_pathway?hsa00564/hsa:C04308%09red
hsa00051	Fructose and mannose metabolism	1	53	54	3250	0.018518519	0.591497011	0.228047446	0.622224128	C00794	https://www.genome.jp/kegg-bin/show_pathway?hsa00051/hsa:C00794%09red
hsa00061	Fatty acid biosynthesis	1	53	56	3250	0.017857143	0.604935353	0.218291034	0.628202098	C00249	https://www.genome.jp/kegg-bin/show_pathway?hsa00061/hsa:C00249%09red
hsa00350	Tyrosine metabolism	1	53	78	3250	0.012820513	0.726937347	0.138503018	0.745340824	C00082	https://www.genome.jp/kegg-bin/show_pathway?hsa00350/hsa:C00082%09red
hsa00524	Neomycin, kanamycin and gentamicin biosynthesis	1	53	81	3250	0.012345679	0.74040166	0.130532616	0.749656681	C00043	https://www.genome.jp/kegg-bin/show_pathway?hsa00524/hsa:C00043%09red
hsa00140	Steroid hormone biosynthesis	1	53	99	3250	0.01010101	0.808531332	0.092303146	0.808531332	C00187	https://www.genome.jp/kegg-bin/show_pathway?hsa00140/hsa:C00187%09red

Supplement 5C: Metabolic bubble map analysis evidence for metabolic pathways of Group1-2 and Group3-4. Metabolic pathway analysis was performed on all samples of control group and experimental group. P-value is the significance of the metabolic signaling pathway.
